# Finding Associations among Histone Modifications Using Sparse Partial Correlation Networks

**DOI:** 10.1371/journal.pcbi.1003168

**Published:** 2013-09-05

**Authors:** Julia Lasserre, Ho-Ryun Chung, Martin Vingron

**Affiliations:** 1 Computational Molecular Biology, MPI for Molecular Genetics, Berlin, Germany; 2 Otto-Warburg Laboratory, MPI for Molecular Genetics, Berlin, Germany; ETH Zürich, Switzerland

## Abstract

Histone modifications are known to play an important role in the regulation of transcription. While individual modifications have received much attention in genome-wide analyses, little is known about their relationships. Some authors have built Bayesian networks of modifications, however most often they have used discretized data, and relied on unrealistic assumptions such as the absence of feedback mechanisms or hidden confounding factors. Here, we propose to infer undirected networks based on partial correlations between histone modifications. Within the partial correlation framework, correlations among two variables are controlled for associations induced by the other variables. Partial correlation networks thus focus on direct associations of histone modifications. We apply this methodology to data in CD4+ cells. The resulting network is well supported by common knowledge. When pairs of modifications show a large difference between their correlation and their partial correlation, a potential confounding factor is identified and provided as explanation. Data from different cell types (IMR90, H1) is also exploited in the analysis to assess the stability of the networks. The results are remarkably similar across cell types. Based on this observation, the networks from the three cell types are integrated into a consensus network to increase robustness. The data and the results discussed in the manuscript can be found, together with code, on http://spcn.molgen.mpg.de/index.html.

## Author Summary

Nucleosomes are protein complexes around which the DNA is wrapped for compactness. They are made of histone proteins that can be post-translationally modified and these histone modifications can affect the expression of surrounding genes. In the past decade, scientists have developed a strong interest in the part of gene regulation provided by epigenetics, i.e. those heritable characteristics that are not based on the DNA sequence and that can therefore be cell-type-specific, such as histone modifications. Striking patterns about the co-occurrence of modifications have been discovered, leading to the hypothesis that different combinations of modifications lead to different effects. Different histone modifications could act jointly to recruit certain proteins, or be required sequentially, which is reflected in statistical dependencies in measured data. The focus of this article is on building a network that represents the global dependencies by extracting direct associations of histone modifications. We find that, although histone modifications patterns are cell-type specific (modifications may not necessarily appear at the same loci), the dependencies are to a large degree cell-type independent, which is supported by a large overlap of the inferred associations in the networks built for different cell types. We are able to find meaningful associations, both known and novel.

## Introduction

The study of gene regulation is traditionally based on DNA sequence analysis, gene interactions and transcription factor binding events. It has however over the past decade been revolutionized by genome-wide maps of epigenetic marks, specifically DNA methylation and histone modifications. Histone modifications are post-translational modifications of the histone proteins which form nucleosomes by wrapping about 147 base pairs of DNA. These modifications can have effects on biological processes including transcription, DNA repair, splicing, dosage compensation and more [1], [2], either by altering the chromatin structure or by recruiting key proteins [1]. The observation of different histone modifications co-occurring in different contexts has raised the possibility of combinatorial effects and has led to the histone code hypothesis [3], whereby combinations of histone modifications have a biological meaning and lead to distinct downstream effects.

In particular, there has been much evidence for a strong role of histone modifications in the regulation of gene expression [4], [5], not only at promoters and enhancers, but also along the gene body. Many authors have contributed genome-wide pattern analyses of modifications around regulatory regions [6]–[10]. For example, it has been found that acetylation marks generally co-occur with active genes, whereas methylation marks can be associated with active genes or repressed genes, depending on the modified residue. Histone modifications can be clustered according to their average level around promoters into two groups, one group containing active marks and the other repressive marks [7]. Ernst *et al.* [9] used hidden Markov models to extract genome-wide epigenetic states, many of which can be thought of as characterizing the transcriptional process at various positions along the gene body, or different kinds of enhancers, or splicing or heterochromatin, etc. Although it is still unclear whether they are causes or effects of transcription, these observations clearly demonstrate a connection between different combinations of histone marks and different transcription states. For instance, it is well established that promoters carry H3K4me3 and/or H3K27me3 and that actively transcribed genes carry H3K36me3 [11], whereas enhancers are marked by H3K4me1 and H3K27ac [11], [12]. Histone modifications have even been successfully used to determine the presence of regulatory elements such as promoters or enhancers [11], [13]–[17]. Beyond these qualitative findings, a remarkable quantitative relationship with mRNA expression levels has been demonstrated in [18]. However, so far all of these studies deal with co-occurrence but do not provide insights about associations between histone modifications.

In this article, we are interested in building networks of histone modifications. This is a problem that benefits from relatively few variables (histone modifications) and many samples (genomic regions of interest), allowing the use of rigorous statistical methods. In such networks, nodes represent histone modifications, and edges connections between them. The nature of these connections depends on the construction method used to obtain the network. Other authors, again particularly in the context of promoters, could capture associations using Bayesian networks (BNs) of histone modifications [19]–[23]. They aimed at establishing causal links: which modifications are required for the presence of another one. However claims about causality in BNs are controversial [24]–[27], especially in the presence of hidden confounding factors, which occur quite frequently in biological systems. Additionally, BNs do not allow cycles or feedback mechanisms, which seems unrealistic in biological systems.

The ChIP-seq data currently available represents a summary of the epigenome, averaged over many cells. For each histone modification, the read counts represent the average frequency at which it is found in the population of cells. This has three main implications for the interpretation of the edges. Firstly, it is very hard to make any claims about causality, as temporal information is missing. Secondly, discretization of the read counts is less plausible. Even if a histone modification is either present or absent at a specific region in a specific cell, the read counts represent the average over many cells, and discretizing these averages is no longer meaningful. Thirdly, given that only an average picture is available, it can safely be assumed that various states will be represented in the data and will appear in the network. Being in one particular state will mean highlighting relevant associations and downplaying others, but all associations will be present in the same network. In a way, we expect to infer the wiring of the circuit as opposed to the flow in the circuit, i.e. the statics as opposed to the dynamics. Edges can reflect co-occurence, mutual exclusivity, or they can mean that two modifications occur sequentially as part of the same pathway. We cannot distinguish between these scenarios with the data at hand.

An observed correlation between two variables may either reflect a direct association or an induced association that may be due to a mutual association with a third variable. For example, if the lack of sports generates both a drop in fitness and a bad mood, a correlation between the variables 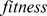 and 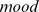 will be observed when, actually, they are only connected through the variable 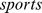 and do not interact otherwise. The third variable (here 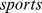) is often referred to as confounding factor. Confounding factors, which can be accountable for part of the associations between other variables, are often presented as a nuisance - experimental techniques for instance may lead to biases that are undesirable confounding factors - however they need not be. For example, expression level is a confounding factor of great interest. In any case, looking at how apparent associations may be explained away can be very insightful.

Let us suppose we have two variables of interest 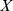 and 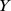. The correlation coefficient is a powerful tool but it cannot distinguish direct associations from those due to confounding factors. The partial correlation coefficient was designed to remedy that very problem [28]. The idea is to subtract from 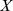 and 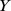 the information contained in a control group of variables 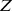 by linearly regressing 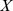 (resp. 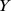) against 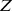, and to keep the residuals 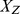 (resp. 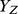). We then compute the correlation between 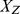 and 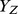. This correlation is called a partial correlation, written 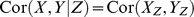 and is a measure of the correlation between 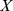 and 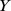 that remains after the explanatory power of 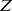 is taken out.

Let us assume we have a set of 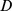 variables 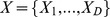, and we compute the correlation matrix 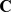 such that 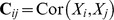. Let 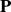 denote the partial correlation matrix (PCM) that contains the pairwise partial correlations, each using as control the remaining variables, i.e. the matrix such that 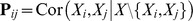. Note that, in this framework, each variable in turn is treated as a confounding factor, regardless of its expected biological relevance. A property of partial correlations is that 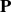 may be obtained by simply inverting, normalizing and negating the correlation matrix 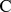 [29]–[32]. This procedure, that we will use throughout the study, is a very fast alternative to the linear regressions. It also shows the involvement of all variables in the computation of 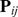 through the inversion step, as opposed to 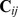 that is only computed on 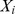 and 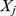.

It is common practice to recover the undirected network connecting these 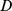 variables by simply building a fully connected network and by removing all edges 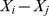 for which 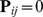 [29]–[32]. This rests on the theoretical grounds that the variables are normally distributed and are linearly related, therefore having 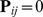 is equivalent to having independence between 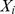 and 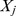 conditioned on the other variables [29]–[32], which is exactly the requirement for the absence of edge in an undirected network. Such networks are therefore referred to as graphical Gaussian models (GGMs) [29]–[32]. In case the true network is Bayesian (i.e. directed and acyclic) then the GGM will contain the original edges and will connect the parents of a same child. GGMs provide a simple and efficient method, whereby networks can be built in just a few seconds. They have been successfully applied to infer gene regulatory networks, even in the presence of small sample size, and a short review of these applications can be found in [33].

In this study, we propose to focus on edges that represent direct dependencies. We want to draw edges between histone modifications that are directly linked in a pathway or that act together, i.e. whose association cannot solely be explained by confounding factors. We build on GGMs, and put forward a robust method to compute sparse partial correlation networks (SPCNs). To the best of our knowledge, PCNs have not yet been applied to histone modifications. In contrast to gene regulatory networks, here the sample size is very large and the variables are few. Formally, partial correlations require normal distributions. In our work this need is overcome and outliers accounted for by rank-transforming the input data. Sparseness is achieved via a cross-validation scheme. Our SPCNs reveal edges that are symptomatic of direct associations, mutual exclusivities, direct edges in a pathway, indirect edges where the intermediate variable(s) are not available, or collaborative work to produce a third variable.

Zhao's group was one of the first to produce genome-wide profiles for a large number of histone modifications, they did so in CD4+ cells [6], [7]. In the meantime, several other groups have contributed to the Roadmap Epigenomics project [34], a database that now contains data for varying numbers of histone modifications in different cell types. Based on this data, the cell types with the largest number of histone modifications were chosen: CD4+, IMR90 and H1. CD4+ cells are lymphocytes (white blood cells), they are part of our immune system. IMR90 cells are fibroblasts (cells involved in the synthesis of tissues' external structure) in the lung, and H1 cells are embryonic stem cells. 21 histone modifications are available for all three cell types, we keep only those. Histone modification data is obtained via ChIP-seq experiments, so openness of the chromatin is a potential confounding factor to include in the analysis via DNaseIHS, which marks the hypersensitivity of the DNA to the enzyme DNaseI. The relationship of histone modifications to mRNA levels is of particular interest because of the role of histone modifications in transcription, so mRNA data is included. We look at the amounts of ChIP-seq reads for these 23 variables in the [−2000,+2000] around the transcription start sites (TSSs) of known genes, and at the amounts of RNA-seq reads in the exons of those genes. Antibodies can also play a role as confounding factors (because of their cross-reactivity), and may also vary from experiment to experiment. Antibodies are an interesting case because, although they are not semantically “hidden” (we know which ones are used and we know they can cross-react and act as confounding factors), they are technically hidden since we do not know how they cross-react as no data is available. However, we can build a table of cross-reactions and look it up as a possible source of explanation for links between histone modifications. Details about data collection and antibody can be found in Materials and Methods.

## Results

### Sparse partial correlation networks (SPCNs)

**Figure 1:**
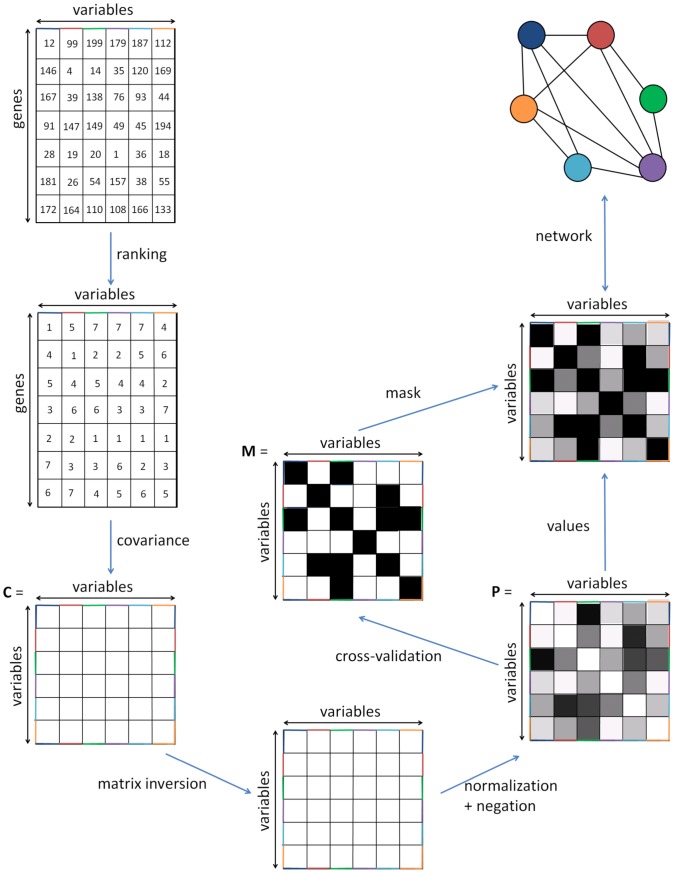
Global view of the algorithm. The data matrix is rank-transformed, and the covariance matrix 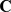 is computed. 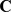 is then inverted, negated and normalized as described in Materials and Methods to obtain the partial correlation matrix 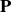. Cross-validation is performed to build a mask 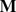 which is applied on 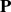 to give a sparse partial correlation network.

We modify GGMs in two respects: first by rank-transforming the input data, and second by enforcing sparseness via a cross-validation scheme. A global view of the algorithm is shown in Figure 1. Precision is favored over completeness: an edge is only found in a network if it is strongly supported by the data. Therefore interpreting edges is favored over interpreting the lack thereof. Details about the computation of the PCMs, the p-values and the q-values can be found in Materials and Methods.

#### Rank-transformation of the data

**Figure 2:**
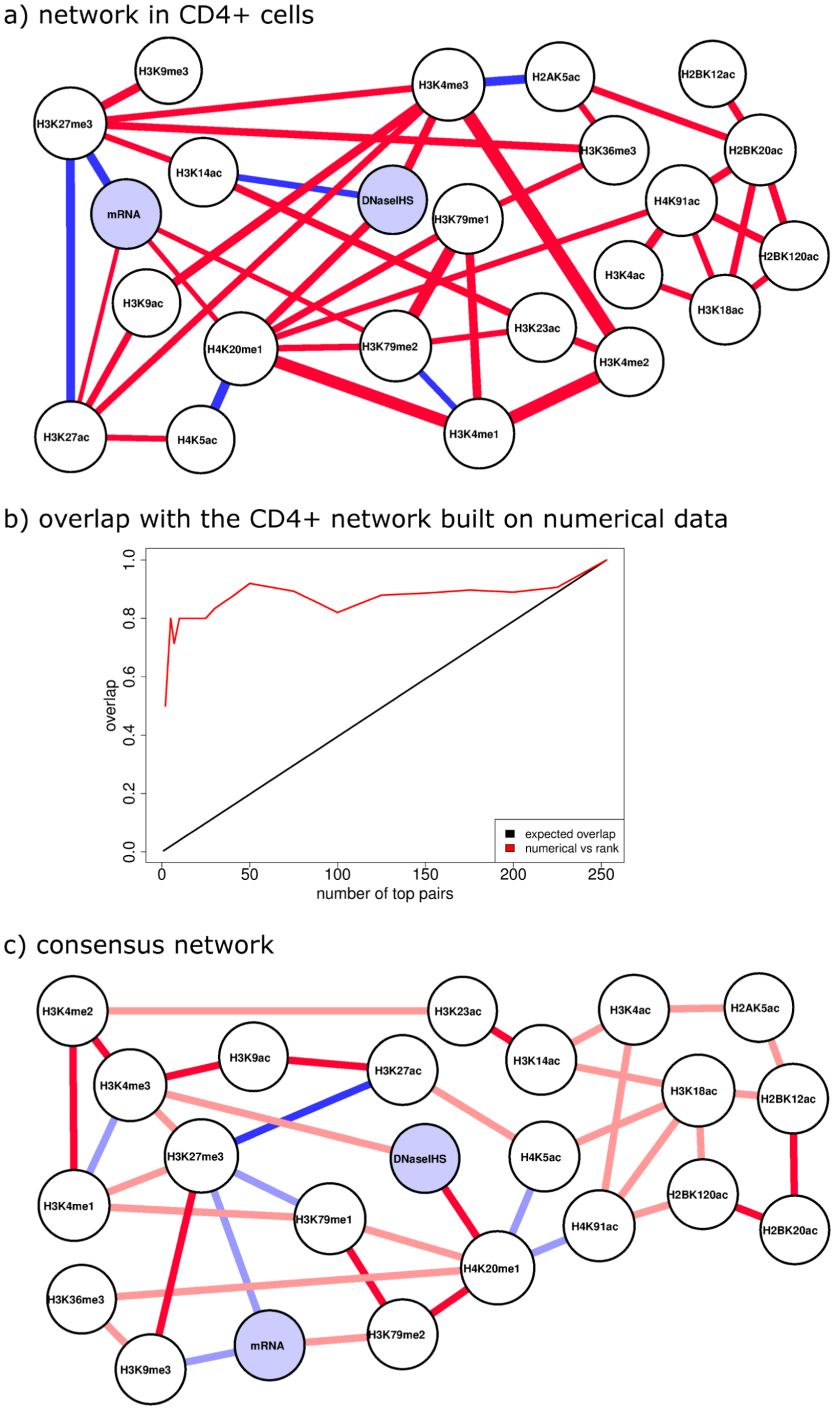
a) Network in CD4+ cells. Blue edges represent negative partial correlations, while red edges represent positive partial correlations. **b) Overlap with the CD4+ network built on numerical data.** Numerical data means that the counts are taken to the log instead of being ranked, so quantitative information is preserved. There is very little difference between the two networks. **c) Consensus network.** Blue edges represent negative partial correlations, while red edges represent positive partial correlations. Bright edges (blue and red) represent edges that are common to all networks, light edges (light blue and pink) are found in two networks out of three. Any blue means a negative partial correlation, while red or pink means a positive partial correlation.

Most histone modifications data is multimodal (see Text S1 Section 4). This observation could imply that discretizing the data is the solution. However relationships between histone modifications go beyond mode associations, they also exist within the modes, which discretized data cannot account for. Results on discretized data are discussed in Text S1 Section 5.4. Instead, Tto render PCNs less sensitive to the distribution of the data and to account for outliers, data is rank-transformed: for each data matrix of interest, and for each variable in that matrix, the entries corresponding to the levels of this variable in various genes are ranked and replaced by their rank [35]. Rank data is uniformly distributed over 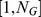, 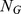 being the number of genes under consideration. However with so many genes at hand it may be approximated with a very wide Gaussian. By applying the rank-transformation, statistical power is partly sacrificed for robustness. Rank-transformation provides a reference transformation that can be used by anyone on any data, which is useful as every lab has its own normalizing method. Ranking may not always be a good idea, depending on how the data looks like. But histone modifications have monotonic relationships and, in this setting, ranking may lose the modes but it does not change the existence or non-existence of the relationships. What we measure in rank space is how close two variables are from being a monotonic transformation of one another, as opposed to a linear transformation. In our simulations, PCNs on rank data perform well, as discussed in Text S1 Section 5. In fact, as shown in Figure 2b and Text S1 Section 5.3, there is little difference with PCNs on numerical data, whether on simulations or on real data, which shows that the underlying structure is not modified. Again, this result stems from the monotonic properties of histone modification data and may not be extended to any dataset without caution. Indeed histone modifications data is not Gaussian, most distributions are multimodal (see Text S1 Section 4). Upon rank-transforming the data, the modes are lost. However here it is acceptable since the relationships between variables go beyond mode associations, which would then call for discretizing rather than ranking. Instead relationships also exist within the modes.

#### Sparseness through cross-validation

Our dataset enjoys a very large number of samples, therefore the q-values of all partial correlation coefficients will be low and all entries in the PCM 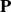 will be considered significant, regardless of their biological relevance [36]. A classical significance threshold can therefore not be used here. Instead, we use the prediction error to produce a mask for 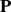. The dataset is split between training and test set, and a sparse partial correlation matrix 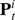 is computed on the training set using a q-value threshold 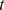. For each variable 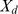, we take as co-variables all of those that have a non-zero entry in the 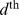 column of 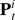, and build a linear regressor for 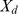 on the training set using as predictors the co-variables only. The predictions 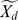 of the linear model on the test set lead to an estimate of the error: 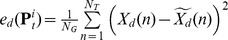, where 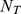 is the number of test data points. The estimates for all of the 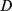 variables are then averaged to give 
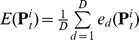
. In case of limited amounts of data, this approach would be self-sufficient as 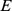 would decrease upon adding the first top edges to the point of overfitting, and would then increase again. Therefore we would simply have to pick the threshold that minimises 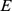. However here, given the large amount of data, 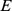 increases continuously with sparseness (as the threshold 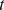 decreases), therefore we pick the lowest threshold 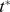 such that 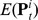 does not exceed the minimum error 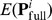 by more than 10% of the difference between 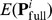 (minimum error) and 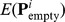 (maximum error). This allows to obtain a sparse matrix 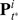 that performs reasonably well compared to the full matrix. The operation is repeated using 10-fold cross-validation (i.e. with 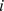 varying from 1 to 10). The 10 resulting sparse matrices are then combined to produce a mask for 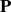: to be kept, an edge has to be found at least 7 times out of 10. Note that, again due to the large amount of data, the 10 sparse matrices are fundamentally very similar and setting the threshold to 5 or to 10 would make very little difference. It does help however to discard aberrant edges that appear only once. In the case of reduced amounts of data, the threshold would be more critical.

### From correlations to partial correlations: Explaining away

“Explaining away” in machine learning is “a common pattern of reasoning in which the confirmation of one cause of an observed event reduces the need to invoke alternative causes” [37]. We take over this concept and translate it into our own context. A connection between 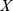 and 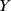 is explained away by 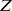 when 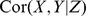 is negligible compared with 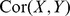, because we assume that 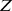 was the main cause of the apparent connection between 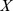 and 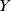 and that therefore the need to find further causes is alleviated.

When controlling for confounding factors, the partial correlation coefficient 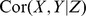 is substituted to the correlation coefficients 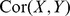 and the difference can be very large. 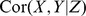 is generally smaller (in terms of absolute value) as it is explained away by the control variables, but it can also be greater as control variables tie 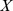 and 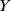 together. For example, if 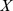 and 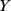 are independent co-parents of 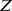 such that 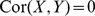, they become dependent upon conditioning on 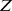, such that 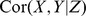 may be different from 0. We would like to know which variables are responsible for most of the change from 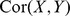 to 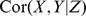. Running an exhaustive search on combinations of about 20 variables is neither possible nor desirable. Instead we condition 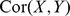 on a single variable 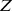. We repeat the operation for every possible 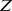 in the dataset and identify the 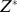 that leads to the biggest discrepancy between 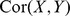 and 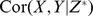, i.e. the control variable that has the highest impact on the correlation. The impact of all variables is shown for some pairs in 

### Stability across cell types

It needs to be established that networks remain stable upon using input data from different experiments or from different cell types. To this end, we define an index of overlap between 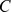 PCMs, based on the ranking of the entries which represent the associations between pairs of variables. For each PCM 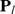 (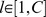), the pairs of variables 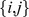 are ranked by increasing q-values and the first 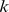 pairs (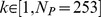) are stored in a list. The number of pairs that occur in all 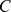 lists divided by 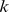 is a measure of the similarity between all the 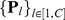 when 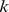 pairs are considered. Results are presented in plots where 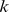 varies from 1 to 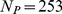. The overlap expected at random depends on the number of matrices being compared 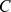 and on the number of pairs being examined 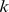. It is easily computed, as seen in Materials and Methods. For 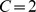, it follows a hypergeometric distribution, and therefore p-values are directly available.

#### Expected variability across experiments

In order to better assess the stability of the results across cell types, the variability that can be observed across experiments needs quantifying. To that end, H1 data from the ENCODE project [38] was downloaded for each histone modification that was also in the data previously described. The web addresses of the experiments that were downloaded can be found in Text S1 Section 1.3. The variables common to all four datasets (CD4+, IMR90 and both H1) were used to compute a PCM for H1 Roadmap data, and a PCM for H1 ENCODE data. The variability between the two will give a good idea of the variability of the data across experiments.

**Figure 3:**
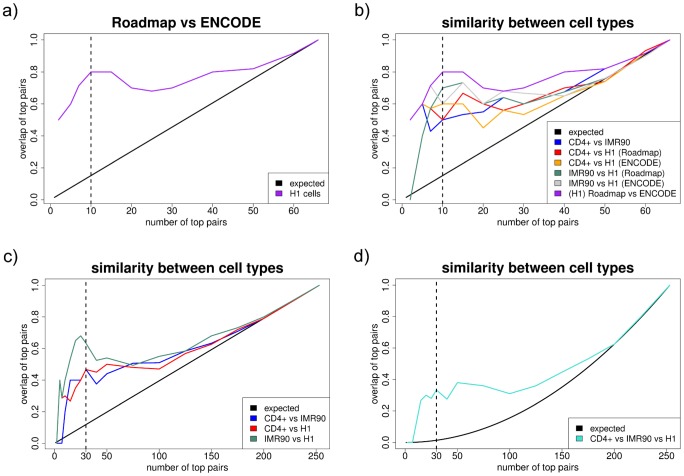
Similarity between experiments and cell types. All plots have the same construction. The x-axis shows the number of top pairs that are considered 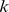. The y-axis shows the proportion of these pairs that are found in the two lists being compared (three lists for subplot d), as an estimate of the similarity between partial correlation matrices. **a**) Similarity - within H1 cells - between Roadmap and ENCODE data, i.e. between experiments, using variables available in all datasets only. For the top 10 pairs, the overlap is 80% (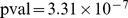). **b**) Similarity between two cell types and between experiments, using variables available in all datasets only. For the top 10 pairs, the overlap is 50% between CD4+ and IMR90, and between CD4+ and H1 Roadmap (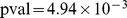), 60% between CD4+ and H1 ENCODE, and between IMR90 and H1 ENCODE (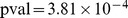), and 70% between IMR90 and H1 Roadmap (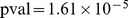). **c**) Similarity between two cell types for the 23 variables used throughout the study. For the top 30 pairs, the overlap is 47% between CD4+ and IMR90, and between CD4+ and H1 (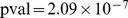), and 63% between IMR90 and H1 (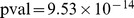). **d**) Similarity between all three cell types for the 23 variables used throughout the study. For the top 30 pairs, the overlap is 33% (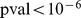).

The procedure described above was applied on the PCMs obtained for Roadmap and ENCODE data in H1 cells, the results are shown in Figure 3a. On the x-axis is the number 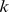 of top pairs, on the y-axis the proportion of these top pairs found in both lists. The similarity is far from random: for the top 10 pairs, 8 are common to both lists (hypergeometric test, 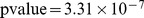). It shall serve as a reliable baseline for what to expect when comparing PCMs across cell types. In particular, it is nowhere near 100% and indicates a high level of experimental noise.

#### Similar partial correlations across cell types

The next step is to repeat the procedure for the PCMs obtained for the same set of histone modifications in CD4+ and IMR90 cells. We compared all pairs of PCMs (CD4+ vs IMR90, CD4+ vs H1 (Roadmap), CD4+ vs H1 (ENCODE), IMR90 vs H1 (Roadmap), IMR90 vs H1 (ENCODE) and H1 (Roadmap) vs H1 (ENCODE) on the same plot in Figure 3b. For the top 10 pairs, 5 are common to CD4+ and IMR90, and to CD4+ and H1 Roadmap (hypergeometric test, 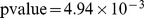), 6 are common to CD4+ and H1 ENCODE, and to IMR90 and H1 ENCODE (hypergeometric test, 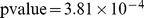), and 7 are common to IMR90 and H1 Roadmap (hypergeometric test, 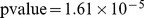). Although the similarity across cell types is lower than within H1 (Roadmap and ENCODE), it is in fact comparable. This shows that the signal is stable across cell types, and that the variability can largely be attributed to experimental noise.

Sonication-ChIP-seq, or MNase-ChIP-seq, may be biased and cause fake links between histone modifications due to the common approach to fragmenting DNA. MNase-seq (i.e. MNase digestion of chromatin without ChIP) and Input represent data that can account for these biases, and can be seen as ChIP-seq controls. To check whether these ChIP-seq controls can explain some of the gap between the variability across experiments and the variability between cell types, MNase was added to the CD4+ dataset (generated with MNase-ChIP-seq) and Input to the IMR90 and H1 datasets (generated with sonication-ChIP-seq). The plot in Text S1 Section 6.1 compares the overlap between two cell types, in the presence and absence of ChIP-seq control. There is no fundamental change.

Figure 3c shows the overlap of the matrices between the three pairs of cell types, when using all the 23 variables (i.e. ignoring ENCODE data). Here again, the overlap is clearly higher than expected by chance for important edges: for the top 30 pairs, 14 are common to CD4+ and IMR90, and to CD4+ and H1 (hypergeometric test, 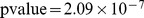), and 19 are common to IMR90 and H1 (hypergeometric test, 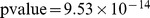). Moreover, Figure 3d shows the overlap between all three matrices. For the top 30 pairs, 10 are common to all cell types (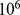 simulations under the null model, 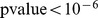). This confirms the existence of a common core. The ChIP-seq controls were also performed to see if the overlap could increase (see Text S1 Section 6.2), but no change was observed.

### Network of histone modifications in CD4+ cells

We now turn to a detailed analysis of the CD4+ network. Note that, the data containing 23 variables, the SPCN has 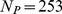 edges maximum. The resulting network is shown in Figure 2a, all the partial correlation coefficients, their q-values and the mask are given in Text S1 Section 7.

Looking at edges around mRNA, we find it is negatively connected to H3K27me3 (a mark of repression) and positively to H3K27ac (a mark of activation), H3K79me2 and H4K20me1 (marks of elongation), which have been, with the exception of H3K27me3, found to be important in predicting expression in CD4+ cells [18]. Interestingly, H3K36me3 has no link to mRNA, in line with [18]. The scatter plots in Text S1 Section 9.1 confirm the lack of relationship. Note that there is no standard correlation either. The data for H3K36me3 is not abundant, very few reads map to the regions of interest. This could come from H3K36me3's preference for exons [39]. Indeed exons are only a small part of the studied region, as shown in Text S1 Section 3, so the lack of connection to expression could be due to poor data, it is hard to tell.

Expected connections are numerous, such as the negative link between H3K27ac and H3K27me3. These two histone modifications are by nature mutually exclusive, and therefore need not be explained by any other histone modification. The strong connections between the various methylation states of H3K4, with H3K4me2 in between, are explained by the fact that these different methylation states are coupled by bidirectional links from H3K4me1 to H3K4me2 and to H3K4me3. Alternatively, it can be explained by antibody cross-reactivity, but it may not be explained by any other histone modification. Connections between DNaseIHS and H3K4me3 and H4K20me1 reflect the need for open chromatin to have transcription.

Finding expected associations is a requirement, however it is more interesting to find unexpected connections. H3K27me3 and H3K9me3 are positively associated (see scatter plots in Text S1 Section 9.2). They have been thought to be mutually exclusive, H3K9me3 encoding constitutive heterochromatin, H3K27me3 facultative heterochromatin. Both would act as repressors but as part of two different processes (involving the PRC1/2 complex for H3K27me3 and the HP1 proteins for H3K9me3), that have been assumed mutually exclusive [40]. Clearly it is not the case here. It has been found that SUZ12, which is part of PRC2 and involved in setting H3K27me3, promotes H3K9 methylation [41], giving a straightforward explanation for our finding. The negative edge between H3K79me2 and H3K4me1 is puzzling given that they are two marks associated with transcription, and that the trend is mostly tue in active genes (see scatter plots in Text S1 Section 9.3). However a possible explanation is that H2BK120ub1, which is required both for the production of H3K4me2/3 and of H3K79me1/2 [42], acts as hidden confounding factor.

Some expected edges exist albeit with an unexpected sign. In particular, H3K4me3 and H3K36me3, associated with initiation and elongation, are positively linked to the repressive mark H3K27me3 (see scatter plots in Text S1 Section 9.4). In fact, for high levels of H3K27me3, this trend already exists in the raw data. This may indicate that some promoters cycle between the repressed H3K27me3 state and the active H3K4me3/H3K36me3 state. The cycling idea of epigenetic states is not without precedent. It has been shown that the estrogen receptor target TFF1 is cyclically methylated and demethylated [43], [44]. In some cells promoters are active (H3K4me3), in some cells they are repressed (H3K27me3), and in some cells they may be bivalent (H3K4me3 AND H3K27me3). All we measure is the population average. If these fluctuations are stochastic, we expect no correlation. However if promoters can move from being active (H3K4me3) to being inactive (H3K27me3) in a regulated manner, then we expect a positive correlation. This could be due to the cell cycle, e.g. promoters get active during S-phase and are rendered inactive thereafter [45]. When looking at the scatter plots in Text S1 Section 9.4, the correlation seems to come from repressed genes, and a little bit from bivalent genes, supporting this hypothesis.

Another example is the negative link between H4K20me1 and H4K5ac (see scatter plots in Text S1 Section 9.5), which seems at first glance counter-intuitive because H4K20me1 is positively linked to expression and acetylations are generally thought to be associated with transcription. This apparent paradox can be resolved by the following reasoning: H4K20me1 is mainly associated with transcription elongation, while acetylations are heavily enriched around the promoter. It has been shown in Drosophila that H4K20me1 recruits the factor RPD3/HDAC1, leading to the deacetylation of H4K [46]. Thus it seems that H4K20me1 helps to prevent cryptic initiation in the transcribed gene body.

Since mechanisms are to a large degree cell-type-independent, the precision and robustness of the results can be increased by integrating information from all available cell types. A SPCN is created for each cell type. Figure 2bc shows the consensus network which contains only those edges that are found in at least two cell-type-specific SPCNs. Light blue edges show negative associations that are found in two cell types, blue edges negative associations found in all three cell types. Pink edges show positive associations that are found in two cell types, red edges positive associations found in all three cell types. It looks very similar to the CD4+ SPCN in Figure 2a. Important associations such as mRNA-H3K27me3, mRNA-H3K79me2, DNaseIHS-H3K4me3, DNaseIHS-H4K20me1 and H3K27ac-H3K27me3 are conserved across cell types. Surprising connection such as H3K27me3-H3K9me3 and H4K20me1-H4K5ac are also stable. The strong connection between H3K4me1 and H4K20me1 is only found in CD4+.

Some of the edges that are common to all networks (marked in bright red and blue) are of particular interest. The antibody table in Text S1 Section 2 (see Materials and Methods) shows that there is antibody cross-reactivity for H3K4's various methylations and for H3K79me1/2. The edges may reflect biologically meaningful associations but may (also or instead) be due to cross-reactions. H3K23ac's antibody reacts with H3K14ac, H3K18ac's with H4K5ac, and H3K27ac's with H3K9ac, which explains partially these three connections. The group H2BK12/20/120ac remains unexplained, however it is plausible that it may be the result of unreported antibody cross-reactions. Other edges that may be explained by antibody cross-reactivity are H4K5ac-H3K27ac and H4K5ac-H3K18ac as well as H3K14ac-H3K18ac.

### Effect matrix of histone modifications in CD4+ cells

**Figure 4:**
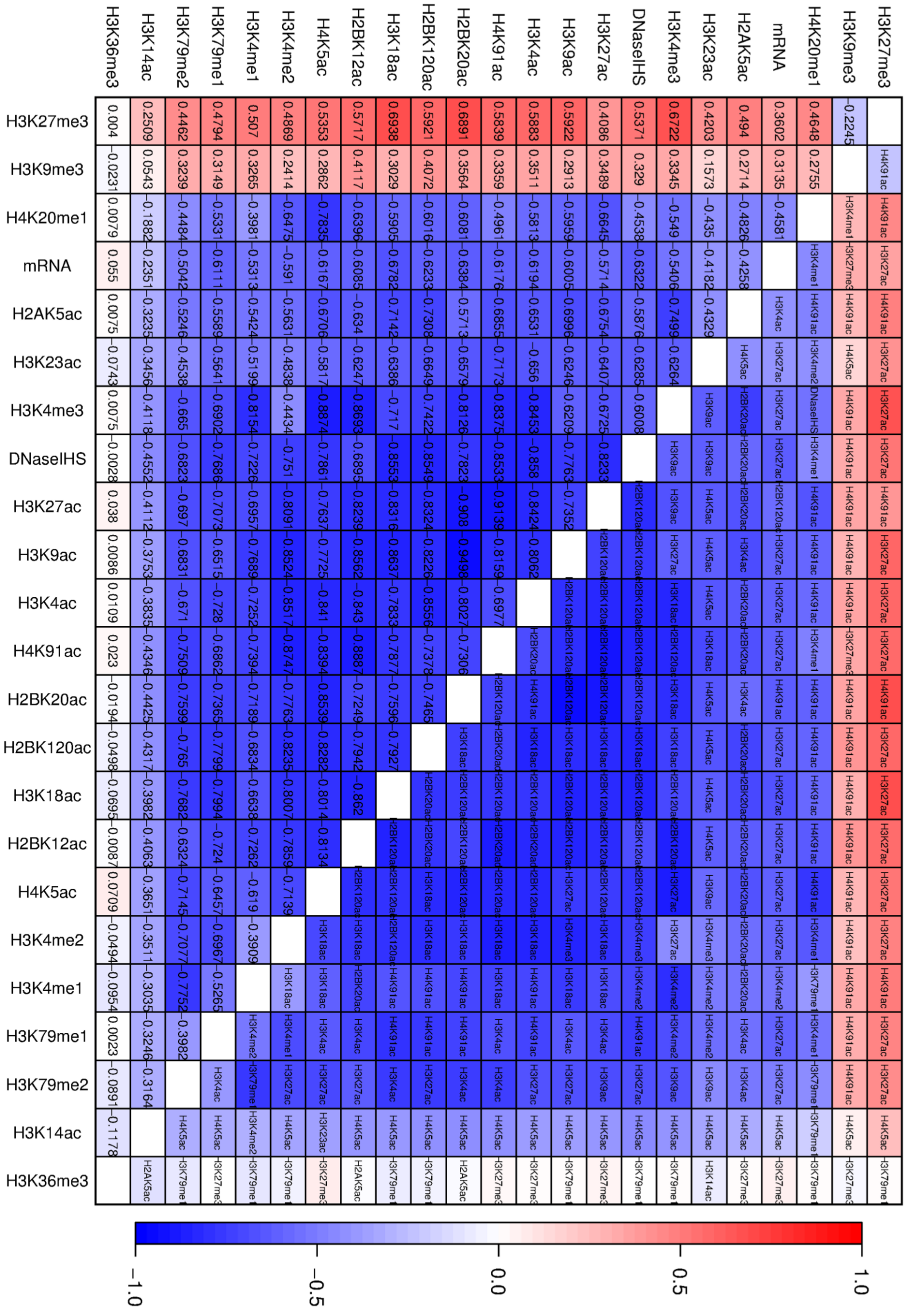
Effect matrix in CD4+ cells. The color code represents the difference between the partial correlation coefficient 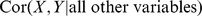 and the correlation coefficient 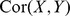. The difference 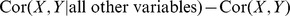 is given in the lower cell of the corresponding pair. The variable 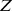 that has the largest effect 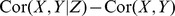 is written in the upper cell of the corresponding pair.

The explaining away procedure was applied. Text S1 Section 10 shows some of the plots that are obtained for all the edges of interest. Figure 4 summarizes the critical information into one matrix. The colors give the magnitude of the differences between 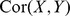 and 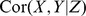. If zooming in is available, the numbers on the lower part of the diagonal give the actual difference, and the text on the upper part of the diagonal gives the histone modification that has the most incidence on 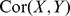.

Partial correlations work in such a way that, in order to explain the correlation between 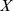 and 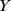, it is sufficient that a control variable 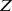 explain 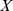. The variable with the most impact then says something about 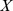 regardless of 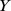. Symptomatic of this scenario, the first explanatory variable is then often the same along the column of the matrix corresponding to 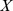. For example, in the column associated with H3K27me3, H3K27ac is very often the most influential variable. It can be assumed that H3K27ac explains H3K27me3 and therefore leads to the loss of correlation between H3K27me3 and other variables. H4K5ac seems to explain H3K14ac. This may be due to antibody cross-reactivity, as H4K5ac is often seen in H3K23's column, and H3K14ac's and H3K23ac's antibodies are known to cross-react.

An interesting example that shows how well this procedure works is the pair H3K4me1 and H3K4me3. After glancing at Text S1 Section 8.1 or after zooming into Figure 4, it can be seen that the variable most responsible for the correlation is H3K4me2. This makes a lot of sense biologically, as H3K4me2 is an intermediate state of methylation. Another example is the correlation between mRNA and H3K4me3, which seems to be largely explained by H3K27ac. This maybe due to the fact that H3K4me3 recruits the SAGA complex required for acetylation [47] which puts H3K27ac, which in turn is predictive of mRNA levels, as was seen in [18]. The relationship between H3K4me3 and H4K20me1 is fully explained by DNaseIHS. One possible reason for this is that chromatin openness favors transcription, thereby explaining H3K4me3. The role of H4K20me1 in HDAC recruitment has been demonstrated in the context of chromatin reassembly [46]. Thus it seems that transcription may lead to higher histone turnover, which results in higher levels of H4K20me1.

Similarly to the networks, a consensus effect matrix is shown in Text S1 Section 8.4. It is surprising to see how well the effect of partial correlation and the explanatory variables are conserved across cell types. Indeed, out of 21 possible variables that are all correlated, in most cases the same one comes out in at least two cell types.

## Discussion

We put forward SPCNs, a fast and robust tool, to construct undirected networks of histone modifications. By definition SPCNs can handle continuous data. Moreover they contain all relevant links, and allow for cycles and symmetric relationships. Edges in a SPCN may be seen as controlled associations, where the link between two variables is only established after controlling for potential confounding factors (the other variables at hand). We believe they are the perfect tool for our purposes. The algorithm is designed to maintain a high precision level in the reconstruction of the networks. To be present, an edge must appear in 7 out of 10 sub SPCMs, i.e. be highly supported by the data. Some edges may be missed, and the lack of edges must be carefully interpreted, however given that only 10% of the maximal drop in performance is allowed, we believe that most contributing edges are recovered, and that the lack of edges mainly corresponds to the lack of relevant associations.

We used the availability of data from different experiments and different cell types to our advantage and quantified the variability that could be expected. Firstly, it is interesting to note that the variability across experiments, for the same cell type, is not low. This tends to show that biological data is difficult to reproduce, that results should be interpreted with care, and that evidence may not be overwhelming even though a phenomenon is true. Here, the cell type is the same so it is true that the mechanisms should be the same, yet the evidence is not as high as one might have expected. Secondly, the variability across cell types is marginally higher than the one across experiments, showing that the networks are stable across cell types, and that the variability is mostly due to experimental noise. This last observation is a significant result. Histone-modifications-related mechanisms are often assumed to be the same in all cell types, but it is not systematically checked. Our simulations show that meachanisms are strikingly similar across cell types, almost as similar as two different experiments in the same cell type.

Gathering information on antibody cross-reactivity was difficult but it proved insightful as it revealed important biases in the data. In particular, different methylation states, such as H3K4me1/H3K4me2/H3K4me3 or H3K79me1/H3K79me2, are difficult to distinguish. The edges between such histone modifications may be biologically relevant or/and due to antibodies' lack of specificity, probably both, it is impossible to tell with the data at hand. A similar phenomenon was observed for acetylations. This ought to be a warning for the community. Antibodies are too trusted in many ChIP-seq studies. Instead cross-reactivities should be documented and biases reported when appropriate. In fact, cross-reaction studies are missing for many antibodies, and biases may be more important than we think.

The SPCN gives a global view of the associations between histone modifications, however this view assumes a closed environment containing only the variables in the network. This is an intrinsic limitation of the method. If the set of variables is increased, the new network will not necessarily contain the previous one, all edges might be affected. How much they might be affected depends on the relevance of the variables that are introduced, and on the number of these variables. This makes the network very hard to test experimentally, as the presence of other variables in the cell will make the network by definition obsolete. However such assumptions are not new in biology, where subsets of variables are often chosen, and consequently studied as if they were isolated from the rest of the world.

The effect matrix on the other hand gives a detailed view of what partial correlation does. It shows the difference between the correlation and the partial correlation conditioned on all other variables. In particular, it allows to see which variable causes the highest difference between 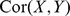 and 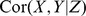. This is of high biological interest, not only because it identifies potential hidden interactions, but also because such effects can be in principle verified experimentally.

Associations of histone modifications are interesting as a first step to understanding their relations. However their connections are not physical and therefore remain abstract. Edges in a SPCN are as direct as possible given the variables at hand, but they can most probably be explained away by enzymes or proteins that float around and provide a physical interface for histone modifications, in particular chromatin modifiers. The next step is therefore to include data for such proteins. Ram *et al.* have now produced data for chromatin regulators [48]. Including them in the network and particularly in the effect matrix would allow to gain much deeper insight into the physical mechanisms. Further steps should also include transcription factors, and various genomic regions, such as proximal promoters and enhancers.

## Materials and Methods

### Data

#### Data collection

We downloaded the hg19 coordinates of all Refseq annotated TSSs from the UCSC database, and created a region of [−1000,+1000] around each annotated gene, i.e. 1000 base pairs before the TSS and 1000 base pairs after the end of the gene. All regions that overlapped were then grouped into one cluster. If this cluster contained two or several non-overlapping regions, these were extracted, otherwise the region with most counts was chosen as cluster representative. Moreover, annotated TSSs with a gene shorter than 2000 bp were removed. After filtering, we were left with 13033 annotated TSSs. We took a region of [−2000,+2000] around those TSSs. After filtering away genes with no or very little DNaseIHS, 12757 genes were kept for CD4+ data, 12823 for IMR90 data, and all 13033 for H1 data (details in Text S1 Section 3).

The list of the 25 variables available (histone modifications and others) can be found in Text S1 Section 1.1. Unless specified, we use as variables the ones that are common to all cell types (23 variables, see column “used” in the table). Most histone modification data was downloaded from Zhao's group [6], [7] and from the Epigenomic Roadmap website [34]. The exact origin of all the data can be found in Text S1 Section 1.2.

#### Read counts and normalization

The data matrices were filled in by computing the levels of each variable around each gene in each cell type. For mRNA, the total number of RNA-seq reads found in the gene's body was computed and normalized by the spliced transcript's length, which was different for every gene. For all other variables, the total number of ChIP-seq reads found in the [−2000,+2000] region was computed and, for symmetry, normalized by the region's length (4000 base pairs).

#### Antibody cross-reactivity

Data for antibody cross-reactivity is not available, however some of these cross-reactions are reported in the literature. For each individual experiment, we looked up in [49] which antibody was used and we tracked potential cross-reactions. We used the information supplied in [50], [51] to build a table profiling the antibody's specificity for modifications of interest. The table and the procedure to obtain it are in Text S1 Section 2.

### Sparse partial correlation networks

#### Computation of the partial correlation matrices

In practice, for a dataset of interest, the inverse 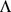 of the covariance matrix is computed. This matrix is then normalized row-wise and column-wise so that its diagonal is 1, and negated to obtain the PCM 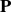. In other words 
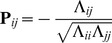
. The PCN is the graphical version of 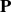, i.e. an edge is drawn between the variables 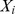 and 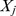 if and only if the entry 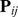 is significantly different from 0. The SPCN is a sparse version of 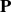, where entries (edges) are masked via the cross-validation scheme detailed in Section “Sparseness through cross-validation”.

#### Computation of the p-values and q-values

A 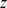-statistic is easily available for each entry in 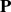 using Fisher's 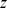-transform for correlation coefficients [52], where the degrees of freedom have been updated to take into account the number of control variables: 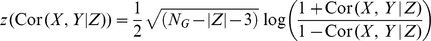. This 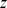-statistic follows approximately a canonical normal distribution (mean 0 and variance 1), from which p-values are straightforward to compute. If 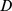 is the number of variables in the model, all of the 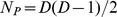 possible edges are tested, so the p-values are corrected for multiple testing using Benjamini-Hochberg's method. It has the effect of controlling the false discovery rate (FDR, the proportion of called positives that are real negatives) instead of the false positive rate (FPR, the proportion of real negatives that are called positives). Setting a threshold 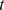 on the q-values now ensures an FDR of 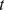.

#### Obtaining a sparse PCN

A few authors [53]–[55] have developed algorithms to optimize a regularized objective function, where the main term is the goodness-of-fit of a multivariate Gaussian with covariance 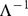, and the regularization term is a penalty on the number of entries in 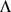. The optimal 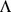 is then normalized to give the PCM 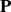. These algorithms follow the principle of LASSO for linear regression by using the L1-norm which imposes sparseness. These methods are very appealing however they also assume normality, and they are not designed to retrieve a real network, so they can change the structure if that helps improving the objective function. Indeed on our simulations, LASSO-type methods did not perform better than a simple threshold on the q-values, be it on numerical data or on rank data (see Text S1 Section 5.4). Through cross-validation, we obtain 10 sparse matrices, each with a different threshold. These 10 matrices are combined to produce a mask for the original PCM 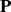.

#### The variable mRNA

As mentioned in introduction, the relationship of histone modifications to mRNA levels is of particular interest. Because a large region around the TSS had to be considered for computational purposes, we were afraid to lose interesting signals that perhaps happen in very localized regions (for example at the TSS) and not along the gene body, hence not giving a very high correlation compared to associations of histone modifications. To pick these associations up, if the mRNA node has fewer than 4 connections returned, the connections are completed (up to 4) using partial correlations of lower significance.

### Computation of the expected proportions and p-values for the overlap figures

With two lists of 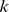 selected pairs from a pool of 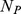 pairs, the number of common pairs follows a hypergeometric distribution with equal number of white balls and drawn balls (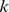) and with a total number of balls of 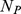, and a hypergeometric test is appropriate to compute p-values. The probability 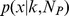 for 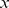 pairs to appear in the two lists is obtained through the hypergeometric distribution with 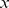 successes (white balls) in 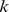 draws from a finite population of size 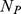 containing 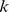 successes (white balls), so 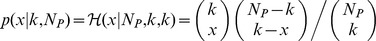. The expected number of same pairs in the two lists is therefore 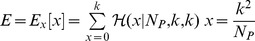, so the expected proportion is 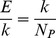, i.e. a straight line. The p-value is then given by the hypergeometric test: 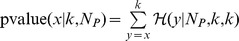. The appropriate call in R is 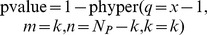.

With three lists, things are more complicated. The probability 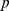 for a pair to appear in the three lists is obtained through a Binomial distribution with number of trials 3 and probability 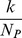, so 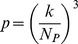. The expected number of pairs common to the three lists is 
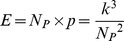
, the expected proportion is therefore 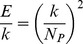, i.e. a quadratic curve. For an observation 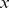, the p-value is computed by simulating 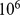 intersections between three lists containing 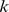 pairs sampled randomly from 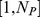 with replacement, and by counting the proportion of times the length of these intersections was at least as high as 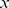. If the result is 0, 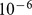 is reported as upper bound.

## Supporting Information

Text S1 **Supporting information for the manuscript.** The PDF file TextS1.pdf contains 10 sections, 13 tables and 68 figures.
(PDF)
