# Supplementary material for: Finding Associations among Histone Modifications Using Sparse Partial Correlation Networks
Source: PLoS Comput Biol. 2013 Sep 5;9(9):e1003168. doi: 10.1371/journal.pcbi.1003168 (PMC3764007; doi:10.1371/journal.pcbi.1003168)
Supplement: Text S1 — Supporting information for the manuscript. The PDF file TextS1.pdf contains 10 sections, 13 tables and 68 figures. (PDF) [file pcbi.1003168.s001.pdf]

Finding associations among histone modifications using sparse partial correlation networks

Text S1

Contents

1 The data **2**
- 1.1 Variables downloaded . . . 2
- 1.2 Accessions and references . . . 3
- 1.3 ENCODE data . . . 6

2 Antibodies' specificity **8**

3 Refseq genes and data extraction **10**

4 Variables' density **11**

5 The method **12**
- 5.1 Distributions of z-statistics . . . 12
- 5.2 Distributions of p-values . . . 14
- 5.3 Use of rank data instead of numerical data . . . 16
  - 5.3.1 Simulations . . . 16
  - 5.3.2 CD4+ data . . . 24
- 5.4 PCNs' sparseness: p-value threshold against GLASSO . . . 25
- 5.5 Use of rank data instead of discrete data . . . 29

6 Controls **32**
- 6.1 ChIP-seq controls using data common to Zhao, Roadmap and ENCODE . . . 32
- 6.2 ChIP-seq controls using data common to Zhao and Roadmap . . . 33

7 The three networks **34**
- 7.1 Network for CD4+ cells . . . 34
- 7.2 Network for IMR90 cells . . . 36
- 7.3 Network for H1 cells . . . 38

8 Effect matrices **40**
- 8.1 Effect matrix for CD4+ cells . . . 40
- 8.2 Effect matrix for IMR90 cells . . . 42
- 8.3 Effect matrix for H1 cells . . . 44
- 8.4 Consenus effect matrix . . . 45

9 Scatter plots of the residuals **47**
- 9.1 H3K36me3 against mRNA . . . 48
- 9.2 H3K9me3 against H3K27me3 . . . 50
- 9.3 H3K79me2 against H3K4me1 . . . 52
- 9.4 H3K4me3 against H3K27me3 . . . 54
- 9.5 H4K5ac against H4K20me1 . . . 56

10 Explaining away pairs of histone modifications **58**

References **70**

1 The data

1.1 Variables downloaded

	CD4+	IMR90	H1	used (unless otherwise specified)
RNA-seq	✓	✓	✓	✓
Input		✓	✓	in Supplementary Material Section 6
DNaseIHS	✓	✓	✓	✓
MNase	✓			in Supplementary Material Section 6
H2AK5ac	✓	✓	✓	✓
H2BK120ac	✓	✓	✓	✓
H2BK12ac	✓	✓	✓	✓
H2BK20ac	✓	✓	✓	✓
H3K14ac	✓	✓	✓	✓
H3K18ac	✓	✓	✓	✓
H3K23ac	✓	✓	✓	✓
H3K27ac	✓	✓	✓	✓
H3K27me3	✓	✓	✓	✓
H3K36me3	✓	✓	✓	✓
H3K4ac	✓	✓	✓	✓
H3K4me1	✓	✓	✓	✓
H3K4me2	✓	✓	✓	✓
H3K4me3	✓	✓	✓	✓
H3K79me1	✓	✓	✓	✓
H3K79me2	✓	✓	✓	✓
H3K9ac	✓	✓	✓	✓
H3K9me3	✓	✓	✓	✓
H4K20me1	✓	✓	✓	✓
H4K5ac	✓	✓	✓	✓
H4K91ac	✓	✓	✓	✓

Table S1: **List of the 25 variables.** Each column shows the variables that were downloaded for a particular cell type. The 23 variables that are actually used to build all the networks presented throughout the article are indicated in the last column. If a network uses additional or fewer variables, it is explicitly specified.

1.2 Accessions and references

cell type	experiment	antibody	reference	accession
CD4+	RNA-seq		[1]	SRX005317
CD4+	DNaseIHS-seq		[2]	SRX040388, SRX040415
CD4+	MNase-seq		[3]	SRX000168
CD4+	H2AK5ac-ChIP-seq	Abcam ab1764	[4]	SRX000354
CD4+	H2BK120ac-ChIP-seq	Upstate 07-564	[5]	SRX000356
CD4+	H2BK12ac-ChIP-seq	Abcam ab40883	[5]	SRX000357
CD4+	H2BK20ac-ChIP-seq	Upstate 07-347	[5]	SRX000358
CD4+	H3K14ac-ChIP-seq	Upstate 07-353	[4]	SRX000360
CD4+	H3K18ac-ChIP-seq	Abcam ab1191	[4]	SRX000361
CD4+	H3K23ac-ChIP-seq	Upstate 07-355	[4]	SRX000362
CD4+	H3K27ac-ChIP-seq	Abcam ab4729	[4]	SRX000363
CD4+	H3K27me3-ChIP-seq	Upstate 07-449	[5]	SRX000143, SRX000364
CD4+	H3K36me3-ChIP-seq	Abcam ab9050	[5]	SRX000145
CD4+	H3K4ac-ChIP-seq	Upstate 07-539	[5]	SRX000366
CD4+	H3K4me1-ChIP-seq	Abcam ab8895	[5]	SRX000146
CD4+	H3K4me2-ChIP-seq	Abcam ab7766	[5]	SRX000147
CD4+	H3K4me3-ChIP-seq	Abcam ab8580	[5]	SRX000148
CD4+	H3K79me1-ChIP-seq	Abcam ab2886	[5]	SRX000149, SRX000367
CD4+	H3K79me2-ChIP-seq	Abcam ab3594	[5]	SRX000150, SRX000368
CD4+	H3K9ac-ChIP-seq	Abcam ab4441	[4]	SRX000370
CD4+	H3K9me3-ChIP-seq	Abcam ab8898	[5]	SRX000154
CD4+	H4K20me1-ChIP-seq	Aabcam ab9051	[5]	SRX000157
CD4+	H4K5ac-ChIP-seq	Upstate 07-327	[4]	SRX000373
CD4+	H4K91ac-ChIP-seq	Abcam ab4627	[4]	SRX000375

Table S2: **List of experiments and antibodies for CD4+ data.** For each variable at hand, we give the various antibodies that were used if appropriate, the reference article, and the experiments' identifiers.

cell type	experiment	antibody	reference	accession
IMR90	RNA-seq		[2]	SRX007167
IMR90	Input-seq		[2]	SRX017545, SRX017546, SRX017547, SRX017548, SRX017549, SRX017550, SRX017551, SRX017552
IMR90	DNaseIHS-seq		[2]	SRX012425, SRX012426, SRX018822, SRX018823
IMR90	H2AK5ac-ChIP-seq	Abcam ab45152	[2]	SRX017485, SRX017487
IMR90	H2BK120ac-ChIP-seq	Upstate 07-567	[2]	SRX017488, SRX017489
IMR90	H2BK12ac-ChIP-seq	Abcam ab40883	[2]	SRX017490, SRX017492, SRX017493
IMR90	H2BK20ac-ChIP-seq	Upstate 07-347	[2]	SRX017498, SRX017499
IMR90	H3K14ac-ChIP-seq	Upstate 07-353	[2]	SRX017500, SRX017502
IMR90	H3K18ac-ChIP-seq	Abcam ab1191	[2]	SRX012495, SRX017503
IMR90	H3K23ac-ChIP-seq	Upstate 07-355	[2]	SRX017504, SRX017505
IMR90	H3K27ac-ChIP-seq	Abcam ab4729	[2]	SRX012496, SRX012497, SRX017506
IMR90	H3K27me3-ChIP-seq	Upstate 07-449	[2]	SRX012498, SRX017508
IMR90	H3K36me3-ChIP-seq	Abcam ab9050	[2]	SRX017509, SRX017511
IMR90	H3K4ac-ChIP-seq	Upstate 07-539	[2]	SRX017512, SRX017513
IMR90	H3K4me1-ChIP-seq	Abcam ab4729	[2]	SRX017514, SRX017516, SRX017517
IMR90	H3K4me2-ChIP-seq	Abcam ab32356	[2]	SRX017518, SRX017519
IMR90	H3K4me3-ChIP-seq	ab8580 / AM39159	[2]	SRX012500, SRX017520
IMR90	H3K79me1-ChIP-seq	Abcam ab2886	[2]	SRX017523, SRX017525, SRX017526, SRX017527
IMR90	H3K79me2-ChIP-seq	Abcam ab3594	[2]	SRX017528, SRX017530
IMR90	H3K9ac-ChIP-seq	ab4441 / AM39137	[2]	SRX012503, SRX017531
IMR90	H3K9me3-ChIP-seq	Abcam ab8898	[2]	SRX012504, SRX017532, SRX017533
IMR90	H4K20me1-ChIP-seq	Abcam ab9051	[2]	SRX017534, SRX017536
IMR90	H4K5ac-ChIP-seq	Upstate 07-327	[2]	SRX012505, SRX017537
IMR90	H4K91ac-ChIP-seq	Abcam ab4627	[2]	SRX017543, SRX017544

Table S3: **List of experiments and antibodies for IMR90 data.** For each variable at hand, we give the various antibodies that were used if appropriate, the reference article, and the experiments' identifiers.

cell type	experiment	antibody	reference	accession
H1	RNA-seq		[6]	SRX026667, SRX026669, SRX026674, SRX026685, SRX026694
H1	Input-seq		[2]	SRX006268, SRX010893, SRX027882, SRX027883, SRX027884, SRX027885, SRX027886, SRX027887, SRX027888, SRX040606, SRX056744
H1	DNaseIHS-seq		[6]	SRX031224
H1	H2AK5ac-ChIP-seq	Abcam ab45152	[2]	SRX027691, SRX027889
H1	H2BK120ac-ChIP-seq	Active Motif AM39119	[2]	SRX027844
H1	H2BK12ac-ChIP-seq	Abcam ab40883	[2]	SRX027845, SRX027846
H1	H2BK20ac-ChIP-seq	Upstate 07-347	[2]	SRX027849, SRX027850
H1	H3K14ac-ChIP-seq	Abcam ab52946	[2]	SRX056726, SRX056727
H1	H3K18ac-ChIP-seq	Abcam ab1191	[2]	SRX027692, SRX027853
H1	H3K23ac-ChIP-seq	Upstate 07-355	[2]	SRX056729, SRX056730
H1	H3K27ac-ChIP-seq	ab4729 / AM39133	[2]	SRX012366, SRX038526, SRX056722
H1	H3K27me3-ChIP-seq	CST9733 / U07-449 / M07-449	[2]	SRX006262, SRX006874, SRX007379, SRX012368, SRX027857, SRX038527, SRX038528
H1	H3K36me3-ChIP-seq	Abcam ab9050	[2]	SRX006235, SRX006270, SRX007388, SRX010891, SRX012371, SRX027858, SRX038529, SRX038530
H1	H3K4ac-ChIP-seq	Upstate 07-539	[2]	SRX027860, SRX056734
H1	H3K4me1-ChIP-seq	ab8895 / U07-436	[2]	SRX006236, SRX006873, SRX007389, SRX012373, SRX027861, SRX038531, SRX038532
H1	H3K4me2-ChIP-seq	ab32356 / ab7766	[2]	SRX027693, SRX027694, SRX038533, SRX038534
H1	H3K4me3-ChIP-seq	CST9751 / M04-745 / ab8580 / M04-473	[2]	SRX006117, SRX006237, SRX006798, SRX007385, SRX012501, SRX027298, SRX027864, SRX038535, SRX038536
H1	H3K79me1-ChIP-seq	Abcam ab2886	[2]	SRX027867, SRX027868, SRX027869
H1	H3K79me2-ChIP-seq	Abcam ab3594	[2]	SRX027870, SRX027871
H1	H3K9ac-ChIP-seq	M07-352 / ab4441 / AM39137	[2]	SRX006116, SRX006875, SRX007386, SRX027872, SRX038537, SRX038538
H1	H3K9me3-ChIP-seq	Abcam ab8898	[2]	SRX006263, SRX007387, SRX010889, SRX027874, SRX027876, SRX027877
H1	H4K20me1-ChIP-seq	Abcam ab9051	[2]	SRX027878, SRX038539, SRX038540
H1	H4K5ac-ChIP-seq	Upstate 07-327	[2]	SRX027879
H1	H4K91ac-ChIP-seq	Abcam ab4627	[2]	SRX027881

Table S4: **List of experiments and antibodies for H1 data.** For each variable at hand, we give the various antibodies that were used if appropriate, the reference article, and the experiments' identifiers.

1.3 ENCODE data

The Zhao/Roadmap data described above is the data used in most of Main Document. However, in Main Document Section "Stability across cell types", data from the ENCODE project [7] is used in addition to the Zhao/Roadmap data. ENCODE data has fewer histone modifications available, so we created a smaller subset to work with for this one particular section of Main Document. This temporary dataset is described below.

	CD4+	IMR90	H1 (Roadmap)	H1 (ENCODE)	used (unless otherwise specified)
RNA-seq	✓	✓	✓	✓	✓
Input		✓	✓	✓	in Supplementary Material Section 6
DNaseIHS	✓	✓	✓	✓	✓
MNase	✓				in Supplementary Material Section 6
H2AK5ac	✓	✓	✓		
H2BK120ac	✓	✓	✓		
H2BK12ac	✓	✓	✓		
H2BK20ac	✓	✓	✓		
H3K14ac	✓	✓	✓		
H3K18ac	✓	✓	✓		
H3K23ac	✓	✓	✓		
H3K27ac	✓	✓	✓	✓	✓
H3K27me3	✓	✓	✓	✓	✓
H3K36me3	✓	✓	✓	✓	✓
H3K4ac	✓	✓	✓		
H3K4me1	✓	✓	✓	✓	✓
H3K4me2	✓	✓	✓	✓	✓
H3K4me3	✓	✓	✓	✓	✓
H3K79me1	✓	✓	✓		
H3K79me2	✓	✓	✓	✓	✓
H3K9ac	✓	✓	✓	✓	✓
H3K9me3	✓	✓	✓	✓	✓
H4K20me1	✓	✓	✓	✓	✓
H4K5ac	✓	✓	✓		
H4K91ac	✓	✓	✓		

Table S5: **List of the 25 variables.** Each column shows the variables that were downloaded for a particular cell type. The 12 variables that are actually used to build all the networks in Main Document Section "Stability across cell types" are indicated in the last column.

The ENCODE data was downloaded from the website http://encodeproject.org/ENCODE/downloads.html. The exact addresses are given below:

cell type	experiment	replicates used	address of the first replicate
H1	RNA-seq	Rep1 - Rep4	wgEncodeCaltechRnaSeq/wgEncodeCaltechRnaSeqH1hescR2x75Il200AlignsRep1V2.bam
H1	Input-seq	Rep1 - Rep2	wgEncodeBroadHistone/wgEncodeBroadHistoneH1hescControlStdAlnRep1.bam
H1	DNaseIHS-seq	Rep1 - Rep2	wgEncodeOpenChromDnase/wgEncodeOpenChromDnaseH1hescAlnRep1.bam
H1	H3K27ac-ChIP-seq	Rep1 - Rep2	wgEncodeBroadHistone/wgEncodeBroadHistoneH1hescH3k27acStdAlnRep1.bam
H1	H3K27me3-ChIP-seq	Rep1 - Rep2	wgEncodeBroadHistone/wgEncodeBroadHistoneH1hescH3k27me3StdAlnRep1.bam
H1	H3K36me3-ChIP-seq	Rep1 - Rep2	wgEncodeBroadHistone/wgEncodeBroadHistoneH1hescH3k36me3StdAlnRep1.bam
H1	H3K4me1-ChIP-seq	Rep1 - Rep2	wgEncodeBroadHistone/wgEncodeBroadHistoneH1hescH3k4me1StdAlnRep1.bam
H1	H3K4me2-ChIP-seq	Rep1 - Rep2	wgEncodeBroadHistone/wgEncodeBroadHistoneH1hescH3k4me2StdAlnRep1.bam
H1	H3K4me3-ChIP-seq	Rep1 - Rep2	wgEncodeBroadHistone/wgEncodeBroadHistoneH1hescH3k4me3StdAlnRep1.bam
H1	H3K79me2-ChIP-seq	Rep1 - Rep2	wgEncodeBroadHistone/wgEncodeBroadHistoneH1hescH3k79me2StdAlnRep1.bam
H1	H3K9ac-ChIP-seq	Rep1 - Rep2	wgEncodeBroadHistone/wgEncodeBroadHistoneH1hescH3k9acStdAlnRep1.bam
H1	H3K9me3-ChIP-seq	Rep1 - Rep2	wgEncodeBroadHistone/wgEncodeBroadHistoneH1hescH3k09me3StdAlnRep1.bam
H1	H4K20me1-ChIP-seq	Rep1 - Rep2	wgEncodeBroadHistone/wgEncodeBroadHistoneH1hescH4k20me1StdAlnRep1.bam

Table S6: **List of ENCODE experiments for H1 cells.** For each variable at hand, we give the address of the bam files that was used (the prefix is http://hgdownload.cse.ucsc.edu/goldenPath/hg19/encodeDCC/). When several replicates are mentioned, the several corresponding bam files were merged into a single bam file.

2 Antibodies' specificity

For each individual experiment, we looked up in [8] which antibody was used and we tracked potential cross-reactions. We used the information supplied in [9, 10] to build a table profiling the antibody's specificity for modifications of interest. The results are given in Fig. S1. Rows represent antibodies, columns histone modifications. Cross-reactions are marked in dark red. For some antibodies, information is missing, so the row was left empty, even for the histone mark the antibody is designed for.

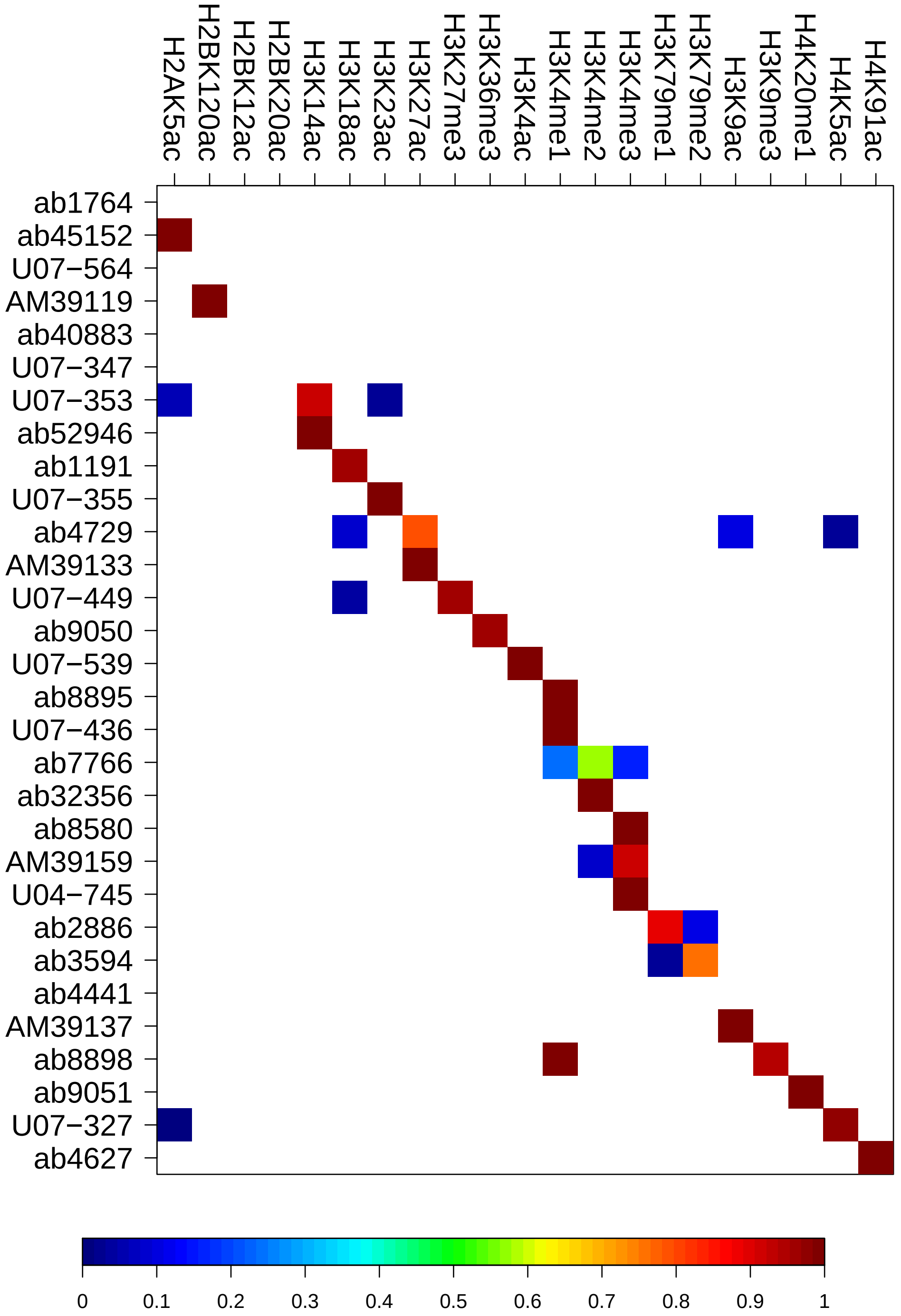

Figure S1: **Antibodies' specificity.** Each row is an antibody, each column is a histone modification of interest. If an antibody is known to react with a histone mark, the appropriate cell in the matrix is colored. The color itself represents the strength of the cross-reaction according to the color code underneath.

We wanted to have an idea of which edges could potentially be explained by antibodies' cross-reactivity. We created a square matrix representing possible pairs of histone modifications. For each pair of histone modifications, and for each cell type, we checked whether one of the antibodies used in the relevant experiments cross-reacted with both modifications in the pair. If such an antibody existed, the pair was listed for the relevant cell type. If a pair was listed for at least two cell types, it was marked in dark red in the matrix. The results are given in Fig. S2.

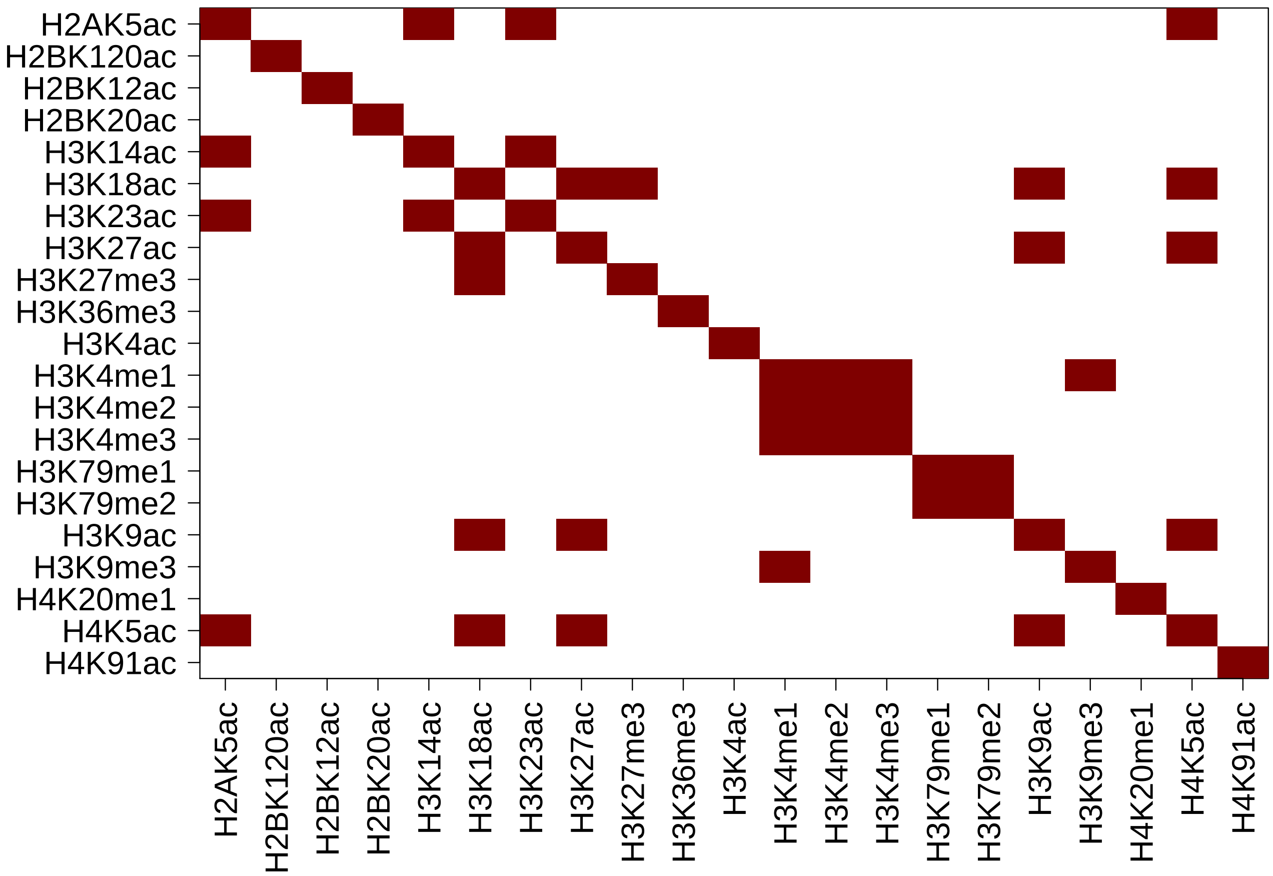

Figure S2: **Edges possibly explained by antibodies' cross-reactivity.** A cell is marked in red if an antibody cross-reaction between the 2 relevant histone modifications has been observed for at least 2 cell types.

3 Refseq genes and data extraction

We downloaded the hg19 coordinates of all Refseq annotated TSSs from the UCSC database, and created a region of [-1000,+1000] around each annotated gene, i.e. 1000 base pairs before the TSS and 1000 base pairs after the end of the gene. All regions that overlapped were then grouped into one cluster. If this cluster contained two or several non-overlapping regions, these were extracted, otherwise the region with most counts was chosen as cluster representative. Moreover, annotated TSSs with a gene shorter than 2000bp were removed. After filtering, we were left with 13033 annotated TSSs. We took a region of [-2000,+2000] around those TSSs.

For mRNA, the total number of reads *reads* found in the gene's body was computed and normalized by the spliced transcript's length, which was different for every gene, giving $\frac{reads}{\text{length of the transcript}}$. For all other markers, the total number of reads *reads* found in the [-2000,+2000] region was computed and, for symmetry, normalized by the region's length (4000 base pairs), giving $\frac{reads}{4000}$.

When visualization is required, as in Supplementary Material Section 4, mRNA levels are computed using $\log\left(\frac{reads+1}{\text{length of the transcript}}\right)$, and other levels using $\log\left(\frac{reads+1}{4000}\right)$. The log data is then standardized to mean 0 and variance 1.

Genes with DNaseIHS log levels lower than -7 were discarded, both in the CD4+ dataset and in the IMR90 dataset. Therefore 12757 genes were kept for CD4+ data, 12823 for IMR90 data, and all 13033 for H1 data.

The exon content of the genes used in the study is given below in Fig. S3. The proportion of exonic DNA is

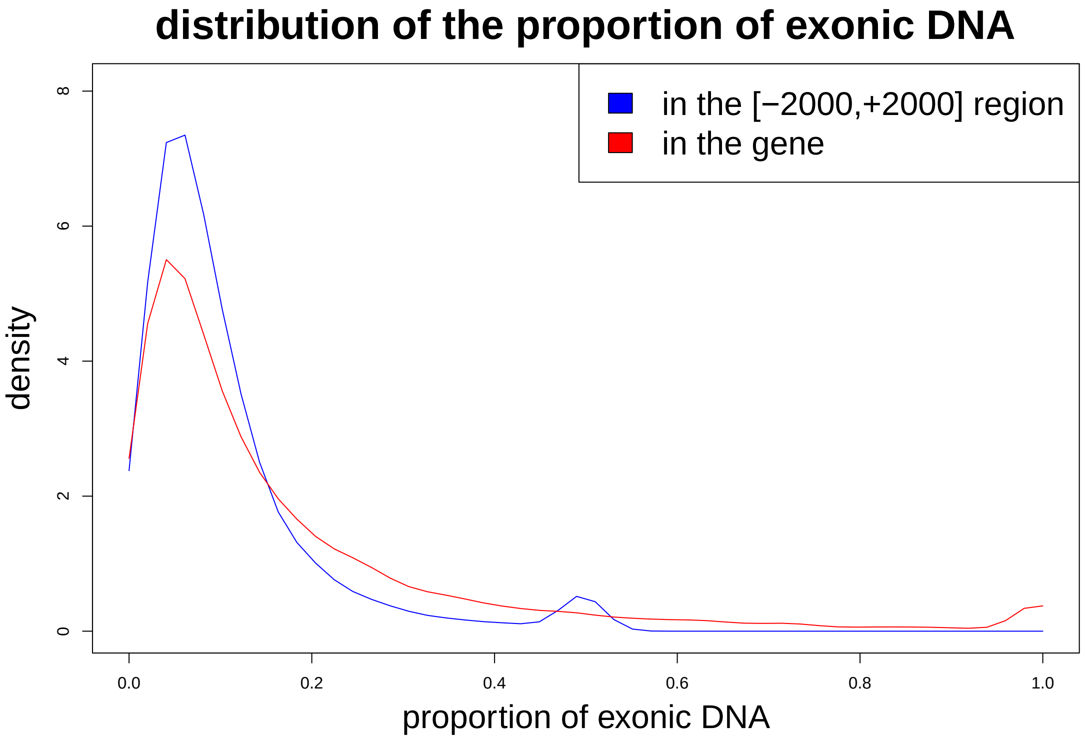

Figure S3: **Distribution of the proportion of exonic DNA in the genes.**

expectedly low. Most regions contain less than 40% exonic DNA, with a peak around 5%.

4 Variables' density

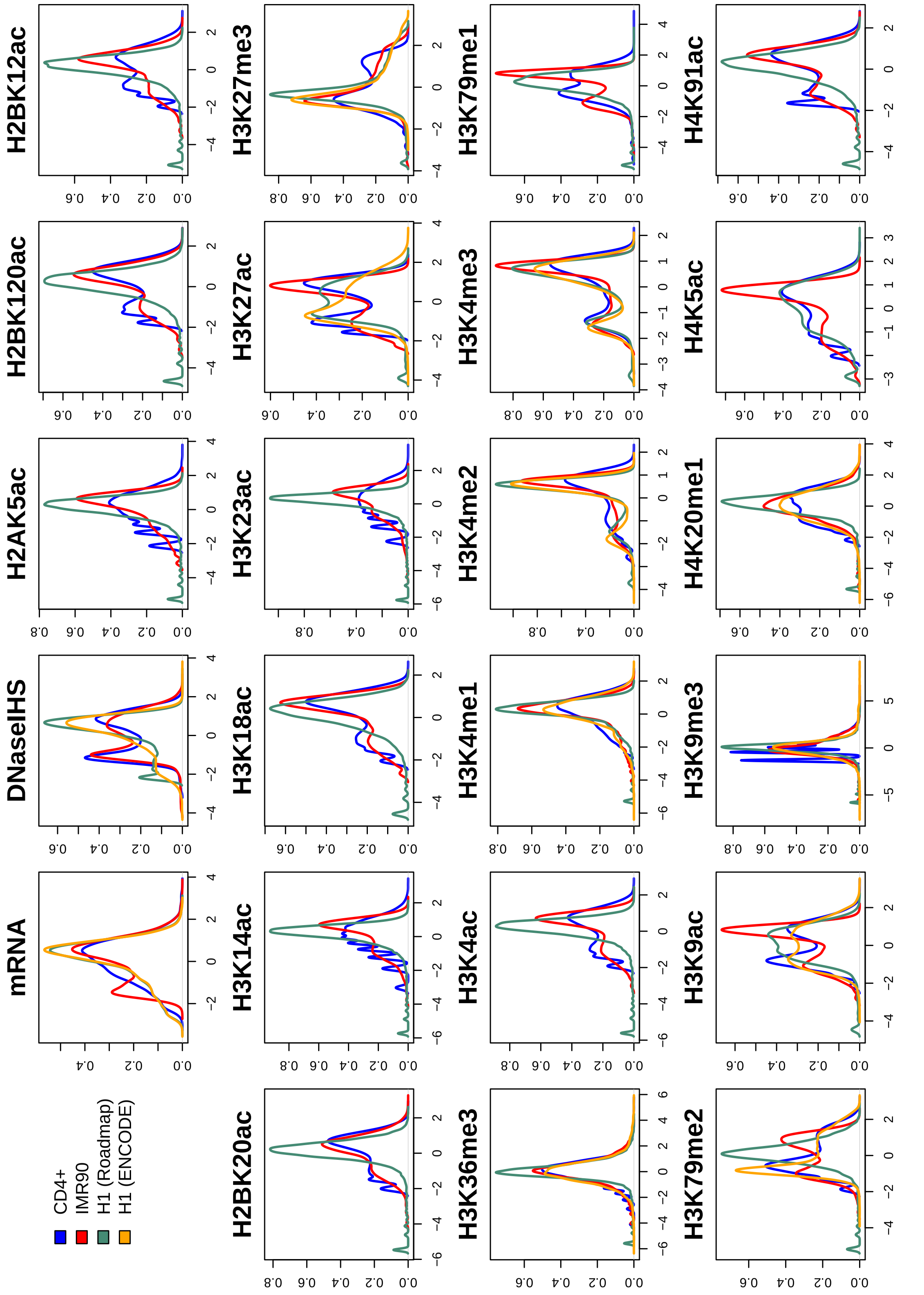

Figure S4: **The variables' density. Blue:** in CD4+ cells. **Red:** in IMR90 cells. **Green:** in H1 (Roadmap) cells. **Orange:** in H1 (ENCODE) cells.

5 The method

5.1 Distributions of z-statistics

As described in Main Document Section "Materials and Methods", the z-statistic that is used throughout this study is Fischer's z-transform [11]:

$$z(\mathrm{Cor}(X,Y|Z)) = \frac{1}{2}\sqrt{(N_G - |Z| - 3)}\log\left(\frac{1+\mathrm{Cor}(X,Y|Z)}{1-\mathrm{Cor}(X,Y|Z)}\right)$$

where N_G is the sample size and $|Z|$ denotes the number of control variables. It is the z-transform used for correlation coefficients, but the degrees of freedom $(N_G - 3)$ are now corrected for the number of control variables.

Figure S5 shows, under the form of histograms, and for each cell type and each gene type separately, the distribution of the z-statistics obtained using Fischer's z-transform on the partial correlation coefficients in the relevant PCM. Remember that the partial correlation coefficients are computed on rank data. There are 23 variables, so there are $(23 \times 22/2 = 253)$ independent z-statistics, and each histogram is built on these 253 z-statistics. The distribution expected by chance (canonical normal distribution) is shown in red.

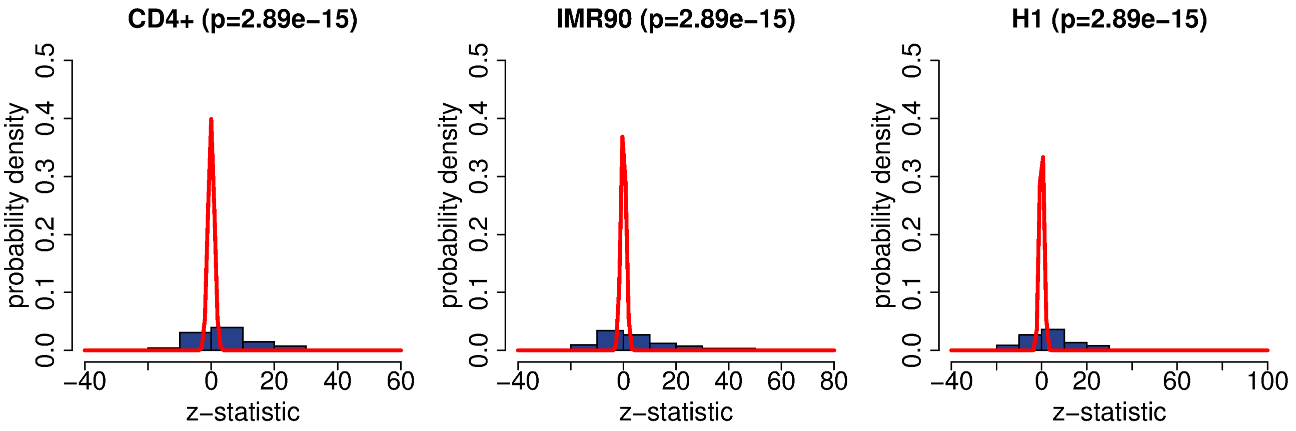

Figure S5: **Distributions of z-statistics.** The distribution expected by chance (canonical normal) is shown in red, and the p-values for the data being sampled from this distribution according to Kolmogorov-Smirnov test are given in brackets. **Left:** CD4+ cells. **Center:** IMR90 cells. **Right:** H1 cells.

In order to assess the validity of these z-statistics on rank data, the data was randomised for each cell type and each gene type separately. Randomised means here that the rows of the data matrix (i.e. the samples) were randomly permuted. The PCM was then computed on this randomised data. Figure S6 shows the same distributions as in Fig. S5, but after randomisation. The observed histograms do not vary much from the expected ones, which indicates that the z-statistics behave well.

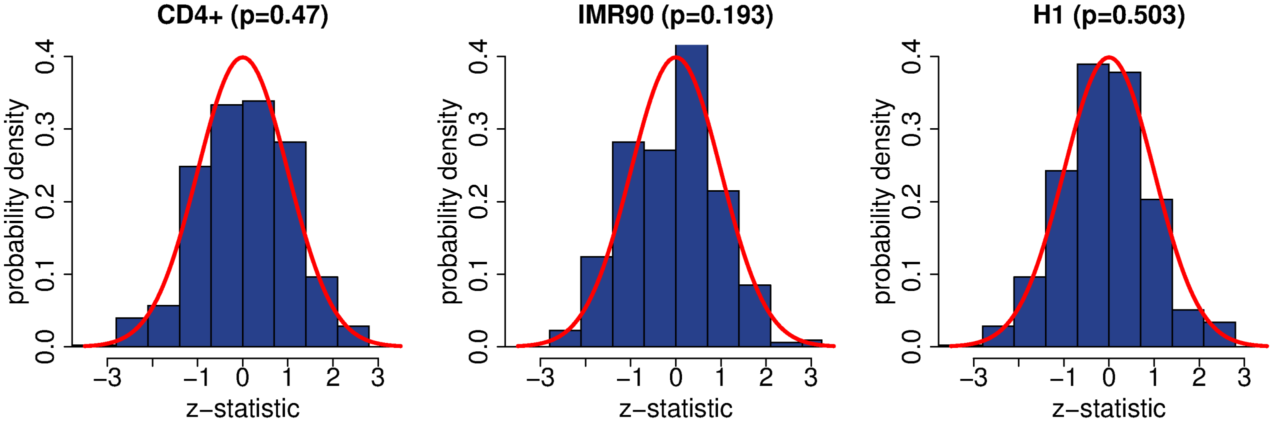

Figure S6: **Distributions of z-statistics for randomised data.** The distribution expected by chance (canonical normal) is shown in red, and the p-values for the data being sampled from this distribution according to Kolmogorov-Smirnov test are given in brackets. **Left:** CD4+ cells. **Center:** IMR90 cells. **Right:** H1 cells.

For smoothness, the randomisation process was repeated 20 times with different permutations, and the 20 resulting sets of z-statistics were concatenated, so as to obtain in total $20 \times 253 = 5060$ z-statistics. The new distributions are shown in Fig. S7. The observed histograms vary now very little from the expected ones, demonstrating the good behaviour of the z-statistics.

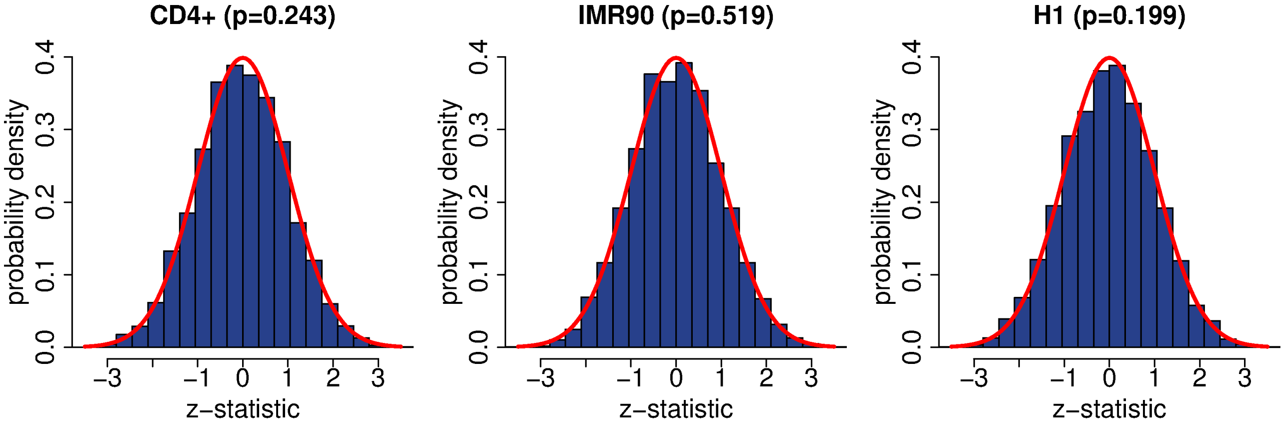

Figure S7: **Distributions of z-statistics for 20 randomisations of the data.** The distribution expected by chance (canonical normal) is shown in red, and the p-values for the data being sampled from this distribution according to Kolmogorov-Smirnov test are given in brackets. **Left:** CD4+ cells. **Center:** IMR90 cells. **Right:** H1 cells.

5.2 Distributions of p-values

Figure S8 shows, under the form of histograms, and for each cell type and each gene type separately, the distributions of the p-values obtained using Fischer's z-transform on the partial correlation coefficients in the relevant PCM. Remember that the partial correlation coefficients are computed on rank data. There are 23 variables, so there are $(23 \times 22/2 = 253)$ independent p-values, and each histogram is built on these 253 p-values. The distribution expected by chance (uniform distribution over [0,1]) is shown in red.

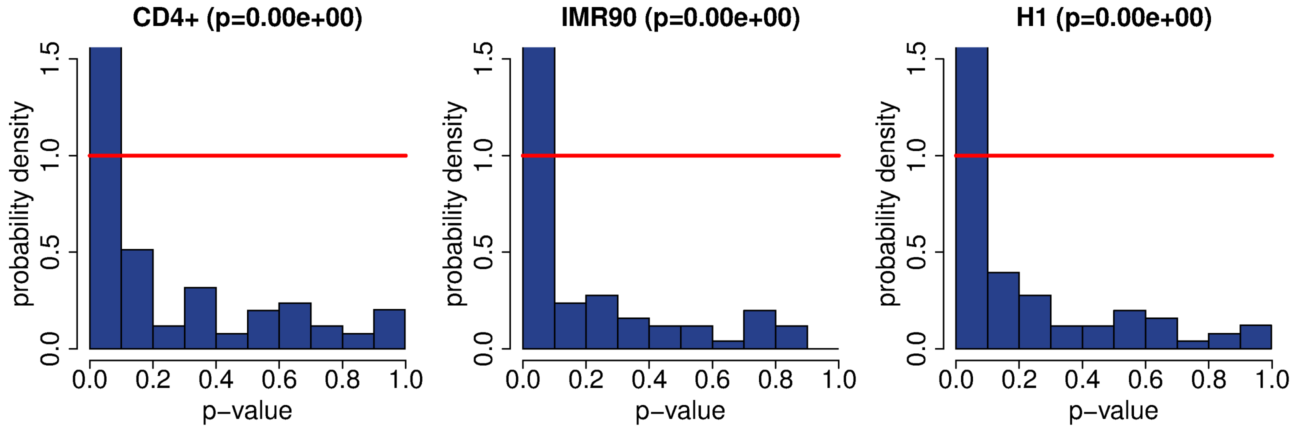

Figure S8: **Distributions of p-values.** The distribution expected by chance (uniform over [0,1]) is shown in red, and the p-values for the data being sampled from this distribution according to Kolmogorov-Smirnov test are given in brackets. **Left:** CD4+ cells. **Center:** IMR90 cells. **Right:** H1 cells.

In order to assess the validity of these p-values on rank data, the data was randomised for each cell type and each gene type separately. Randomised means here that the rows of the data matrix (i.e. the samples) were randomly permuted. The PCM was then computed on this randomised data. Figure S9 shows the same distributions as in Fig. S8, but after randomisation. The observed histograms do not vary much from the expected ones, which indicates that the p-values behave well.

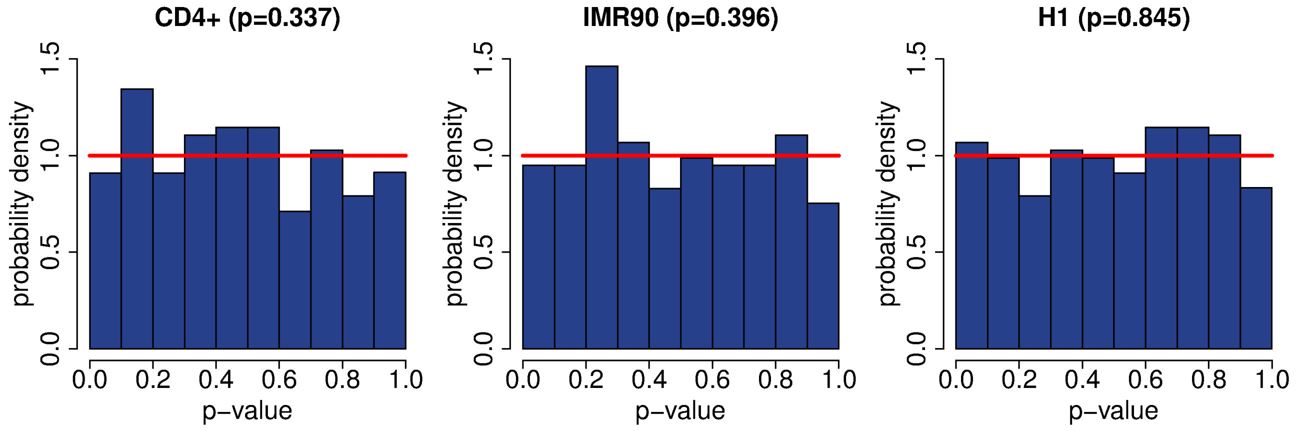

Figure S9: **Distributions of p-values for randomised data.** The distribution expected by chance (uniform over [0,1]) is shown in red, and the p-values for the data being sampled from this distribution according to Kolmogorov-Smirnov test are given in brackets. **Left:** CD4+ cells. **Center:** IMR90 cells. **Right:** H1 cells.

For smoothness, the randomisation process was repeated 20 times with different permutations and the 20 resulting sets of p-values were concatenated, so as to obtain in total $20 \times 253 = 5060$ p-values. The new distributions are shown in Fig. S10. The observed histograms vary now very little from the expected ones, demonstrating the good behaviour of the p-values.

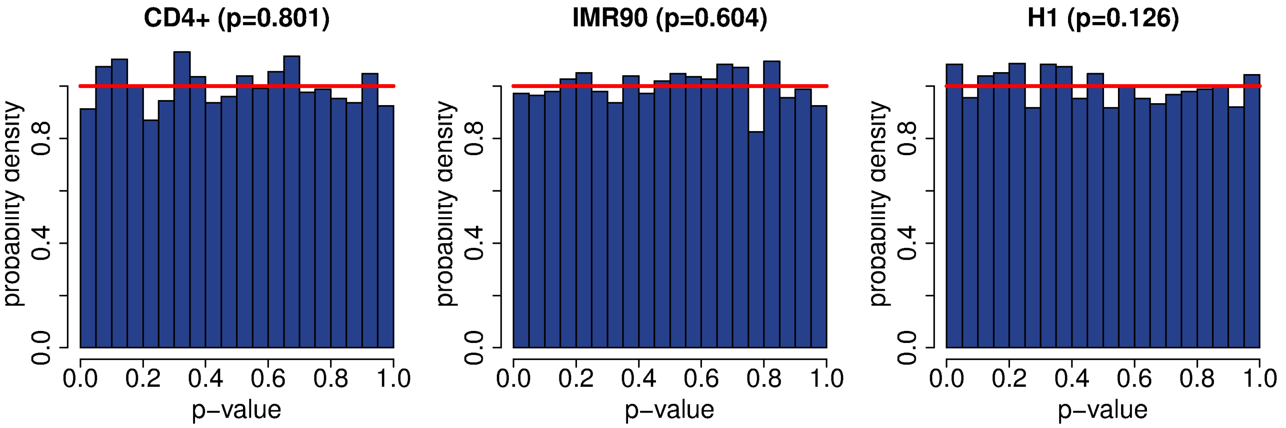

Figure S10: **Distributions of p-values for 20 randomisations of the data.** The distribution expected by chance (uniform over [0,1]) is shown in red, and the p-values for the data being sampled from this distribution according to Kolmogorov-Smirnov test are given in brackets. **Left:** CD4+ cells. **Center:** IMR90 cells. **Right:** H1 cells.

5.3 Use of rank data instead of numerical data

5.3.1 Simulations

In order to be robust to distribution changes (distributions do not always look the same from experiment to experiment, and are often not Gaussian), the data was ranked prior to analysis. To assess whether this was an inspired choice, we performed several simulation studies, two of which are reported below.

1st simulation

A Bayesian network was designed as in Fig. S11 (left), and its corresponding undirected network constructed as in Fig. S11 (right).

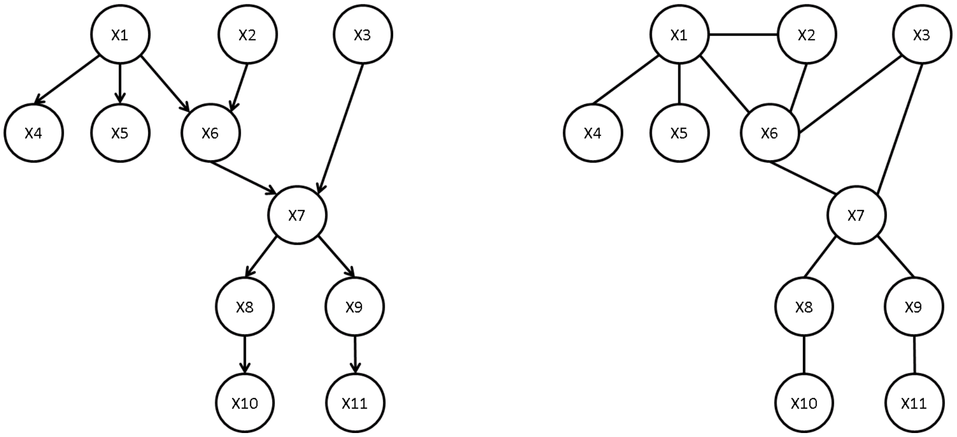

Figure S11: **Simulated networks. Left:** original Bayesian network used to generate data. **Right:** the corresponding undirected network the methods should find.

The Bayesian network was used to generate 5000 datapoints, according to the following distributions:

$$\begin{aligned}
\mu &\sim \mathcal{N}(0, I_D) \\
\log\left(\sigma^2\right) &\sim \mathcal{N}(0, I_D) \\
\\
X_1 &\sim \mathcal{N}(\mu_1 \ , \ \sigma_1) \\
X_2 &\sim \mathcal{N}(\mu_2 \ , \ \sigma_2) \\
X_3 &\sim 0.6\,\mathcal{N}(\mu_3 - 10 \ , \ \sigma_3) + 0.4\,\mathcal{N}(\mu_3 + 10 \ , \ 0.5\ \sigma_1) \\
X_4 &\sim 0.7\,\mathcal{N}(\mu_4\ X_1 \ , \ \sigma_4) + 0.3\,\mathcal{N}(0 \ , \ 1) \\
X_5 &\sim \mathcal{N}(\mu_5 + X_1 \ , \ \sigma_5) \\
X_6 &\sim \mathcal{G}\left(\text{shape} = 1 + \frac{\mu_6 + 2X_1 + X_2 + 20}{5\ \sigma_6^2} \ , \ \text{scale} = 5\ \sigma_6^2\right) \\
X_7 &\sim 0.7\,\mathcal{N}(X_3 + X_6 + \mu_7 \ , \ \sigma_7) + 0.3\,\mathcal{N}(\mu_7 - 2X_3 + 3X_6 \ , \ \sigma_7) \\
X_8 &\sim \mathcal{N}(\mu_8 + 0.1X_7 \ , \ \sigma_8) \\
X_9 &\sim \mathcal{N}(\mu_9 + 0.1X_7 \ , \ \sigma_9) \\
X_{10} &\sim \mathcal{N}(\mu_{10} + X_8 \ , \ \sigma_{10}) \\
X_{11} &\sim \mathcal{N}(\mu_{11} + X_9 \ , \ \sigma_{11})
\end{aligned}$$

which led to the variables shown in Fig. S12.

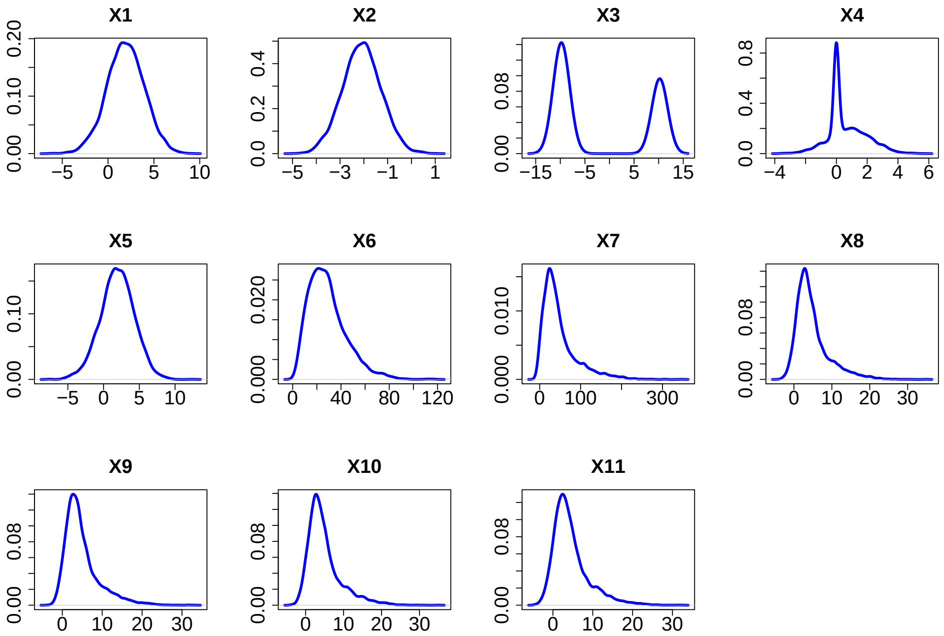

Figure S12: **Distributions of the toy variables.** On the x-axis are the values that can be taken by the variable under study, and on the y-axis the probability density.

The pairwise relationships between the variables are plotted in Fig. S13.

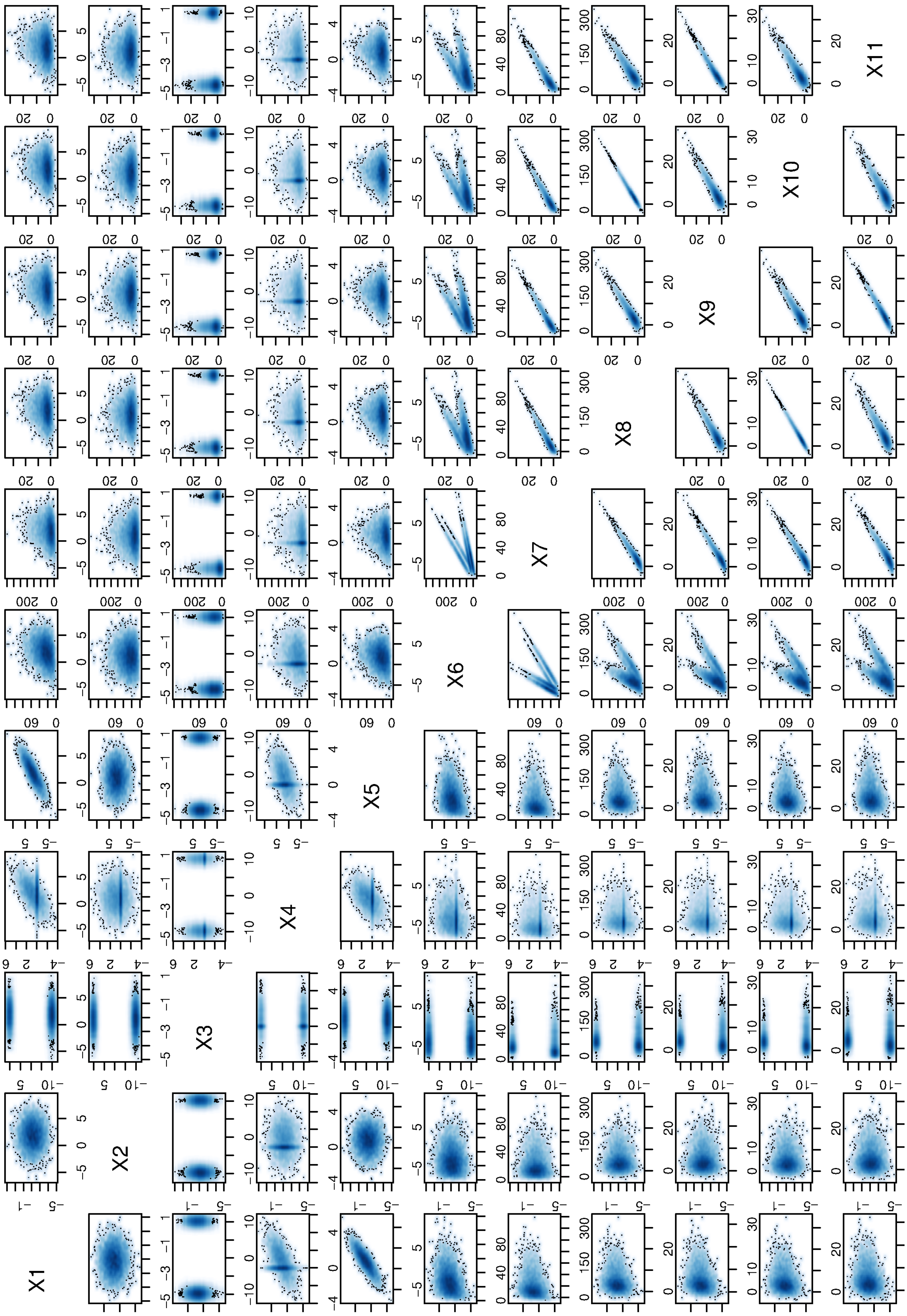

Figure S13: **Pairwise relationships between the toy variables.** The cell $M(i, j)$ in this matrix shows the scatter plot of variable X_i against variable X_j.

The PCM, its associated p-values and the precision matrix Λ (the inverse of the covariance matrix) were computed (the latter is an un-normalised version of the PCM). The p-values were ranked and the corresponding edges removed one at a time, in decreasing order of p-value, giving each time a smaller network G_k and a sparser precision matrix Λ_k, where k is the number of edges left. Note that removing an edge means setting the two corresponding entries (one in the upper triangular part of Λ and one in the lower triangular part), to 0. There are 11 variables, so there can be at most 55 edges.

The ROC and precision-recall (PR) curves were built by varying k from 55 to 0, and each time computing how many of the real non-edges were in G_k (FPR), how many of the real edges were in G_k (TPR, recall), and how many of the edges in G_k were real (precision).

Figure S14 shows the two plots described above for partial correlation networks on both numerical and rank data. As can be seen, the network on rank data slightly outperforms the network on numerical data. This is

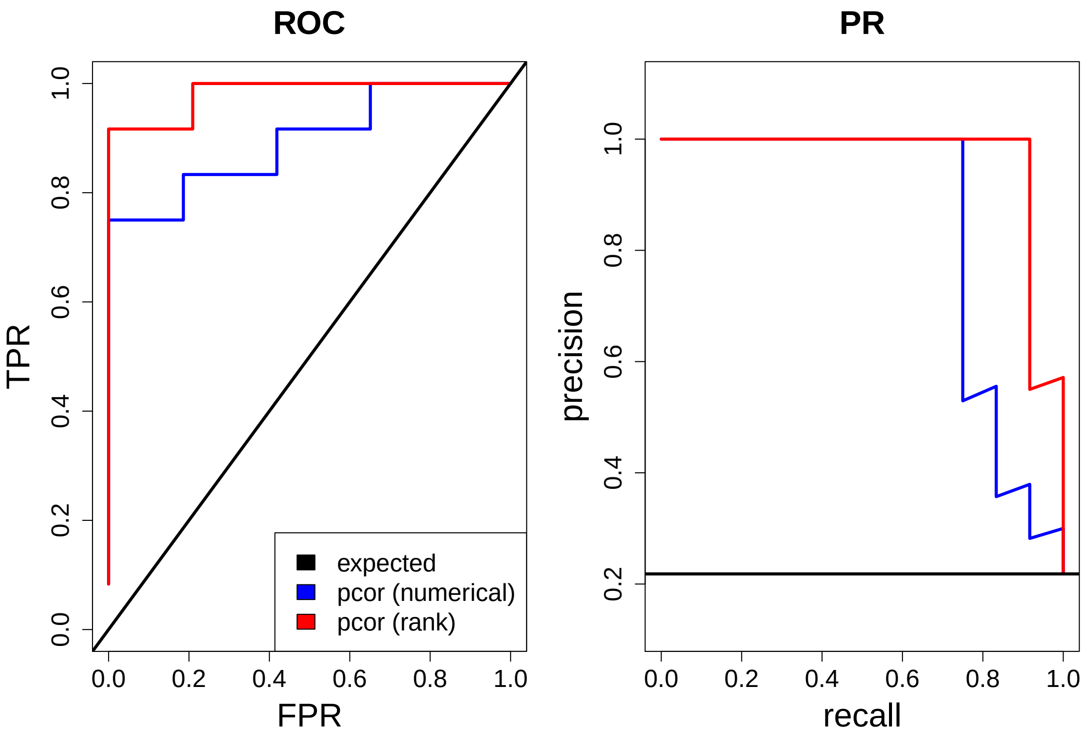

Figure S14: **Comparison of partial correlation matrices between rank data and numerical data on a non-Gaussian model.** Partial correlations are shown in purple, rank partial correlations in red. **Left:** ROC curve. The black diagonal line is the curve expected by chance. **Right:** Precision-recall (PR) curve. The black horizontal line is the curve expected by chance.

probably due to the fact that, in the presence of many samples N_G, the distribution of the rank data (uniform across $[1, N_G]$) can be approximated by a Gaussian with a very large variance, whereas the numerical data is far from Gaussian.

2nd simulation

A Bayesian network was designed as in Fig. S15 (left), and its corresponding undirected network constructed as in Fig. S15 (right).

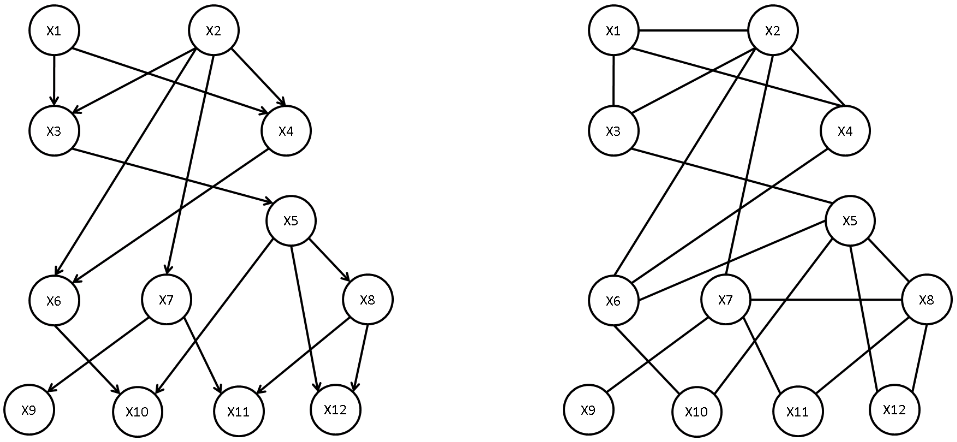

Figure S15: **Simulated networks. Left:** original Bayesian network used to generate data. **Right:** the corresponding undirected network the methods should find.

The Bayesian network was used to generate 5000 datapoints, according to the following distributions:

$$
\begin{aligned}
\mu &\sim \mathcal{N}\left(0, 3\ I_D\right) \\
\log\left(\sigma\right) &\sim \mathcal{N}\left(0, 2\ I_D\right) \\
\text{class} &\sim \text{ the first 1800 points are in class 1, the other 3200 in class 2}
\end{aligned}
$$

$$
\begin{aligned}
X_1 &\sim \mathcal{N}\left(-2, 1\right)\ \text{ for points in class 1} \\
&\ \mathcal{N}\left(2, 1\right)\ \text{ for points in class 2} \\
X_2 &\sim \mathcal{N}\left(-2, 1\right)\ \text{ for points in class 1} \\
&\ \mathcal{N}\left(2, 1\right)\ \text{ for points in class 2} \\
X_3 &\sim \mathcal{N}\left(2X_1 + 3X_2\ ,\ 150\ \sigma_3\right) \\
X_4 &\sim \mathcal{N}\left(\exp\left(\frac{\mu_4 + X_1 + 2X_2}{10}\right)\ ,\ \sigma_4\right) \\
X_5 &\sim \mathcal{N}\left(\exp\left(\frac{-X_3}{20}\right)\ ,\ \sigma_5\right) \\
X_6 &\sim \mathcal{N}\left(\frac{X_2}{15} + \log\left(X_4 + 5\right)\ ,\ 0.2\ \sigma_6\right)
\end{aligned}
$$

$$
\begin{aligned}
X_7 &\sim \mathcal{N}\left(\mu_7 + X_2\ ,\ 4\ \sigma_7\right) \\
X_8 &\sim \mathcal{N}\left(\mu_8 + X_5\ ,\ 5\ \sigma_8\right) \\
\log\left(X_9\right) &\sim \mathcal{N}\left(\frac{X_7}{30}\ ,\ 0.2\ \sigma_9\right) \\
\log\left(X_{10}\right) &\sim \mathcal{N}\left(\frac{\mu_1 0 + 2X_5 - 10X_6}{100}\ ,\ 0.01\ \sigma_{10}\right) \\
\log\left(X_{11}\right) &\sim \mathcal{N}\left(\frac{\mu_{11} - 2X_7 - 2X_8}{50}\ ,\ \sigma_{11}\right) \\
\log\left(X_{12}\right) &\sim \mathcal{N}\left(\frac{\mu_{12} + X_5 + 2X_8}{50}\ ,\ \sigma_{12}\right)
\end{aligned}
$$

which led to the variables shown in Fig. S16.

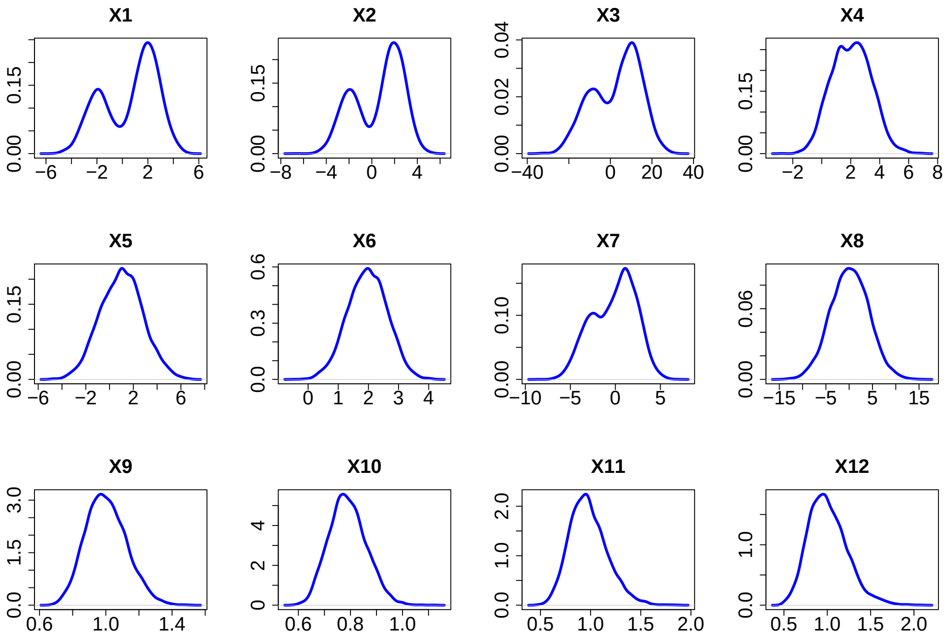

Figure S16: **Distributions of the toy variables.** On the x-axis are the values that can be taken by the variable under study, and on the y-axis the probability density.

The pairwise relationships between the variables are plotted in Fig. S17.

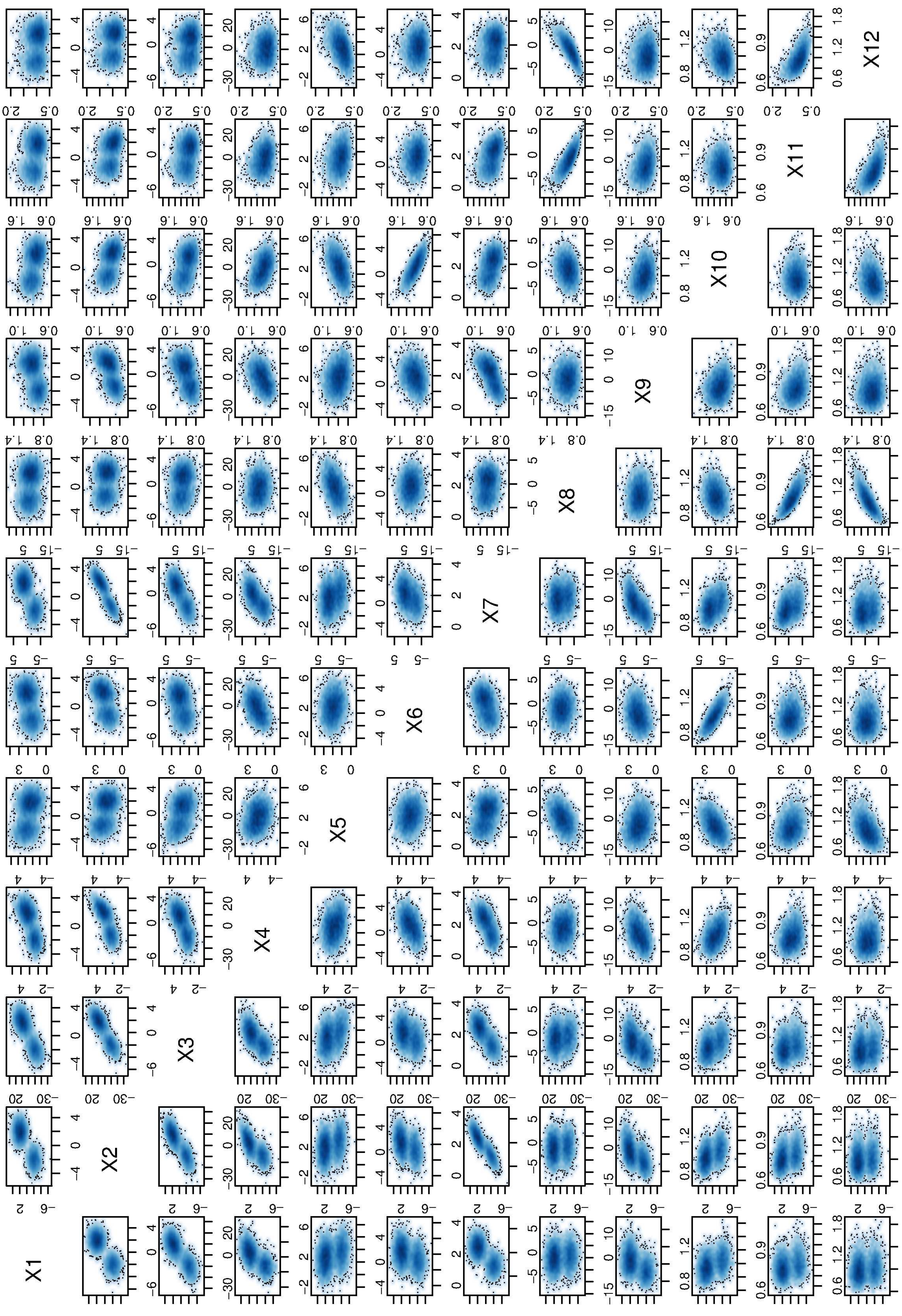

Figure S17: **Pairwise relationships between the toy variables.** The cell $M(i, j)$ in this matrix shows the scatter plot of variable X_i against variable X_j.

The PCM, its associated p-values and the precision matrix Λ (the inverse of the covariance matrix) were computed (the latter is an un-normalised version of the PCM). The p-values were ranked and the corresponding edges removed one at a time, in decreasing order of p-value, giving each time a smaller network G_k and a sparser precision matrix Λ_k, where k is the number of edges left. Note that removing an edge means setting the two corresponding entries (one in the upper triangular part of Λ and one in the lower triangular part), to 0. There are 12 variables, so there can be at most 66 edges.

The ROC and precision-recall (PR) curves were built by varying k from 66 to 0, and each time computing how many of the real non-edges were in G_k (FPR), how many of the real edges were in G_k (TPR, recall), and how many of the edges in G_k were real (precision).

Figure S18 shows the two plots described above for partial correlation networks on both numerical and rank data. As can be seen, the network on rank data and the network on numerical data perform very similaryly.

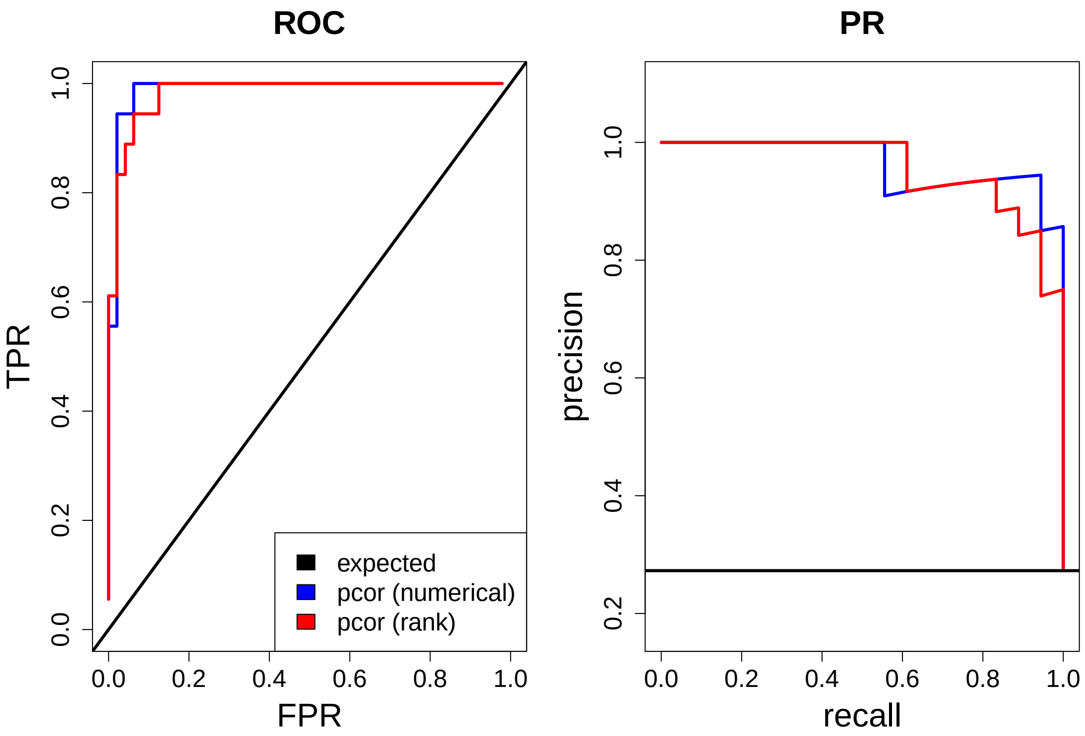

Figure S18: **Comparison of partial correlation matrices between rank data and numerical data on a non-Gaussian model.** Partial correlations are shown in purple, rank partial correlations in red. **Left:** ROC curve. The black diagonal line is the curve expected by chance. **Right:** Precision-recall (PR) curve. The black horizontal line is the curve expected by chance.

5.3.2 CD4+ data

Figure S19 shows the overlap between the PCMs obtained with numerical data and rank data. It is very high (for the size of our network: 42 edges, it is about 85%).

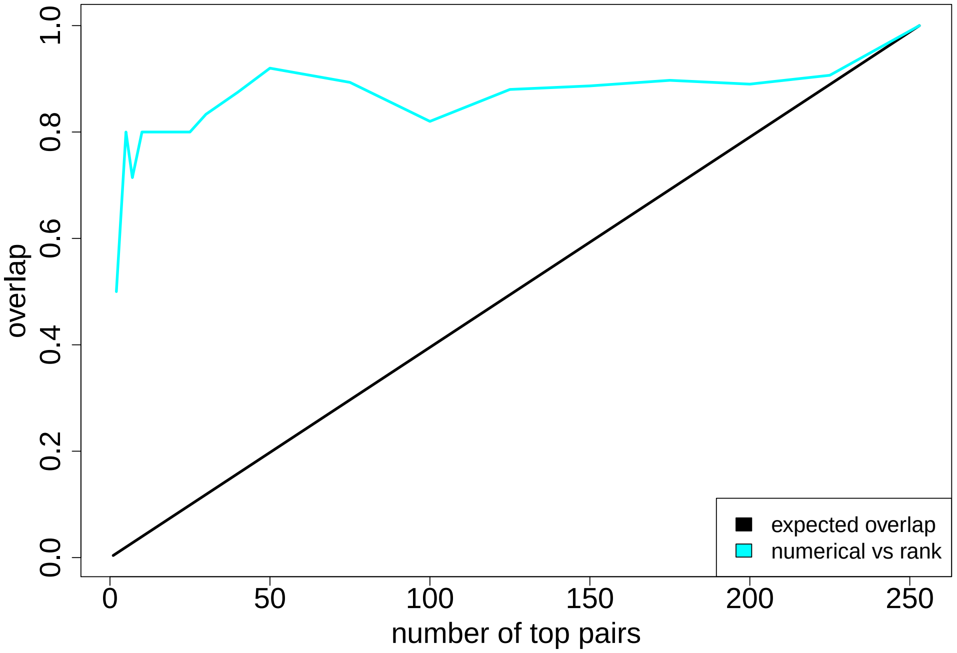

Figure S19: **Comparison of partial correlation matrices between rank data and numerical data on CD4+ data.** The overlap between networks on numerical data and rank data is shown in cyan. The black diagonal line is the overlap expected by chance.

We have called the sparsifying procedure on the numerical PCM and obtained a network with 47 edges. The differences with the rank network (42 edges) are listed in Table S7. The network based on numerical data loses the edge mRNA-H3K27ac and gains instead the edge mRNA-H3K4me3. Either edge is plausible, however H3K27ac is directly related to the levels of mRNA and was shown to be the best single predictor [12], whereas H3K4me3 was shown to be related to mRNA levels in low-CpG-promoter genes only. The additions H3K27me3-H3K79me2, H3K36me3-H3K9me3 and H3K4me1-H3K9ac are plausible. However the last addition H3K4me3-H3K9me3 seems a little strange because of the sign. H3K9me3 should represent constitutive heterochromatin and should not be positively associated with H3K4me3. Other additions and deletions involve acetylations only and do not really seem critical in regulation, so it is difficult to interpret them.

type	pair	numerical pcor	rank pcor
deleted edge	mRNA - H3K27ac	0.09012539	0.08840979
added edge	mRNA - H3K4me3	0.09688382	0.06294204
added edge	DNaseIHS - H2BK12ac	0.1460755	0.1504671
added edge	H2BK120ac - H2BK12ac	0.1484418	0.1406839
added edge	H3K18ac - H4K5ac	0.1443524	0.1211656
deleted edge	H3K18ac - H4K91ac	0.1414867	0.1578807
deleted edge	H3K27ac - H4K5ac	0.1328442	0.1493066
added edge	H3K27me3 - H3K79me2	-0.1609432	-0.1138062
added edge	H3K36me3 - H3K9me3	0.1700819	0.1203280
added edge	H3K4me1 - H3K9ac	-0.1887855	-0.1053897
added edge	H3K4me3 - H3K9me3	0.1578053	0.1262903

Table S7: **Differences between the partial correlation networks on rank data and numerical data.**

5.4 PCNs' sparseness: p-value threshold against GLASSO

To check whether setting a p-value threshold on the PCM was a viable method, we completed the simulation studies with a comparison with GLASSO.

- The ROC and precision-recall (PR) curves were computed as above.
- The likelihood was computed assuming a Gaussian model, i.e. all variables should be normally distributed and linear combinations of each other. This model does not fit the data, however it is the model assumed by both partial correlations and GLASSO. The likelihood was averaged over 10 cross-validation folds and expected to increase with the number of edges.
- The error was computed by performing linear regression for each variable using the variables that were connected to it in G_k only, and by averaging the prediction errors over all variables X_i and over 10 cross-validation folds. The error was expected to decrease with the number of edges.

For GLASSO, the procedure was similar. The penalty ρ was varied appropriately, each time giving a sparser version of the precision matrix Λ_ρ and a smaller network G_ρ. Note that here we could not control the number of edges and it may not always monotonically decrease as the penalty ρ increases, explaining some bumps in the curves. The computation of the four curves was unchanged.

Figure S20 shows the four plots described above for both partial correlations and GLASSO in the first simulation. As can be seen, the p-value threshold outperforms GLASSO.

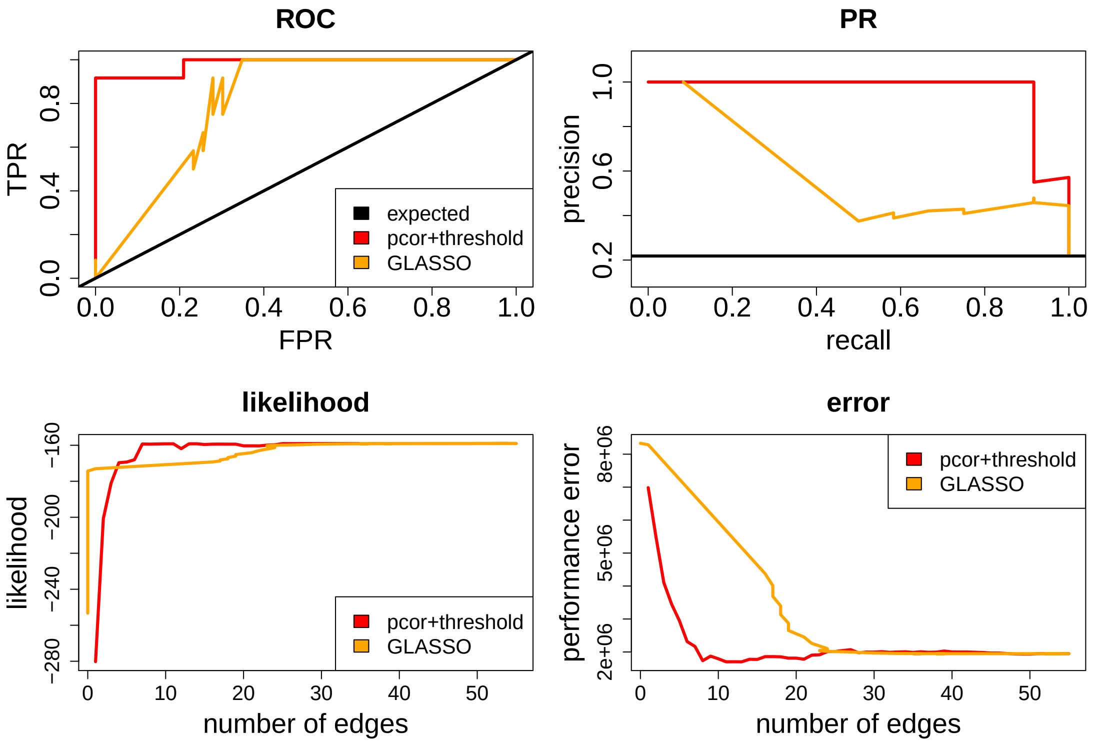

Figure S20: **Evaluation of partial correlations and GLASSO on a non-Gaussian model.** Partial correlations are shown in red, GLASSO in orange. **Top left:** ROC curve. The black diagonal line is the curve expected by chance. **Top right:** Precision-recall (PR) curve. The black horizontal line is the curve expected by chance. **Bottom left:** likelihood. **Bottom right:** prediction error.

We repeated the previous experiment on numerical data. Figure S21 shows the previous results again, this time with PCNs on both numerical data and rank data. A p-value threshold still outperforms GLASSO. Note that GLASSO does not seem to handle rank data well. This is probably due to the fact that the variance is very

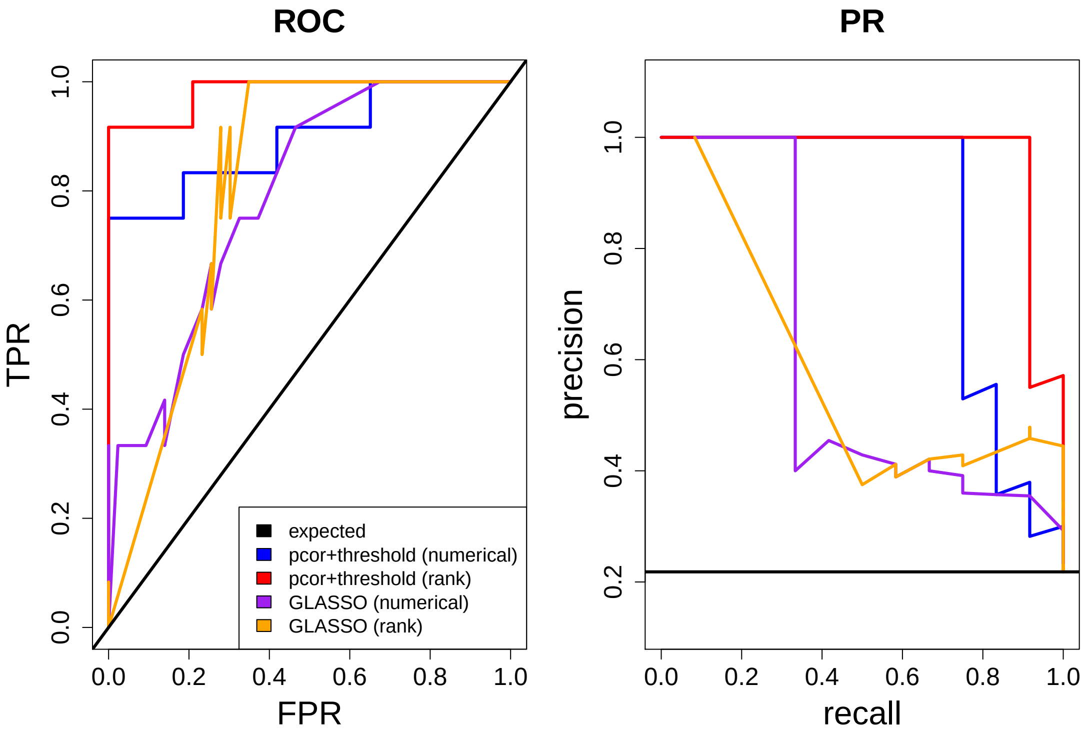

Figure S21: **Comparison of networks between rank data and numerical data.** Partial correlations on rank data are shown in purple, on numerical data in blue, GLASSO on rank data in orange, and GLASSO on numerical data in red. **Left:** ROC curve. The black diagonal line is the curve expected by chance. **Right:** Precision-recall (PR) curve. The black horizontal line is the curve expected by chance.

large and may create singularities.

Figure S22 shows the four plots described above for both partial correlations and GLASSO in the second simulation.

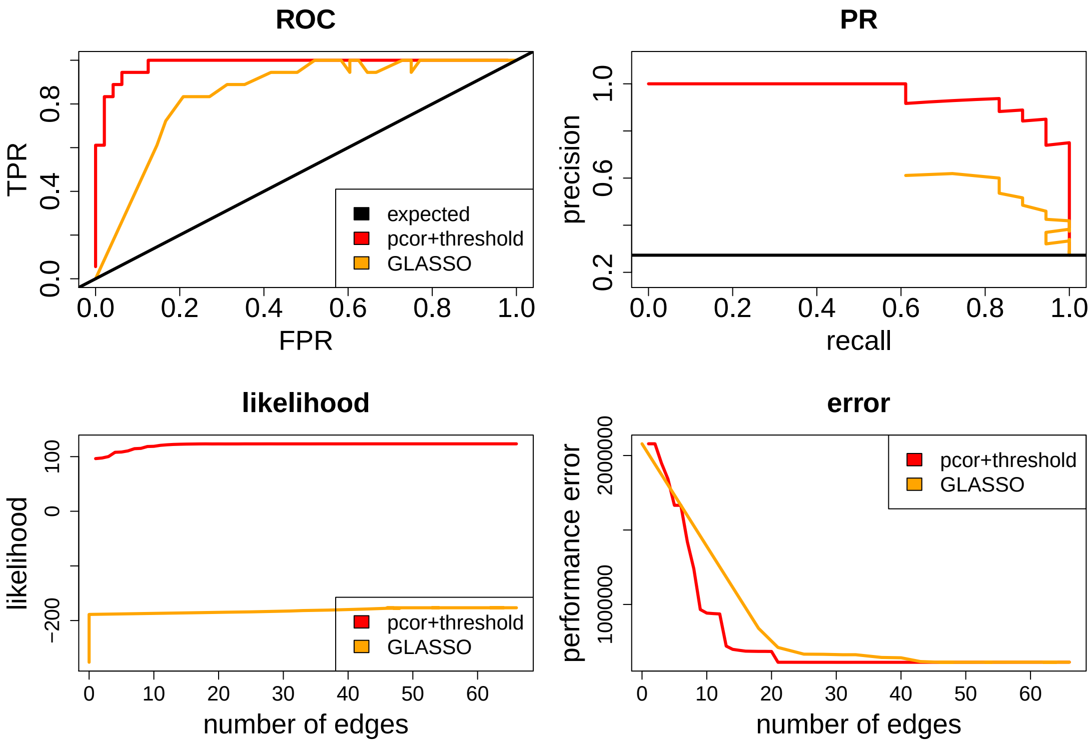

Figure S22: **Evaluation of partial correlations and GLASSO on a non-Gaussian model.** Partial correlations are shown in red, GLASSO in orange. **Top left:** ROC curve. The black diagonal line is the curve expected by chance. **Top right:** Precision-recall (PR) curve. The black horizontal line is the curve expected by chance. **Bottom left:** likelihood. **Bottom right:** prediction error.

Again, GLASSO is outperformed.

We repeated the previous experiment on numerical data. Figure S23 shows the previous results again, this time with PCNs on both numerical data and rank data. Again, GLASSO is outperformed.

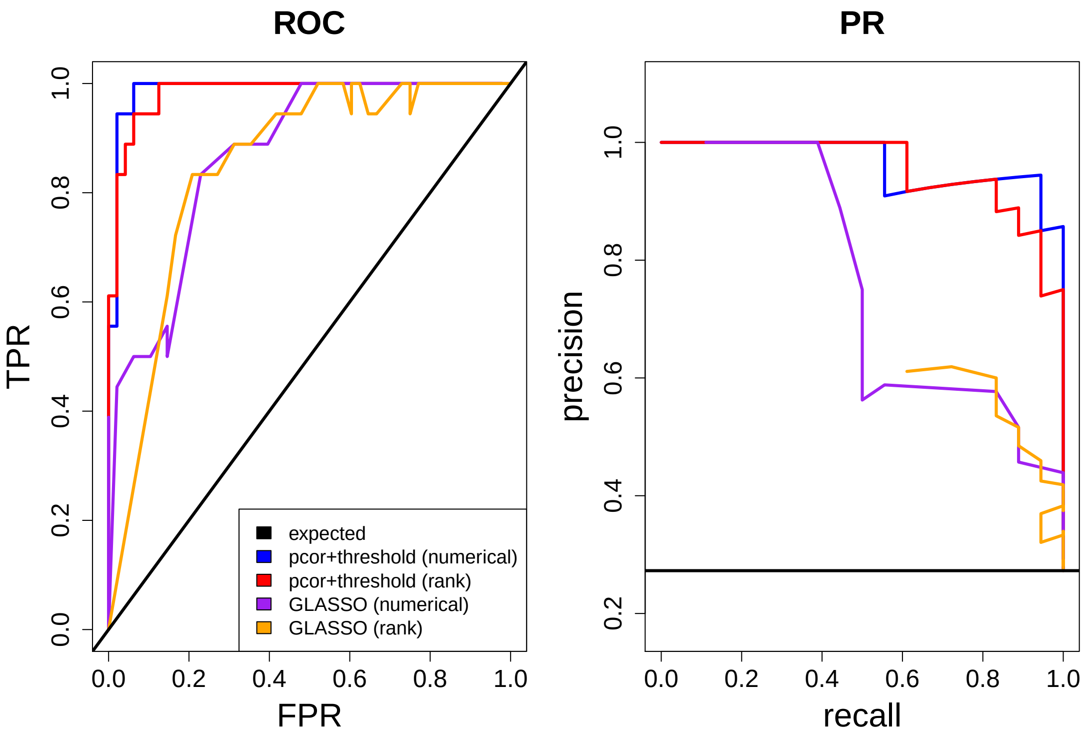

Figure S23: **Comparison of networks between rank data and numerical data.** Partial correlations on rank data are shown in purple, on numerical data in blue, GLASSO on rank data in orange, and GLASSO on numerical data in red. **Left:** ROC curve. The black diagonal line is the curve expected by chance. **Right:** Precision-recall (PR) curve. The black horizontal line is the curve expected by chance.

5.5 Use of rank data instead of discrete data

We binarized each variable using Gaussian mixture models with 2 modes and non-equal variances, using the R package mclust. We then applied a number of network contruction methods.

1) Firstly, we comupted the normalized mutual information matrix $\mathbf{M}$, where $M_{ij} = \dfrac{I(X_i, X_j)}{\sqrt{H(X_i)H(X_j)}}$, with I the mutual information measure, and H the entropy measure.

2) Secondly, we used the normalized mutual information matrix to build a network using the ARACNE algorithm [13]. Though the matrix returned by ARACNE already contains some 0s, we considered it as an input matrix and ran the algorithm to select a threshold and obtain a sparse network.

3) Thirdly, for a fairer comparison with partial correlations, we computed the normalized conditional mutual information matrix $\mathbf{CM}$, where $CM_{ij} = \dfrac{I(X_i, X_j|Z = \text{all other variables})}{\sqrt{H(X_i|Z)H(X_j|Z)}}$. Note that, in our dataset, there are 23 variables. Z therefore contains 21 variables, which define a space with 2^{21} possible states. The first problem is that we have more states than genes (datapoints), in other words the sampling is not large enough. This makes us question the reliability of the coefficients.

4) Fourthly, we also computed the partial correlations on the discrete data. This is wrong, as the linear relationships comes from the bimodality (in 2D, a line between 2 spheres) and not from the quantities (in 2D, a line across points). Nevertheless, since the linear relationships within modes tend to be close to the line between modes, we thought it was worth investigating.

Figure S24 shows the overlap between the networks obtained with various transformations of the CD4+ data: rank data, numerical data and discrete data. The trends are the same whether the data is discretized in 2 groups (top row) or 3 groups (bottom row). As seen earlier, the similarity between the network on numerical data and the network on rank data is very high (for the size of our network: 42 edges, it is about 85%).When using partial correlations to produce the discrete network, the overlap with the networks built on numerical and rank data is about 55%.

The overlap between the MI network or the ARACNE network on discrete data and numerical or rank data is significant, but much lower (about 30%). It increases up to about 40% with conditional mutual information. This makes sense as conditional mutual information embeds the idea of partial correlations to control for other variables. The differences are potentially due to the information lost through discretization, and to the difficulty of computing a meaningful conditional mutual information with so many variables in the condition.

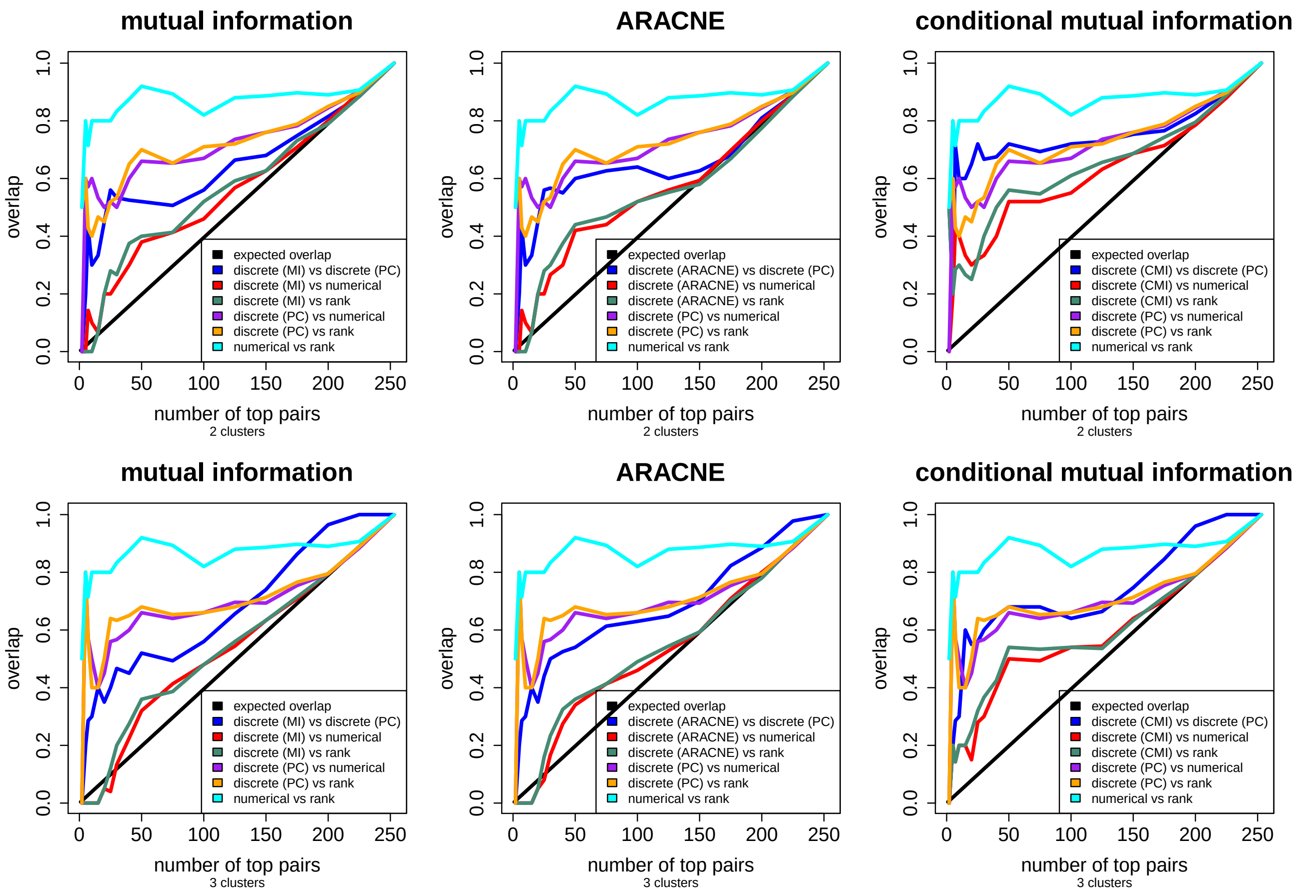

Figure S24: **Comparison of rank data, numerical data and discrete data.** The overlap between networks on discrete data (PC) and numerical data is always shown in purple, between networks on discrete data (PC) and rank data in orange, and between networks on numerical and rank data in cyan. The black diagonal line is the overlap expected by chance. **Left column:** Comparison with the network based on the normalized mutual information matrix (MI). **Center column:** Comparison with the network obtained after applying ARACNE. **Right column:** Comparison with the network based on the normalized conditional mutual information matrix (CMI). **Top:** the data is binarized. **Bottom:** all features are discretized in 3 groups.

For each of the 4 matrices, we built the associated sparse network, again using prediction error. Note however that, the variables being binary, we used logistic regression to predict the nodes, not linear regression, and we defined the error as the negation of the binomial likelihood. Figure S25 shows the networks. First of all, note that the three networks based on mutual information are much denser, which seems to indicate that many more features are necessary to predict each node, probably because they are not well chosen or because they do not contain enough information. Either way, it makes the result slightly doubtable. More importantly, the connections are not meaningful. Of course, since many more connections are present, many of them are plausible, but many of them also are not. If we take a closer look at the bottom left network based on conditional mutual information for example, mRNA is only connected to H2BK20ac, H3K18ac, H2BK120ac, acetylation marks that are not particularly known to be related to transcription.

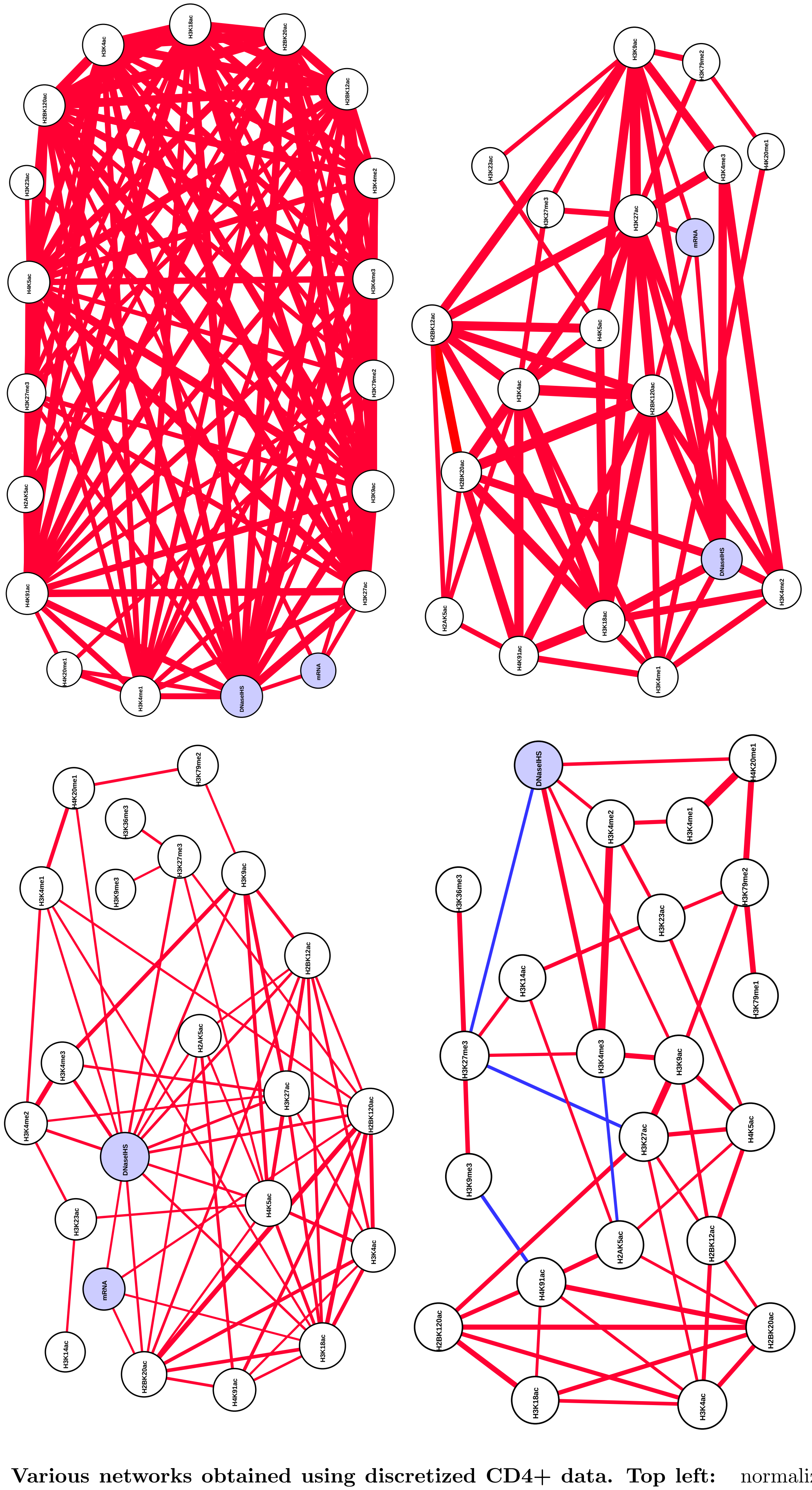

Figure S25: **Various networks obtained using discretized CD4+ data. Top left:** normalized mutual information network. **Top right:** ARACNE network. **Bottom left:** normalized conditional mutual information network. **Bottom right:** partial correlations network.

6 Controls

6.1 ChIP-seq controls using data common to Zhao, Roadmap and ENCODE

In order to make sure that the variability between cell types was not due to a bias in the ChIP-seq data, a second set of PCMs was created using appropriate controls. For CD4+ cells, the data was generated using MNase ChIP-seq, so MNase was used as control variable. For IMR90 and H1 cells, the data was generated using sonication ChIP-seq, so Input was used as control variable. Note that the PCMs are no longer strictly comparable, since they have different variables, but we focus on the comparable part, i.e. the 10 histone modifications, mRNA and DNaseIHS.

Figure S26 shows the overlap between the top pairs obtained when using different cell types. The solid lines represent the case when no ChIP-seq control is used, the dashed lines the case when either MNase (for CD4+ cells) or Input are used as ChIP-seq control. In each subplot, the two curves are very similar, suggesting that the

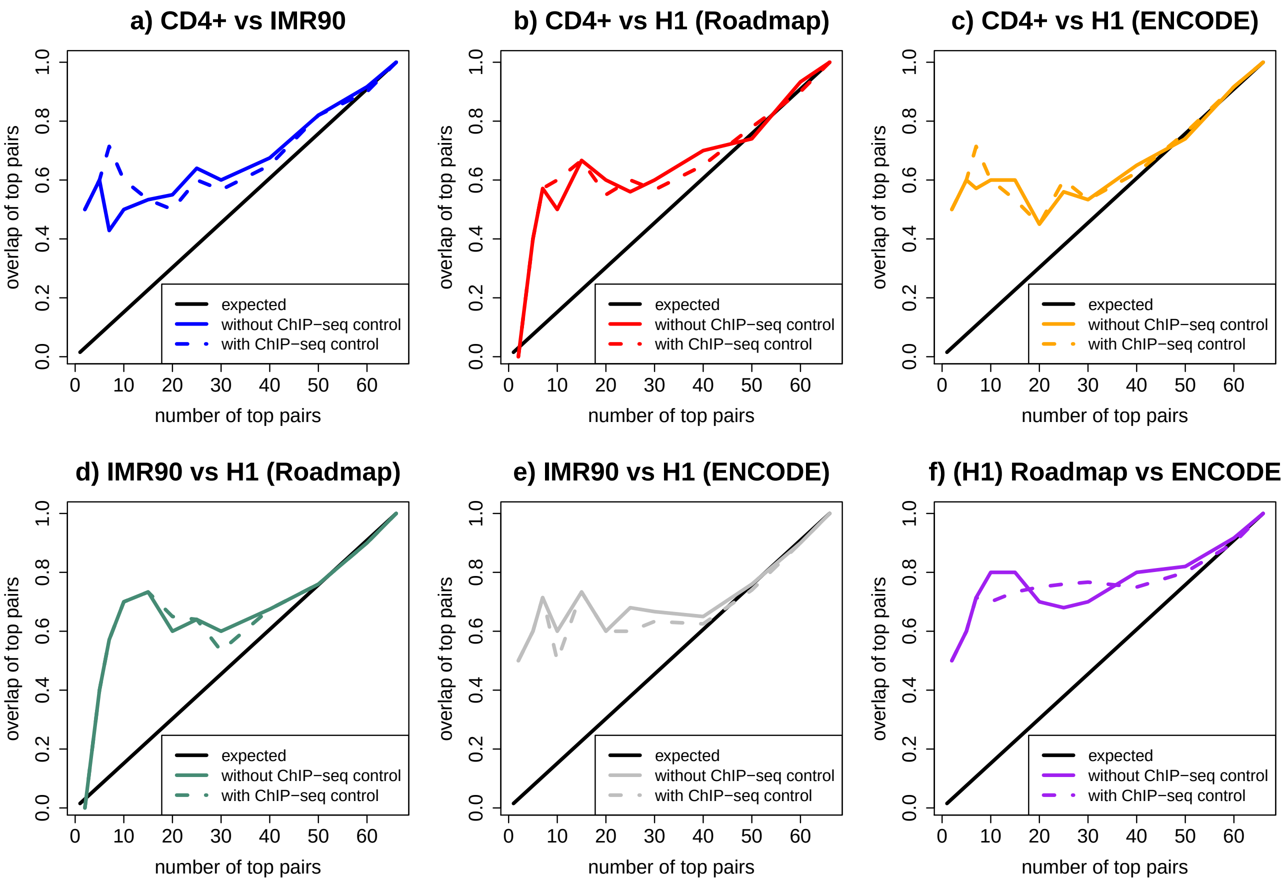

Figure S26: **Influence of ChIP-seq control on the similarity between experiments and cell types.** All plots have the same construction. The x-axis shows the number of top pairs that are considered k. The y-axis shows the proportion of these pairs that are found in the two lists that are considered, as an estimate of the similarity between partial correlation matrices. The solid lines represent the case when no ChIP-seq control is used, the dashed lines the case when either MNase (for CD4+ cells) or Input are used as ChIP-seq control. **a)** Overlap between CD4+ and IMR90. **b)** Overlap between CD4+ and H1 (Roadmap). **c)** Overlap between CD4+ and H1 (ENCODE). **d)** Overlap between IMR90 and H1 (Roadmap). **e)** Overlap between IMR90 and H1 (ENCODE). **f)** Overlap - within H1 cells - between Roadmap and ENCODE.

ChIP-seq has little influence on the results, and that the differences in cell types are not due to the CHIP-seq method.

6.2 ChIP-seq controls using data common to Zhao and Roadmap

In order to make sure that the variability between cell types was not due to a bias in the ChIP-seq data, a second set of PCMs was created using appropriate controls. For CD4+ cells, the data was generated using MNase ChIP-seq, so MNase was used as control variable. For IMR90 and H1 cells, the data was generated using sonication ChIP-seq, so Input was used as control variable. Note that the PCMs are no longer strictly comparable, since they have different variables, but we focus on the comparable part, i.e. the 21 histone modifications, mRNA and DNaseIHS.

Figure S27 shows the overlap between the top pairs obtained when using different cell types. The solid lines represent the case when no ChIP-seq control is used, the dashed lines the case when either MNase (for CD4+ cells) or Input are used as ChIP-seq control.

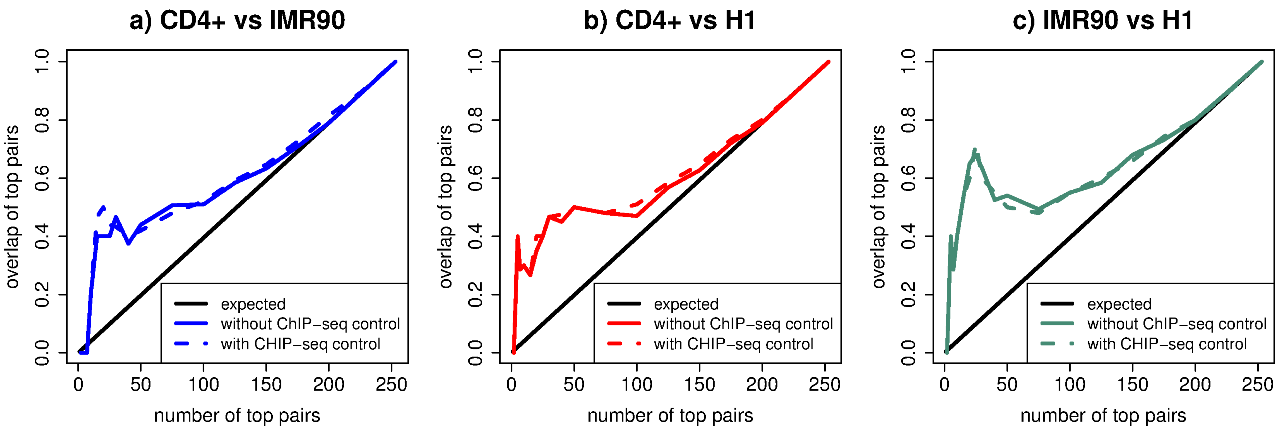

Figure S27: **Influence of ChIP-seq control on the similarity between cell types.** All plots have the same construction. The x-axis shows the number of top pairs that are considered k. The y-axis shows the proportion of these pairs that are found in the two lists that are considered, as an estimate of the similarity between partial correlation matrices. The solid lines represent the case when no ChIP-seq control is used, the dashed lines the case when either MNase (for CD4+ cells) or Input are used as ChIP-seq control. **a)** Overlap between CD4+ and IMR90. **b)** Overlap between CD4+ and H1. **c)** Overlap between IMR90 and H1.

7 The three networks

All networks here are presented as a list of edges with their properties.

7.1 Network for CD4+ cells

variable 1	variable 2	Cor	PCor	p-value	q-value	draw
H3K4me1	H3K4me2	0.81	0.42	0	0	✓
H3K79me1	H3K79me2	0.78	0.38	0	0	✓
H4K20me1	H4K5ac	0.5	-0.28	2.9e-238	1.5e-236	✓
H3K27ac	H3K27me3	-0.67	-0.26	1.2e-197	4.4e-196	✓
H3K4me3	H3K9ac	0.88	0.26	9.0e-191	2.5e-189	✓
DNaseIHS	H3K4me3	0.85	0.25	1.8e-187	4.2e-186	✓
mRNA	H3K27me3	-0.61	-0.25	4.4e-187	8.5e-186	✓
H2BK20ac	H4K91ac	0.95	0.22	1.5e-143	2.6e-142	✓
H2BK120ac	H4K91ac	0.95	0.21	2.5e-132	3.7e-131	✓
H3K23ac	H3K4me2	0.7	0.21	1.8e-130	2.4e-129	✓
H2BK120ac	H2BK20ac	0.96	0.21	3.8e-127	4.6e-126	✓
H2BK20ac	H3K18ac	0.95	0.19	2.8e-109	3.1e-108	✓
H2AK5ac	H2BK20ac	0.76	0.19	5.6e-103	5.6e-102	✓
H3K27me3	H3K4me3	-0.49	0.18	7.3e-95	6.9e-94	✓
H3K79me2	H4K20me1	0.62	0.17	1.5e-81	1.3e-80	✓
H3K18ac	H3K4ac	0.95	0.17	3.7e-80	3.0e-79	✓
H3K36me3	H3K79me1	0.16	0.16	4.8e-76	3.7e-75	✓
H3K14ac	H3K27me3	-0.09	0.16	3.6e-73	2.6e-72	✓
H3K18ac	H4K91ac	0.95	0.16	3.7e-72	2.5e-71	✓
DNaseIHS	H2BK12ac	0.84	0.15	1.3e-65	8.5e-65	
H2BK20ac	H3K4ac	0.95	0.15	6.6e-64	4.1e-63	
H3K4me1	H3K4me3	0.68	-0.14	9.1e-57	5.4e-56	
H3K14ac	H4K5ac	0.5	0.13	9.4e-51	5.3e-50	
H3K4me3	H3K9me3	-0.21	0.13	1.5e-46	8.1e-46	
H3K18ac	H4K5ac	0.92	0.12	5.9e-43	3.1e-42	
mRNA	H3K79me2	0.62	0.12	3.5e-42	1.7e-41	✓
H3K23ac	H4K91ac	0.6	-0.11	1.4e-37	6.5e-37	
mRNA	H4K20me1	0.57	0.11	3.3e-34	1.5e-33	✓
H3K4me1	H3K9ac	0.66	-0.11	7.7e-33	3.4e-32	
H2BK120ac	H3K27ac	0.93	0.1	6.2e-29	2.6e-28	
H3K27ac	H4K20me1	0.57	-0.1	9.5e-28	3.9e-27	
H2AK5ac	H3K4ac	0.75	0.1	4.4e-27	1.8e-26	
H3K4ac	H3K9ac	0.9	0.09	1.2e-26	4.8e-26	
H3K18ac	H3K27me3	-0.6	0.09	3.0e-26	1.1e-25	
H3K23ac	H3K27me3	-0.33	0.09	7.2e-26	2.6e-25	
H3K4me3	H4K5ac	0.79	-0.09	1.3e-25	4.6e-25	
H2BK12ac	H3K9me3	-0.32	0.09	3.5e-25	1.2e-24	
mRNA	H3K27ac	0.66	0.09	1.5e-23	5.1e-23	✓
H2BK20ac	H3K27me3	-0.6	0.09	6.3e-23	2.1e-22	
H2BK12ac	H3K4ac	0.93	0.09	6.7e-22	2.1e-21	
H3K18ac	H3K36me3	-0.01	-0.08	1.3e-21	4.2e-21	
H3K4me2	H4K5ac	0.8	0.08	7.6e-21	2.3e-20	
H3K9me3	H4K20me1	-0.35	-0.08	5.4e-19	1.6e-18	
H3K4ac	H4K5ac	0.92	0.08	4.2e-18	1.2e-17	
H3K18ac	H3K4me1	0.74	0.08	1.2e-17	3.3e-17	
H2BK120ac	H3K36me3	-0.02	-0.07	8.8e-17	2.4e-16	
H3K23ac	H3K9ac	0.7	0.07	2.2e-16	5.9e-16	
H2AK5ac	H3K9ac	0.63	-0.07	3.1e-16	8.3e-16	
DNaseIHS	H3K9ac	0.85	0.07	6.0e-16	1.6e-15	
H3K4ac	H3K79me2	0.74	0.07	4.1e-15	1.1e-14	
H3K14ac	H3K9ac	0.44	0.07	5.2e-15	1.3e-14	
DNaseIHS	H3K4me2	0.82	0.07	1.5e-14	3.6e-14	
H3K14ac	H3K4me2	0.42	0.07	2.6e-14	6.2e-14	
H2BK20ac	H3K9ac	0.88	-0.07	1.5e-13	3.6e-13	
H3K79me1	H4K5ac	0.71	0.06	2.4e-13	5.6e-13	
mRNA	H3K18ac	0.61	-0.06	5.5e-13	1.3e-12	
mRNA	H3K4me3	0.6	0.06	1.1e-12	2.6e-12	
H4K5ac	H4K91ac	0.9	0.06	2.4e-12	5.2e-12	
H2BK20ac	H4K5ac	0.91	0.06	8.5e-12	1.8e-11	
H3K4me1	H4K5ac	0.68	0.06	2.7e-11	5.7e-11	
H2BK120ac	H3K9me3	-0.35	0.06	5.1e-11	1.1e-10	
DNaseIHS	H3K79me1	0.71	-0.06	1.3e-10	2.8e-10	
H2BK12ac	H3K4me1	0.67	-0.05	8.8e-10	1.8e-09	
H3K18ac	H3K9me3	-0.36	-0.05	1.3e-09	2.6e-09	
H3K14ac	H4K91ac	0.38	-0.05	1.6e-09	3.2e-09	
H3K4me1	H3K9me3	-0.38	-0.05	3.0e-09	5.8e-09	

variable 1	variable 2	Cor	PCor	p-value	q-value	draw
H3K4me2	H3K4me3	0.88	0.43	0	0	✓
H3K4me1	H4K20me1	0.79	0.39	0	0	✓
H2AK5ac	H3K4me3	0.49	-0.26	1.4e-201	5.7e-200	✓
H3K4ac	H4K91ac	0.95	0.26	4.0e-193	1.3e-191	✓
H3K4me1	H3K79me1	0.78	0.25	5.1e-188	1.3e-186	✓
H3K27me3	H3K9me3	0.48	0.25	2.9e-187	6.0e-186	✓
DNaseIHS	H4K20me1	0.69	0.23	2.9e-156	5.2e-155	✓
H3K27me3	H3K36me3	0.22	0.22	1.4e-140	2.2e-139	✓
H3K14ac	H3K23ac	0.56	0.21	8.4e-131	1.2e-129	✓
H2BK12ac	H2BK20ac	0.94	0.21	1.6e-128	2.0e-127	✓
H3K79me1	H4K20me1	0.73	0.2	3.4e-114	3.9e-113	✓
H3K27ac	H3K4me3	0.86	0.19	3.4e-105	3.5e-104	✓
H3K27ac	H3K9ac	0.92	0.19	1.8e-102	1.8e-101	✓
DNaseIHS	H3K14ac	0.28	-0.18	1.3e-88	1.2e-87	✓
H2AK5ac	H3K36me3	0.16	0.17	1.8e-80	1.5e-79	✓
H2BK120ac	H3K18ac	0.96	0.17	9.0e-80	7.1e-79	✓
H4K20me1	H4K91ac	0.66	0.16	3.3e-75	2.5e-74	✓
H3K4me1	H3K79me2	0.62	-0.16	3.7e-73	2.6e-72	✓
H3K23ac	H3K79me2	0.61	0.15	4.0e-68	2.7e-67	✓
H3K27ac	H4K5ac	0.91	0.15	1.3e-64	8.2e-64	✓
H2BK120ac	H2BK12ac	0.93	0.14	1.8e-57	1.1e-56	
H3K18ac	H3K4me3	0.85	0.13	1.0e-51	5.8e-51	
H3K23ac	H4K5ac	0.71	0.13	5.5e-49	3.0e-48	
H3K9ac	H4K5ac	0.89	0.12	1.9e-43	1.0e-42	
H3K36me3	H3K9me3	0.14	0.12	2.2e-42	1.1e-41	
H3K27me3	H3K79me2	-0.56	-0.11	4.7e-38	2.3e-37	
H3K23ac	H4K20me1	0.32	-0.11	9.0e-36	4.2e-35	
H3K36me3	H4K5ac	0.03	0.11	4.1e-33	1.9e-32	
H2BK120ac	H3K4me3	0.85	0.11	8.1e-33	3.5e-32	
H2BK12ac	H4K5ac	0.91	0.1	1.3e-28	5.3e-28	
H2AK5ac	H2BK12ac	0.73	0.1	1.1e-27	4.4e-27	
H2BK120ac	H3K4ac	0.95	0.1	4.7e-27	1.8e-26	
H2BK120ac	H4K5ac	0.92	0.09	2.3e-26	9.0e-26	
H3K18ac	H3K27ac	0.93	0.09	3.0e-26	1.1e-25	
H3K36me3	H4K20me1	0.08	0.09	9.8e-26	3.5e-25	
H2BK12ac	H4K20me1	0.55	-0.09	1.6e-25	5.7e-25	
H2BK120ac	H3K9ac	0.91	0.09	3.7e-25	1.3e-24	
H2BK12ac	H3K79me2	0.72	0.09	3.0e-23	9.9e-23	
H3K4me2	H4K91ac	0.79	-0.09	4.3e-22	1.4e-21	
H2AK5ac	H3K14ac	0.41	0.09	6.6e-22	2.1e-21	
H2BK12ac	H3K27ac	0.91	0.08	4.0e-21	1.2e-20	
H3K14ac	H3K36me3	0.2	0.08	1.8e-20	5.4e-20	
H2AK5ac	H3K23ac	0.51	0.08	2.0e-18	5.8e-18	
H3K27ac	H3K4ac	0.92	0.08	9.9e-18	2.9e-17	
H2BK12ac	H3K4me3	0.79	-0.07	2.5e-17	7.0e-17	
H3K79me1	H3K9ac	0.72	0.07	1.1e-16	3.1e-16	
DNaseIHS	H2BK20ac	0.85	0.07	2.5e-16	6.7e-16	
H2AK5ac	H3K9me3	-0.34	-0.07	4.4e-16	1.2e-15	
H3K9ac	H4K91ac	0.89	0.07	3.1e-15	7.9e-15	
H3K27me3	H4K5ac	-0.6	-0.07	4.5e-15	1.1e-14	
H3K18ac	H3K79me1	0.73	-0.07	9.3e-15	2.3e-14	
H3K79me2	H3K9ac	0.75	0.07	2.5e-14	6.1e-14	
H3K79me1	H4K91ac	0.75	0.07	5.4e-14	1.3e-13	
H2BK12ac	H3K18ac	0.93	0.06	2.1e-13	4.9e-13	
H3K14ac	H3K79me2	0.38	0.06	2.4e-13	5.6e-13	
H3K79me2	H3K9me3	-0.26	0.06	7.4e-13	1.7e-12	
DNaseIHS	H3K27me3	-0.6	-0.06	1.3e-12	2.8e-12	
H3K9me3	H4K91ac	-0.4	-0.06	2.9e-12	6.4e-12	
H3K4me2	H3K79me1	0.76	0.06	8.5e-12	1.8e-11	
H3K27me3	H4K91ac	-0.64	-0.06	3.2e-11	6.7e-11	
H2AK5ac	H4K91ac	0.74	0.06	6.2e-11	1.3e-10	
H3K27ac	H3K79me2	0.75	0.06	1.5e-10	3.1e-10	
H3K4me2	H3K9me3	-0.3	-0.05	1.2e-09	2.4e-09	
H2AK5ac	H3K4me1	0.6	0.05	1.3e-09	2.7e-09	
H3K4ac	H3K4me2	0.8	-0.05	1.8e-09	3.5e-09	
H3K4me3	H4K20me1	0.6	0.05	3.1e-09	5.9e-09	

H2BK20ac	H3K79me2	0.71	-0.05	6.9e-09	1.3e-08	
H2AK5ac	H3K79me1	0.61	0.05	9.9e-09	1.9e-08	
H2AK5ac	H4K5ac	0.72	0.05	1.5e-08	2.8e-08	
H3K18ac	H3K79me2	0.72	-0.05	1.7e-08	3.1e-08	
H3K23ac	H3K27ac	0.69	0.05	4.0e-08	7.2e-08	
H2BK120ac	H3K79me1	0.73	-0.05	8.9e-08	1.6e-07	
H2AK5ac	H3K27me3	-0.45	0.05	1.9e-07	3.3e-07	
H2BK12ac	H3K27me3	-0.62	-0.05	2.9e-07	4.9e-07	
mRNA	H3K14ac	0.19	-0.04	6.6e-07	1.1e-06	
H3K14ac	H3K79me1	0.37	0.04	1.1e-06	1.8e-06	
H3K14ac	H3K4ac	0.43	0.04	1.6e-06	2.7e-06	
H3K4me3	H4K91ac	0.8	-0.04	5.1e-06	8.3e-06	
H2BK120ac	H3K79me2	0.73	-0.04	1.1e-05	1.8e-05	
H2BK12ac	H3K23ac	0.66	0.04	1.7e-05	2.6e-05	
H3K27me3	H3K79me1	-0.52	-0.04	2.1e-05	3.2e-05	
H3K14ac	H3K18ac	0.43	0.04	5.1e-05	8.0e-05	
H3K18ac	H3K23ac	0.67	0.03	8.1e-05	1.2e-04	
H2AK5ac	H3K79me2	0.56	0.03	2.3e-04	3.5e-04	
H3K27me3	H3K4me1	-0.47	0.03	2.5e-04	3.8e-04	
H3K14ac	H3K4me3	0.38	-0.03	4.9e-04	7.3e-04	
H3K36me3	H3K79me2	0.06	-0.03	7.8e-04	0.001	
mRNA	H3K9ac	0.63	0.03	9.4e-04	0.001	
H2BK12ac	H3K9ac	0.89	0.03	0.001	0.002	
H3K23ac	H3K9me3	-0.13	0.03	0.001	0.002	
mRNA	H4K5ac	0.59	-0.03	0.002	0.003	
H3K27me3	H4K20me1	-0.44	0.03	0.002	0.003	
H3K36me3	H4K91ac	0	0.03	0.004	0.005	
H2BK12ac	H3K14ac	0.43	0.03	0.004	0.005	
H2BK12ac	H3K79me1	0.7	-0.02	0.007	0.01	
H3K4ac	H3K79me1	0.75	0.02	0.016	0.021	
H3K36me3	H3K4ac	0.01	0.02	0.018	0.024	
DNaseIHS	H4K5ac	0.81	0.02	0.023	0.03	
H3K23ac	H3K36me3	0.09	0.02	0.036	0.046	
H2AK5ac	H2BK120ac	0.71	-0.02	0.045	0.057	
H3K27ac	H3K4me2	0.83	0.02	0.058	0.073	
H3K27ac	H3K36me3	-0.02	0.02	0.064	0.079	
H2BK20ac	H3K4me1	0.73	0.02	0.087	0.107	
H3K27ac	H3K9me3	-0.33	0.01	0.103	0.126	
DNaseIHS	H2BK120ac	0.87	0.01	0.12	0.144	
H3K27me3	H3K4me2	-0.5	-0.01	0.126	0.152	
mRNA	H3K79me1	0.6	-0.01	0.135	0.16	
H2BK20ac	H3K36me3	0.01	-0.01	0.142	0.167	
H3K79me1	H3K9me3	-0.33	-0.01	0.146	0.17	
mRNA	H2BK120ac	0.63	0.01	0.19	0.22	
H3K27me3	H3K9ac	-0.6	-0.01	0.244	0.28	
H2BK12ac	H3K4me2	0.78	-0.01	0.325	0.368	
H3K23ac	H3K4ac	0.65	-0.01	0.344	0.387	
H3K14ac	H3K4me1	0.3	-0.01	0.377	0.42	
H3K9ac	H3K9me3	-0.3	-0.01	0.385	0.425	
H2BK20ac	H3K9me3	-0.36	-0.01	0.472	0.517	
mRNA	H3K9me3	-0.32	-0.01	0.507	0.55	
H3K79me2	H4K5ac	0.71	-0.01	0.566	0.609	
H3K4me1	H4K91ac	0.74	0	0.596	0.636	
H2BK120ac	H3K23ac	0.66	0	0.631	0.671	
mRNA	H2BK12ac	0.6	0	0.687	0.721	
H2BK20ac	H3K4me3	0.81	0	0.685	0.722	
H3K4me3	H3K79me1	0.69	0	0.738	0.762	
H2AK5ac	H3K18ac	0.72	0	0.862	0.883	
DNaseIHS	H4K91ac	0.85	0	0.917	0.931	
H3K4me2	H3K79me2	0.71	0	0.952	0.96	
DNaseIHS	H3K4me1	0.72	0	0.995	0.995	

H3K23ac	H3K4me3	0.68	0.05	7.4e-09	1.4e-08	
H3K9me3	H4K5ac	-0.34	-0.05	1.1e-08	2.1e-08	
H3K14ac	H4K20me1	0.14	-0.05	1.6e-08	2.9e-08	
H3K27me3	H3K4ac	-0.64	-0.05	1.8e-08	3.3e-08	
H2BK120ac	H3K4me1	0.73	0.05	6.4e-08	1.1e-07	
H3K4me3	H3K79me2	0.71	0.05	1.3e-07	2.3e-07	
DNaseIHS	H3K23ac	0.58	-0.05	2.7e-07	4.6e-07	
H3K18ac	H3K9ac	0.91	0.05	3.0e-07	5.1e-07	
DNaseIHS	H3K36me3	-0.04	-0.04	7.0e-07	1.2e-06	
mRNA	H3K36me3	-0.01	0.04	1.1e-06	1.9e-06	
H3K4ac	H4K20me1	0.62	0.04	3.2e-06	5.2e-06	
mRNA	H3K4me1	0.57	0.04	6.3e-06	1.0e-05	
H2BK120ac	H3K27me3	-0.63	-0.04	1.2e-05	2.0e-05	
mRNA	H2AK5ac	0.46	0.04	1.9e-05	3.0e-05	
H3K4ac	H3K4me3	0.81	-0.04	2.7e-05	4.3e-05	
H3K4me2	H3K9ac	0.82	-0.04	6.8e-05	1.1e-04	
H3K9ac	H4K20me1	0.56	-0.03	1.7e-04	2.6e-04	
H3K18ac	H3K4me2	0.83	0.03	2.5e-04	3.7e-04	
DNaseIHS	H3K27ac	0.86	0.03	3.1e-04	4.7e-04	
DNaseIHS	H3K79me2	0.71	0.03	6.2e-04	9.2e-04	
H3K79me2	H4K91ac	0.72	-0.03	9.2e-04	0.001	
H2BK20ac	H3K4me2	0.81	0.03	0.001	0.001	
H2BK12ac	H4K91ac	0.92	0.03	0.001	0.002	
mRNA	H2BK20ac	0.61	-0.03	0.002	0.003	
H2BK20ac	H3K14ac	0.42	-0.03	0.002	0.003	
H3K23ac	H3K4me1	0.49	-0.03	0.003	0.004	
H3K4ac	H3K9me3	-0.38	-0.03	0.004	0.005	
H3K18ac	H4K20me1	0.61	0.02	0.006	0.009	
H3K27ac	H3K79me1	0.73	0.02	0.009	0.012	
H3K14ac	H3K9me3	-0.03	0.02	0.016	0.021	
mRNA	H4K91ac	0.64	0.02	0.021	0.028	
H3K36me3	H3K4me1	0.08	-0.02	0.024	0.031	
H2BK120ac	H3K14ac	0.41	-0.02	0.038	0.048	
H3K4me2	H4K20me1	0.66	0.02	0.055	0.069	
mRNA	H3K4ac	0.64	0.02	0.06	0.076	
H3K23ac	H3K79me1	0.55	-0.02	0.082	0.101	
H2BK12ac	H3K36me3	-0.01	-0.01	0.099	0.121	
H3K14ac	H3K27ac	0.43	0.01	0.105	0.127	
H2BK20ac	H3K23ac	0.64	-0.01	0.12	0.145	
H2AK5ac	H3K27ac	0.66	-0.01	0.132	0.158	
H2BK120ac	H4K20me1	0.61	0.01	0.139	0.164	
H3K36me3	H3K4me2	0.04	-0.01	0.146	0.17	
mRNA	H3K4me2	0.6	0.01	0.163	0.189	
H3K27ac	H4K91ac	0.9	-0.01	0.206	0.237	
DNaseIHS	H3K4ac	0.85	-0.01	0.297	0.338	
H2BK20ac	H4K20me1	0.62	0.01	0.335	0.378	
DNaseIHS	H3K18ac	0.86	0.01	0.361	0.404	
DNaseIHS	H2AK5ac	0.6	0.01	0.382	0.424	
H2AK5ac	H3K4me2	0.57	0.01	0.392	0.431	
H2AK5ac	H4K20me1	0.48	-0.01	0.495	0.54	
H2BK20ac	H3K79me1	0.73	-0.01	0.544	0.588	
H3K27ac	H3K4me1	0.69	0	0.589	0.631	
H3K36me3	H3K9ac	0	0	0.632	0.669	
H3K36me3	H3K4me3	-0.01	0	0.691	0.72	
mRNA	H3K23ac	0.42	0	0.69	0.721	
H3K4ac	H3K4me1	0.73	0	0.711	0.737	
DNaseIHS	H3K9me3	-0.33	0	0.795	0.818	
H2BK20ac	H3K27ac	0.91	0	0.871	0.889	
H2BK120ac	H3K4me2	0.82	0	0.932	0.943	
mRNA	DNaseIHS	0.63	0	0.958	0.962	

Table S8: **Edges for the network in CD4+ cells.** The interactions are ordered by increasing q-value, where the q-value is the p-value of the partial correlation coefficient corrected for multiple testing according to Benjamini-Hochberg. The first two colums give the variables that are connected, the Cor column gives the correlation coefficient, the PCor column gives the partial correlation coefficient (the strength of the edge), and the p-value (respectively q-value) column gives the p-value (respectively q-value) of this coefficient. The last column indicates, as a result of the cross-validation, whether the edge should be drawn in the network.

7.2 Network for IMR90 cells

variable 1	variable 2	Cor	PCor	p-value	q-value	draw
H2AK5ac	H2BK12ac	0.93	0.34	0	0	✓
H2BK120ac	H2BK20ac	0.97	0.4	0	0	✓
H3K14ac	H3K23ac	0.98	0.55	0	0	✓
H2AK5ac	H3K4ac	0.96	0.44	0	0	✓
H3K4me1	H3K4me2	0.68	0.38	0	0	✓
H3K79me1	H3K79me2	0.92	0.33	0	0	✓
H3K4me3	H3K9ac	0.96	0.49	0	0	✓
H3K4ac	H4K91ac	0.99	0.36	0	0	✓
H2BK12ac	H3K18ac	0.95	0.29	1.1e-250	1.7e-249	✓
H3K18ac	H4K5ac	0.95	0.28	3.8e-238	5.0e-237	✓
H3K4me1	H3K4me3	0.53	-0.28	3.6e-226	4.3e-225	✓
H3K14ac	H3K18ac	0.97	0.26	1.8e-198	2.0e-197	✓
H4K20me1	H4K5ac	0.66	-0.25	7.3e-190	7.4e-189	✓
H3K27ac	H3K27me3	-0.45	-0.25	3.0e-182	2.9e-181	✓
mRNA	H3K79me2	0.75	0.24	5.8e-174	5.1e-173	✓
DNaseIHS	H3K27ac	0.87	0.23	4.5e-149	3.7e-148	✓
H2BK12ac	H4K5ac	0.9	-0.22	2.0e-141	1.5e-140	✓
H2AK5ac	H2BK120ac	0.92	-0.21	1.3e-131	9.5e-131	✓
H3K4ac	H4K5ac	0.97	0.2	3.2e-121	2.2e-120	✓
H3K23ac	H4K20me1	0.76	0.2	1.1e-113	7.0e-113	✓
H3K27me3	H3K79me1	-0.45	-0.19	3.8e-107	2.4e-106	✓
H2AK5ac	H4K20me1	0.75	0.19	3.1e-104	1.9e-103	
H3K36me3	H3K4me1	0.67	0.19	4.4e-103	2.5e-102	
H2BK120ac	H4K91ac	0.96	0.17	1.3e-88	6.8e-88	
H3K27me3	H3K9ac	-0.41	0.17	7.7e-86	4.0e-85	
H3K4me2	H3K9ac	0.94	0.17	9.4e-80	4.6e-79	
H3K18ac	H3K79me1	0.85	-0.16	1.4e-76	6.6e-76	
H2BK20ac	H3K27ac	0.86	-0.16	1.5e-70	6.9e-70	
H3K36me3	H3K79me1	0.66	0.15	5.3e-69	2.3e-68	
H3K9ac	H3K9me3	-0.18	-0.15	2.2e-64	9.4e-64	
H2AK5ac	H3K79me2	0.89	0.14	1.9e-57	7.9e-57	
H2AK5ac	H3K36me3	0.7	0.14	1.7e-56	6.9e-56	
DNaseIHS	H3K18ac	0.83	-0.14	2.4e-53	9.2e-53	
H2BK120ac	H3K4me3	0.8	0.13	6.1e-49	2.3e-48	
DNaseIHS	H3K36me3	0.52	-0.12	1.2e-43	4.3e-43	
H2BK20ac	H4K5ac	0.88	0.12	6.1e-43	2.2e-42	
H3K4me2	H3K79me1	0.87	0.12	9.4e-41	3.3e-40	
H3K4me3	H3K9me3	-0.15	0.12	1.4e-39	4.7e-39	
H2BK120ac	H3K4me1	0.72	0.11	1.3e-38	4.3e-38	
H3K14ac	H3K79me2	0.9	0.11	3.1e-37	9.9e-37	
H3K79me2	H4K91ac	0.88	0.11	9.5e-37	3.0e-36	
H3K18ac	H3K79me2	0.85	-0.11	9.2e-36	2.8e-35	
mRNA	H3K27me3	-0.46	-0.11	3.0e-34	9.0e-34	✓
H3K4me1	H4K5ac	0.67	0.11	2.3e-33	6.8e-33	
H2AK5ac	H3K14ac	0.94	0.11	8.7e-33	2.5e-32	
H3K79me1	H3K9ac	0.88	-0.1	1.8e-31	5.0e-31	
mRNA	H3K9me3	-0.26	-0.1	2.4e-29	6.6e-29	✓
H3K23ac	H3K27me3	-0.35	0.1	3.0e-28	8.1e-28	
mRNA	H3K4me3	0.71	0.09	3.0e-26	7.8e-26	✓
H3K14ac	H3K9ac	0.94	0.09	1.1e-25	2.7e-25	
H3K36me3	H4K5ac	0.6	0.09	3.2e-23	8.1e-23	
DNaseIHS	H3K4me2	0.85	0.09	3.3e-22	8.0e-22	
H2BK120ac	H3K4me2	0.85	-0.08	7.0e-22	1.7e-21	
DNaseIHS	H2BK12ac	0.78	0.08	1.9e-21	4.5e-21	
H2BK120ac	H3K14ac	0.95	-0.08	5.1e-20	1.2e-19	
DNaseIHS	H3K23ac	0.84	-0.08	1.3e-19	3.0e-19	
H2BK120ac	H4K20me1	0.65	-0.08	1.9e-18	4.3e-18	
H3K18ac	H3K4ac	0.97	-0.08	9.8e-18	2.2e-17	
H2BK20ac	H3K14ac	0.92	0.08	1.5e-17	3.3e-17	
H3K4me2	H3K9me3	-0.13	0.07	3.7e-17	7.8e-17	
DNaseIHS	H3K9ac	0.87	-0.07	3.5e-16	7.4e-16	
H3K23ac	H3K4me3	0.89	0.07	1.1e-15	2.3e-15	
H2BK12ac	H3K9me3	-0.16	-0.07	1.4e-14	2.9e-14	
H3K9me3	H4K91ac	-0.18	0.07	5.9e-14	1.2e-13	
H2BK120ac	H3K79me1	0.83	-0.07	8.7e-14	1.7e-13	
H2BK12ac	H3K9ac	0.85	-0.07	1.6e-13	3.1e-13	
H3K18ac	H4K91ac	0.97	0.06	8.9e-13	1.7e-12	
H3K9ac	H4K91ac	0.93	0.06	1.3e-12	2.5e-12	
H3K9ac	H4K20me1	0.76	-0.06	4.3e-12	8.0e-12	
H2AK5ac	H2BK20ac	0.9	-0.06	8.0e-12	1.5e-11	
H2AK5ac	H3K4me3	0.78	-0.06	1.8e-11	3.2e-11	
H2BK20ac	H3K23ac	0.9	0.06	4.5e-11	8.0e-11	

variable 1	variable 2	Cor	PCor	p-value	q-value	draw
H2BK120ac	H2BK12ac	0.98	0.38	0	0	✓
H2BK12ac	H2BK20ac	0.98	0.43	0	0	✓
H3K18ac	H3K27ac	0.96	0.36	0	0	✓
H2AK5ac	H3K4me1	0.79	0.35	0	0	✓
H3K4me2	H3K4me3	0.93	0.35	0	0	✓
H3K27ac	H3K9ac	0.98	0.51	0	0	✓
H3K27me3	H3K9me3	0.52	0.37	0	0	✓
H3K14ac	H3K4ac	0.98	0.31	2.6e-286	4.1e-285	✓
H3K36me3	H3K9me3	0.11	0.29	2.2e-245	3.0e-244	✓
H3K36me3	H4K20me1	0.71	0.28	3.9e-237	5.0e-236	✓
H3K14ac	H3K4me1	0.68	-0.27	2.8e-218	3.2e-217	✓
H3K79me2	H4K20me1	0.8	0.25	1.6e-190	1.6e-189	✓
H3K23ac	H3K4me2	0.93	0.25	8.4e-186	8.2e-185	✓
H3K79me1	H4K5ac	0.89	0.25	2.3e-178	2.1e-177	✓
H4K5ac	H4K91ac	0.97	0.24	1.1e-165	9.0e-165	✓
H2BK120ac	H3K4ac	0.96	0.22	1.9e-142	1.5e-141	✓
H3K27me3	H3K4me1	-0.07	0.22	7.2e-140	5.4e-139	✓
H3K27ac	H3K4ac	0.96	0.21	6.9e-123	4.8e-122	✓
DNaseIHS	H4K20me1	0.72	0.2	5.0e-118	3.3e-117	✓
H3K27ac	H4K20me1	0.74	0.2	2.0e-113	1.3e-112	✓
H3K4me1	H3K79me1	0.7	0.19	1.7e-104	1.0e-103	
DNaseIHS	H3K4me3	0.86	0.19	6.5e-104	3.7e-103	
H3K79me2	H3K9ac	0.92	0.18	1.3e-97	7.4e-97	
H3K14ac	H3K4me3	0.87	-0.17	2.3e-88	1.2e-87	
H2AK5ac	H3K18ac	0.91	-0.17	2.8e-82	1.4e-81	
H3K23ac	H4K5ac	0.93	-0.17	2.8e-79	1.3e-78	
H3K27me3	H4K20me1	-0.18	0.16	6.5e-72	3.0e-71	
H3K36me3	H3K4me2	0.56	-0.15	2.7e-69	1.2e-68	
H3K27me3	H3K79me2	-0.45	-0.15	3.7e-67	1.6e-66	
H3K79me1	H3K9me3	-0.22	-0.14	1.0e-57	4.4e-57	
H2AK5ac	H3K23ac	0.91	-0.14	6.4e-57	2.6e-56	
H3K14ac	H3K27ac	0.95	-0.14	8.6e-54	3.4e-53	
H3K14ac	H3K79me1	0.9	0.13	6.8e-50	2.6e-49	
H2AK5ac	H4K91ac	0.95	0.13	2.2e-46	8.3e-46	
H3K23ac	H3K79me1	0.9	0.12	2.7e-43	9.9e-43	
H3K23ac	H3K9ac	0.95	0.12	4.9e-41	1.7e-40	
H4K20me1	H4K91ac	0.68	-0.12	1.2e-40	4.1e-40	
H2BK120ac	H4K5ac	0.92	-0.12	2.1e-39	6.9e-39	
H3K4me2	H3K79me2	0.86	-0.11	1.5e-38	4.8e-38	
H2BK120ac	H3K18ac	0.97	0.11	4.2e-37	1.3e-36	
H3K4me3	H4K5ac	0.87	0.11	5.9e-36	1.8e-35	
H3K4me3	H3K79me2	0.87	0.11	1.5e-35	4.4e-35	
H2BK120ac	H3K23ac	0.93	0.11	6.5e-34	1.9e-33	
H3K4me3	H3K79me1	0.84	0.11	4.1e-33	1.2e-32	
H3K4me3	H4K91ac	0.84	-0.1	3.3e-32	9.3e-32	
H3K14ac	H4K5ac	0.96	0.1	1.4e-29	3.9e-29	
H2BK120ac	H3K27ac	0.92	0.1	1.8e-28	4.8e-28	
H2AK5ac	H3K4me2	0.84	-0.1	7.1e-28	1.9e-27	
H3K4me1	H4K91ac	0.7	-0.09	4.3e-26	1.1e-25	
H3K27ac	H3K4me2	0.91	-0.09	2.6e-25	6.7e-25	
H2AK5ac	H3K27ac	0.91	-0.09	2.7e-22	6.6e-22	
H3K14ac	H3K36me3	0.6	-0.09	4.1e-22	1.0e-21	
DNaseIHS	H3K4me1	0.6	0.08	1.3e-21	3.0e-21	
H3K36me3	H3K4me3	0.53	-0.08	3.3e-20	7.6e-20	
H3K36me3	H3K9ac	0.6	0.08	7.5e-20	1.7e-19	
DNaseIHS	H3K27me3	-0.39	-0.08	1.9e-18	4.3e-18	
H3K18ac	H3K4me2	0.89	0.08	7.7e-18	1.7e-17	
DNaseIHS	H4K5ac	0.83	0.08	1.3e-17	2.8e-17	
H2BK12ac	H3K4ac	0.95	-0.08	1.8e-17	3.9e-17	
H2BK20ac	H3K4ac	0.92	-0.07	1.6e-16	3.3e-16	
DNaseIHS	H2BK120ac	0.79	-0.07	5.8e-16	1.2e-15	
H3K4ac	H4K20me1	0.71	-0.07	4.7e-15	9.7e-15	
H3K79me2	H4K5ac	0.88	-0.07	1.5e-14	3.1e-14	
DNaseIHS	H2BK20ac	0.75	0.07	7.2e-14	1.4e-13	
DNaseIHS	H3K14ac	0.84	0.07	1.6e-13	3.1e-13	
H3K36me3	H3K79me2	0.67	0.06	3.5e-13	6.8e-13	
H2BK120ac	H3K9ac	0.88	-0.06	9.1e-13	1.7e-12	
H3K18ac	H3K23ac	0.95	-0.06	1.4e-12	2.7e-12	
H2BK20ac	H3K79me1	0.81	0.06	4.5e-12	8.2e-12	
H3K18ac	H3K9me3	-0.17	0.06	1.7e-11	3.0e-11	
mRNA	H3K4ac	0.7	0.06	2.3e-11	4.1e-11	
H3K23ac	H3K4ac	0.96	0.06	5.8e-11	1.0e-10	

H2BK12ac	H3K4me2	0.84	0.06	5.8e-11	1.0e-10
H2BK20ac	H3K9ac	0.82	-0.06	1.5e-10	2.5e-10
H2BK20ac	H3K4me3	0.73	-0.06	2.2e-10	3.8e-10
H3K27ac	H3K4me3	0.91	-0.05	5.8e-10	9.7e-10
H3K79me2	H3K9me3	-0.19	0.05	8.9e-09	1.5e-08
H3K23ac	H3K79me2	0.9	-0.05	1.0e-08	1.7e-08
H3K4me2	H4K91ac	0.87	-0.05	2.1e-08	3.3e-08
H3K27ac	H4K91ac	0.95	0.05	5.0e-08	8.0e-08
DNaseIHS	H3K9me3	-0.16	0.05	6.2e-08	9.8e-08
H3K4me1	H3K9me3	-0.01	-0.05	1.5e-07	2.3e-07
H2BK12ac	H3K79me1	0.83	0.05	2.1e-07	3.2e-07
H2BK12ac	H3K36me3	0.56	-0.04	6.1e-07	9.2e-07
H3K4me3	H4K20me1	0.73	0.04	1.2e-06	1.8e-06
H2BK120ac	H3K27me3	-0.34	0.04	1.9e-06	2.8e-06
H3K27me3	H3K4me2	-0.34	-0.04	5.0e-06	7.3e-06
H3K23ac	H3K9me3	-0.15	0.04	2.2e-05	3.1e-05
mRNA	H3K9ac	0.72	-0.04	3.8e-05	5.4e-05
H3K18ac	H3K4me1	0.68	0.04	5.2e-05	7.4e-05
H3K27me3	H3K4ac	-0.41	-0.04	6.5e-05	9.1e-05
DNaseIHS	H3K79me2	0.81	-0.03	9.1e-05	1.3e-04
H2BK20ac	H4K20me1	0.63	0.03	1.2e-04	1.6e-04
H3K9me3	H4K20me1	-0.04	-0.03	1.7e-04	2.4e-04
mRNA	H3K18ac	0.67	0.03	8.8e-04	0.001
H3K4ac	H3K4me2	0.89	0.03	0.001	0.001
H3K27me3	H3K36me3	-0.11	0.03	0.003	0.004
H2BK20ac	H3K9me3	-0.14	0.02	0.007	0.009
H3K4ac	H3K4me1	0.71	-0.02	0.009	0.012
H2BK120ac	H3K36me3	0.55	-0.02	0.01	0.013
H2AK5ac	H3K27me3	-0.35	0.02	0.014	0.017
mRNA	H3K27ac	0.72	0.02	0.02	0.025
mRNA	H4K20me1	0.57	-0.02	0.023	0.029
H3K14ac	H3K9me3	-0.19	-0.02	0.024	0.029
H3K4me1	H3K9ac	0.63	-0.02	0.031	0.037
H3K14ac	H4K20me1	0.72	-0.02	0.04	0.048
mRNA	H3K14ac	0.7	-0.02	0.043	0.051
H3K79me1	H4K20me1	0.74	-0.02	0.052	0.061
H2BK12ac	H3K14ac	0.94	-0.02	0.078	0.091
H2BK20ac	H3K4me2	0.81	-0.02	0.079	0.091
H2AK5ac	H3K79me1	0.89	0.01	0.099	0.113
H3K14ac	H4K91ac	0.97	-0.01	0.113	0.128
mRNA	H2BK12ac	0.62	-0.01	0.173	0.194
mRNA	H4K91ac	0.68	-0.01	0.2	0.223
H2BK12ac	H3K27me3	-0.32	-0.01	0.215	0.238
H3K27ac	H3K4me1	0.65	0.01	0.24	0.263
mRNA	H2BK120ac	0.64	0.01	0.279	0.303
H2BK12ac	H4K20me1	0.66	-0.01	0.302	0.325
mRNA	H2BK20ac	0.59	-0.01	0.324	0.344
H2BK20ac	H3K4me1	0.72	0.01	0.407	0.431
H2BK12ac	H3K27ac	0.89	-0.01	0.44	0.464
H3K27me3	H4K5ac	-0.43	-0.01	0.552	0.575
H2BK20ac	H3K18ac	0.93	0	0.69	0.713
H3K4me2	H4K5ac	0.89	0	0.735	0.752
H3K36me3	H3K4ac	0.62	0	0.785	0.798
H3K18ac	H3K9ac	0.93	0	0.814	0.82
H2BK120ac	H3K79me2	0.82	0	0.877	0.877
H3K4ac	H3K79me2	0.9	-0.06	1.4e-10	2.5e-10
H3K18ac	H3K36me3	0.55	-0.06	1.5e-10	2.5e-10
H2AK5ac	H3K9ac	0.88	0.06	2.5e-10	4.3e-10
H3K23ac	H3K27ac	0.94	-0.05	1.1e-09	1.9e-09
H3K27ac	H3K36me3	0.6	0.05	9.0e-09	1.5e-08
H3K4ac	H3K79me1	0.89	-0.05	2.0e-08	3.3e-08
H3K4me1	H4K20me1	0.65	0.05	4.8e-08	7.6e-08
mRNA	H2AK5ac	0.65	-0.05	6.2e-08	9.8e-08
mRNA	H4K5ac	0.68	-0.05	7.9e-08	1.2e-07
H3K18ac	H4K20me1	0.68	0.05	1.8e-07	2.8e-07
H3K4me1	H3K79me2	0.65	-0.05	3.0e-07	4.5e-07
H3K9ac	H4K5ac	0.94	0.04	6.2e-07	9.4e-07
H2BK12ac	H4K91ac	0.95	0.04	1.6e-06	2.3e-06
H3K23ac	H3K36me3	0.61	0.04	3.3e-06	4.9e-06
H3K18ac	H3K27me3	-0.38	0.04	1.1e-05	1.6e-05
H3K27ac	H3K79me2	0.9	-0.04	2.5e-05	3.6e-05
H3K27ac	H3K79me1	0.87	-0.04	3.9e-05	5.6e-05
H3K79me1	H4K91ac	0.88	-0.04	5.3e-05	7.5e-05
DNaseIHS	H2AK5ac	0.79	-0.03	9.1e-05	1.3e-04
H3K27me3	H3K4me3	-0.39	0.03	9.6e-05	1.3e-04
H2BK20ac	H3K27me3	-0.3	0.03	1.7e-04	2.3e-04
H3K23ac	H4K91ac	0.94	-0.03	3.2e-04	4.3e-04
H3K9me3	H4K5ac	-0.18	0.03	9.4e-04	0.001
H3K4me2	H4K20me1	0.75	0.03	0.001	0.002
H3K27me3	H4K91ac	-0.41	-0.02	0.006	0.008
H2AK5ac	H4K5ac	0.92	-0.02	0.007	0.009
H3K27ac	H4K5ac	0.95	0.02	0.009	0.012
DNaseIHS	H3K4ac	0.84	-0.02	0.011	0.014
H3K4ac	H3K9me3	-0.19	-0.02	0.017	0.022
H3K14ac	H3K27me3	-0.41	-0.02	0.022	0.027
H2BK12ac	H3K4me1	0.74	0.02	0.024	0.029
H2BK20ac	H4K91ac	0.93	0.02	0.025	0.03
H2BK12ac	H3K4me3	0.77	-0.02	0.039	0.047
H3K23ac	H3K4me1	0.69	0.02	0.042	0.05
H2BK20ac	H3K36me3	0.52	-0.02	0.049	0.058
mRNA	DNaseIHS	0.64	-0.02	0.071	0.083
H3K36me3	H4K91ac	0.6	0.02	0.079	0.091
mRNA	H3K36me3	0.44	0.01	0.096	0.11
mRNA	H3K4me1	0.44	0.01	0.102	0.116
H2BK12ac	H3K79me2	0.82	-0.01	0.171	0.193
H2AK5ac	H3K9me3	-0.16	-0.01	0.178	0.199
DNaseIHS	H4K91ac	0.82	0.01	0.203	0.225
DNaseIHS	H3K79me1	0.8	-0.01	0.228	0.25
mRNA	H3K4me2	0.68	0.01	0.268	0.292
mRNA	H3K23ac	0.69	-0.01	0.298	0.323
H3K4ac	H3K9ac	0.94	-0.01	0.307	0.329
H3K14ac	H3K4me2	0.91	-0.01	0.323	0.345
H3K27ac	H3K9me3	-0.2	-0.01	0.44	0.462
H3K4ac	H3K4me3	0.86	-0.01	0.518	0.541
H2BK20ac	H3K79me2	0.78	0.01	0.556	0.577
H3K18ac	H3K4me3	0.86	0	0.715	0.736
mRNA	H3K79me1	0.7	0	0.745	0.76
H2BK12ac	H3K23ac	0.92	0	0.795	0.804
H2BK120ac	H3K9me3	-0.16	0	0.834	0.837

Table S9: **Edges for the network in IMR90 cells.** The interactions are ordered by increasing q-value, where the q-value is the p-value of the partial correlation coefficient corrected for multiple testing according to Benjamini-Hochberg. The first two colums give the variables that are connected, the Cor column gives the correlation coefficient, the PCor column gives the partial correlation coefficient (the strength of the edge), and the p-value (respectively q-value) column gives the p-value (respectively q-value) of this coefficient. The last column indicates, as a result of the cross-validation, whether the edge should be drawn in the network.

7.3 Network for H1 cells

variable 1	variable 2	Cor	PCor	p-value	q-value	draw
H2BK12ac	H2BK20ac	0.86	0.35	0	0	✓
H3K4me2	H3K4me3	0.82	0.42	0	0	✓
H3K27ac	H3K9ac	0.92	0.37	0	0	✓
H3K36me3	H3K9me3	0.56	0.37	0	0	✓
H2AK5ac	H2BK12ac	0.85	0.3	8.2e-277	2.3e-275	✓
H3K79me2	H4K20me1	0.67	0.3	7.9e-267	1.8e-265	✓
H3K18ac	H4K5ac	0.77	0.25	2.9e-193	5.7e-192	✓
H3K36me3	H4K20me1	0.64	0.25	1.0e-187	1.7e-186	✓
H2BK120ac	H3K18ac	0.89	0.23	7.1e-164	1.1e-162	✓
H2BK120ac	H4K91ac	0.89	0.23	5.7e-158	7.6e-157	✓
H3K27me3	H3K9me3	0.61	0.22	4.4e-150	5.2e-149	✓
H3K27me3	H3K4me1	0.64	0.22	8.5e-143	9.3e-142	✓
H3K14ac	H3K18ac	0.86	0.22	1.0e-140	1.1e-139	✓
H3K4me2	H3K79me2	0.58	-0.2	1.2e-121	1.1e-120	✓
DNaseIHS	H3K4me3	0.7	0.2	2.6e-119	2.3e-118	✓
H3K27me3	H3K79me1	0.25	-0.18	1.1e-100	9.0e-100	✓
H2AK5ac	H4K91ac	0.86	0.18	7.9e-99	6.1e-98	✓
H2BK20ac	H3K27me3	0.54	0.18	8.0e-96	5.8e-95	✓
H3K36me3	H3K9ac	0.42	0.18	1.0e-90	7.0e-90	✓
H2BK120ac	H2BK20ac	0.8	0.17	1.1e-87	7.0e-87	✓
mRNA	H3K9me3	-0.01	-0.16	9.8e-80	6.0e-79	✓
DNaseIHS	H4K20me1	0.62	0.16	3.5e-76	2.0e-75	✓
DNaseIHS	H2BK20ac	0.28	-0.16	7.0e-75	3.9e-74	✓
mRNA	H3K79me1	0.64	0.15	3.7e-69	2.0e-68	✓
H3K4ac	H4K91ac	0.89	0.15	8.2e-64	4.2e-63	
H2AK5ac	H3K4me3	0.46	-0.15	1.1e-62	5.5e-62	
H3K27me3	H4K20me1	0.54	0.14	1.0e-61	5.0e-61	
H4K20me1	H4K5ac	0.31	-0.14	6.2e-59	2.9e-58	
H3K18ac	H3K79me1	0.68	-0.14	8.9e-57	4.0e-56	
H3K9ac	H4K20me1	0.46	-0.13	1.3e-53	5.5e-53	
H3K4me3	H4K5ac	0.83	0.13	3.3e-52	1.4e-51	
H3K18ac	H3K9ac	0.71	-0.13	2.6e-51	1.0e-50	
H2BK12ac	H4K91ac	0.86	0.13	6.5e-49	2.5e-48	
H3K18ac	H3K27me3	0.45	0.13	3.3e-47	1.3e-46	
H3K4me3	H3K79me1	0.75	0.12	6.6e-44	2.4e-43	
DNaseIHS	H3K9me3	0.36	0.12	1.2e-43	4.3e-43	
DNaseIHS	H4K5ac	0.53	-0.12	2.6e-41	9.1e-41	
H2AK5ac	H3K79me2	0.71	0.12	7.4e-41	2.5e-40	
H3K4me2	H4K20me1	0.6	0.11	1.6e-39	5.3e-39	
H2BK20ac	H3K23ac	0.73	0.11	1.1e-38	3.6e-38	
H2AK5ac	H4K20me1	0.55	0.11	5.7e-38	1.8e-37	
H3K4me1	H3K79me2	0.5	-0.1	4.8e-33	1.5e-32	
H2AK5ac	H3K18ac	0.76	-0.1	1.1e-30	3.4e-30	
H2BK20ac	H3K4ac	0.75	0.1	3.2e-29	9.3e-29	
H3K9ac	H3K9me3	0.09	-0.1	8.6e-28	2.4e-27	
H3K14ac	H4K5ac	0.64	-0.09	2.6e-26	7.3e-26	
H3K79me1	H4K91ac	0.71	0.09	5.8e-26	1.6e-25	
H3K4me1	H4K5ac	0.45	0.09	3.0e-23	7.9e-23	
H3K36me3	H3K4me1	0.56	0.08	5.3e-22	1.4e-21	
mRNA	H3K23ac	0.37	-0.08	3.5e-21	8.8e-21	✓
H2AK5ac	H3K23ac	0.79	0.08	5.8e-21	1.5e-20	
H2BK120ac	H3K4ac	0.87	0.08	6.4e-20	1.6e-19	
H2AK5ac	H3K79me1	0.71	0.08	1.7e-19	4.0e-19	
H3K4ac	H3K79me1	0.76	0.08	4.2e-18	9.8e-18	
mRNA	DNaseIHS	0.49	0.08	1.0e-17	2.4e-17	
H2BK12ac	H3K4me1	0.79	0.07	3.4e-17	7.7e-17	
H3K4me2	H4K5ac	0.69	0.07	5.7e-17	1.3e-16	
H3K14ac	H3K79me2	0.7	0.07	2.0e-16	4.4e-16	
H3K9me3	H4K91ac	0.38	0.07	2.4e-15	5.2e-15	
H3K79me2	H3K9me3	0.31	0.07	9.1e-15	1.9e-14	
H2BK12ac	H3K36me3	0.5	-0.06	1.2e-13	2.4e-13	
H3K27me3	H3K4me2	0.49	0.06	1.7e-13	3.5e-13	
mRNA	H2BK12ac	0.33	-0.06	4.5e-13	9.2e-13	
H3K36me3	H3K4ac	0.54	0.06	1.1e-12	2.1e-12	
H3K4me3	H4K91ac	0.57	-0.06	1.5e-12	2.9e-12	
H3K4me3	H4K20me1	0.5	0.06	1.1e-11	2.1e-11	
H3K4ac	H3K79me2	0.74	0.06	8.8e-11	1.7e-10	
mRNA	H4K91ac	0.48	0.06	1.2e-10	2.2e-10	
DNaseIHS	H2BK12ac	0.46	-0.05	4.1e-10	7.6e-10	
DNaseIHS	H3K14ac	0.53	-0.05	6.9e-10	1.3e-09	
H3K18ac	H3K79me2	0.63	-0.05	1.1e-09	2.0e-09	
H2BK12ac	H3K79me2	0.57	-0.05	1.6e-09	2.8e-09	

variable 1	variable 2	Cor	PCor	p-value	q-value	draw
H3K14ac	H3K23ac	0.89	0.4	0	0	✓
H3K79me1	H3K79me2	0.88	0.39	0	0	✓
H3K4me3	H3K9ac	0.94	0.53	0	0	✓
H3K27ac	H4K5ac	0.97	0.7	0	0	✓
H3K4me1	H3K4me3	0.41	-0.3	7.0e-267	1.8e-265	✓
H3K4me1	H3K4me2	0.67	0.3	4.2e-264	8.9e-263	✓
DNaseIHS	H3K27me3	0.49	0.25	1.1e-191	1.9e-190	✓
H3K14ac	H3K4ac	0.9	0.24	5.5e-170	8.6e-169	✓
H3K18ac	H4K91ac	0.87	0.23	3.4e-160	4.8e-159	✓
H2AK5ac	H3K4ac	0.87	0.23	8.7e-157	1.1e-155	✓
H3K4me1	H3K79me1	0.61	0.22	1.4e-148	1.6e-147	✓
H3K79me1	H4K20me1	0.7	0.22	2.7e-141	2.8e-140	✓
H3K23ac	H3K4ac	0.85	0.22	8.5e-139	8.3e-138	✓
H2BK12ac	H3K18ac	0.85	0.2	1.3e-119	1.2e-118	✓
H3K27ac	H3K27me3	0.02	-0.19	4.3e-101	3.6e-100	✓
DNaseIHS	H3K4me1	0.57	0.18	6.0e-100	4.8e-99	✓
H3K27ac	H3K4me2	0.69	-0.18	1.1e-98	8.3e-98	✓
H3K18ac	H3K4me2	0.79	0.18	6.7e-93	4.7e-92	✓
H2AK5ac	H3K4me2	0.53	-0.17	2.6e-90	1.7e-89	✓
H3K4ac	H4K20me1	0.52	-0.17	3.6e-85	2.3e-84	✓
H2BK20ac	H3K9ac	0.29	-0.16	6.2e-77	3.8e-76	✓
H3K4me3	H3K79me2	0.71	0.16	6.0e-75	3.5e-74	✓
H4K20me1	H4K91ac	0.47	-0.15	4.0e-70	2.2e-69	✓
H3K27me3	H3K4me3	0.19	0.15	1.6e-65	8.6e-65	✓
H3K27me3	H4K5ac	0	-0.15	4.8e-63	2.4e-62	
H3K4me1	H4K20me1	0.63	0.15	2.0e-62	9.7e-62	
DNaseIHS	H3K27ac	0.57	0.14	4.6e-59	2.2e-58	
H3K23ac	H4K20me1	0.64	0.14	3.3e-58	1.5e-57	
H2BK12ac	H3K9ac	0.52	0.14	1.2e-56	5.1e-56	
H3K36me3	H3K4me3	0.4	-0.13	1.7e-53	7.1e-53	
H2AK5ac	H3K9ac	0.55	0.13	6.0e-52	2.5e-51	
mRNA	H3K36me3	0.36	0.13	3.9e-49	1.5e-48	✓
H3K79me2	H4K91ac	0.7	0.13	1.4e-47	5.2e-47	
H2BK120ac	H2BK12ac	0.88	0.13	1.0e-46	3.9e-46	
H2BK20ac	H3K4me1	0.74	0.12	7.1e-44	2.6e-43	
DNaseIHS	H4K91ac	0.52	0.12	2.0e-43	7.2e-43	
H3K23ac	H3K4me2	0.71	0.12	4.2e-41	1.4e-40	
H3K4me2	H3K9ac	0.77	0.12	2.1e-40	7.1e-40	
DNaseIHS	H3K79me2	0.53	-0.11	7.4e-39	2.4e-38	
H2BK12ac	H3K4me2	0.65	0.11	5.4e-38	1.7e-37	
H3K23ac	H4K91ac	0.76	-0.11	1.0e-37	3.1e-37	
H3K79me2	H3K9ac	0.74	0.1	5.5e-33	1.7e-32	
H3K4me2	H4K91ac	0.62	-0.1	2.6e-29	7.7e-29	
H3K14ac	H3K4me2	0.73	0.1	4.3e-28	1.2e-27	
DNaseIHS	H3K23ac	0.5	-0.09	5.5e-27	1.5e-26	
H3K14ac	H3K36me3	0.52	-0.09	4.0e-26	1.1e-25	
H3K27ac	H4K20me1	0.37	0.09	7.6e-26	2.0e-25	
DNaseIHS	H3K79me1	0.63	0.09	5.5e-23	1.4e-22	
H3K27ac	H3K36me3	0.37	-0.08	5.3e-22	1.4e-21	
H3K9me3	H4K5ac	0	-0.08	4.3e-21	1.1e-20	
mRNA	H3K4me3	0.66	0.08	8.5e-21	2.1e-20	
H3K14ac	H3K27ac	0.67	0.08	1.5e-19	3.8e-19	
H3K4me1	H3K9me3	0.48	-0.08	3.5e-18	8.5e-18	
DNaseIHS	H3K18ac	0.61	0.08	4.9e-18	1.2e-17	
H3K36me3	H4K5ac	0.34	0.07	2.1e-17	4.8e-17	
H3K18ac	H3K4me1	0.75	0.07	4.7e-17	1.1e-16	
H2BK12ac	H3K4me3	0.47	-0.07	1.5e-16	3.3e-16	
H3K14ac	H4K91ac	0.84	0.07	1.7e-15	3.7e-15	
mRNA	H3K4me1	0.32	0.07	4.6e-15	9.9e-15	
H2BK120ac	H3K9ac	0.58	-0.07	3.2e-14	6.8e-14	
H3K4me1	H3K9ac	0.42	-0.06	1.7e-13	3.4e-13	
H3K27me3	H3K79me2	0.2	-0.06	3.2e-13	6.6e-13	
H3K27me3	H3K9ac	0.12	0.06	1.0e-12	2.1e-12	
H3K27ac	H4K91ac	0.71	0.06	1.4e-12	2.7e-12	
H3K18ac	H3K4ac	0.87	0.06	8.6e-12	1.7e-11	
H2AK5ac	H3K4me1	0.71	0.06	6.3e-11	1.2e-10	
mRNA	H4K20me1	0.35	-0.06	1.1e-10	2.0e-10	
mRNA	H2BK20ac	0.16	-0.05	3.7e-10	6.9e-10	
H3K23ac	H3K9ac	0.59	0.05	4.1e-10	7.7e-10	
H3K23ac	H4K5ac	0.55	-0.05	8.6e-10	1.5e-09	
H2BK120ac	H3K4me1	0.76	0.05	1.3e-09	2.3e-09	
H2BK120ac	H3K4me3	0.54	0.05	2.5e-09	4.4e-09	

mRNA	H2AK5ac	0.42	0.05	3.6e-09	6.3e-09	
mRNA	H3K79me2	0.6	0.05	1.2e-08	2.1e-08	
H3K79me1	H3K9me3	0.32	0.05	1.3e-08	2.3e-08	
H2BK20ac	H4K20me1	0.41	-0.05	2.9e-08	4.9e-08	
H3K18ac	H3K36me3	0.49	0.05	3.8e-08	6.3e-08	
H3K18ac	H3K4me3	0.69	0.05	1.2e-07	2.0e-07	
H3K14ac	H3K9ac	0.64	-0.05	2.5e-07	4.0e-07	
H3K23ac	H3K79me1	0.71	0.04	6.1e-07	9.7e-07	
H2BK12ac	H3K14ac	0.83	-0.04	6.3e-07	1.0e-06	
H3K27ac	H3K4ac	0.74	0.04	1.2e-06	1.8e-06	
H3K27ac	H3K79me2	0.7	-0.04	2.2e-06	3.4e-06	
H2BK12ac	H3K79me1	0.61	-0.04	2.8e-06	4.2e-06	
H2BK20ac	H4K91ac	0.76	0.04	4.2e-06	6.3e-06	
H2BK20ac	H3K18ac	0.72	-0.04	4.6e-06	6.8e-06	
H2BK20ac	H3K4me3	0.24	-0.04	1.3e-05	1.9e-05	
H2BK120ac	H3K36me3	0.5	0.04	1.6e-05	2.3e-05	
mRNA	H3K27ac	0.68	0.04	3.3e-05	4.7e-05	
H3K36me3	H3K4me2	0.47	-0.04	4.4e-05	6.2e-05	
mRNA	H3K4ac	0.5	0.04	4.7e-05	6.6e-05	
H3K36me3	H3K79me2	0.56	0.04	6.1e-05	8.4e-05	
H3K27ac	H3K4me1	0.46	0.03	9.6e-05	1.3e-04	
H2BK120ac	H3K79me2	0.62	0.03	2.6e-04	3.5e-04	
H2AK5ac	H2BK120ac	0.82	0.03	5.0e-04	6.7e-04	
H3K14ac	H3K79me1	0.73	0.03	6.9e-04	9.2e-04	
DNaseIHS	H3K36me3	0.47	-0.03	0.001	0.002	
H3K14ac	H3K27me3	0.46	0.03	0.002	0.003	
H3K9me3	H4K20me1	0.5	-0.03	0.003	0.004	
H2BK12ac	H4K5ac	0.56	-0.02	0.007	0.008	
H2AK5ac	H3K27me3	0.38	-0.02	0.01	0.013	
DNaseIHS	H2BK120ac	0.51	0.02	0.016	0.02	
H2BK20ac	H3K36me3	0.43	-0.02	0.021	0.026	
H3K4me2	H3K9me3	0.31	0.02	0.026	0.032	
H2BK12ac	H4K20me1	0.5	0.02	0.046	0.056	
H2BK120ac	H3K27ac	0.65	0.02	0.061	0.073	
H3K36me3	H3K79me1	0.58	0.02	0.077	0.091	
mRNA	H2BK120ac	0.41	-0.01	0.088	0.104	
DNaseIHS	H3K9ac	0.64	-0.01	0.108	0.127	
H3K4me2	H3K79me1	0.67	0.01	0.112	0.129	
mRNA	H4K5ac	0.66	0.01	0.134	0.153	
H2BK20ac	H3K79me2	0.43	0.01	0.166	0.188	
H2BK120ac	H3K4me2	0.66	-0.01	0.196	0.221	
H3K27ac	H3K79me1	0.73	0.01	0.245	0.273	
H3K27me3	H4K91ac	0.38	0.01	0.259	0.286	
H3K79me1	H4K5ac	0.69	0.01	0.272	0.298	
H3K23ac	H3K9me3	0.44	-0.01	0.304	0.33	
H3K4ac	H3K4me2	0.69	0.01	0.353	0.38	
H2BK20ac	H3K79me1	0.46	-0.01	0.47	0.501	
H3K79me1	H3K9ac	0.76	-0.01	0.503	0.53	
H3K4ac	H3K9me3	0.36	-0.01	0.516	0.542	
H2BK120ac	H4K5ac	0.64	-0.01	0.561	0.584	
H3K4ac	H3K4me3	0.63	0	0.637	0.658	
H3K23ac	H3K4me3	0.57	0	0.684	0.704	
DNaseIHS	H3K4me2	0.71	0	0.826	0.839	
H2AK5ac	H3K27ac	0.6	0	0.925	0.933	
H2BK120ac	H3K23ac	0.78	0	0.96	0.963	
H3K14ac	H3K4me1	0.73	-0.05	9.0e-09	1.6e-08	
H3K18ac	H3K27ac	0.77	0.05	1.3e-08	2.3e-08	
H2BK20ac	H4K5ac	0.37	0.05	2.8e-08	4.8e-08	
DNaseIHS	H3K4ac	0.51	-0.05	3.7e-08	6.2e-08	
H2BK12ac	H3K27me3	0.53	0.05	3.9e-08	6.5e-08	
H4K5ac	H4K91ac	0.68	-0.05	1.5e-07	2.4e-07	
H2AK5ac	H2BK20ac	0.77	0.04	3.4e-07	5.4e-07	
H3K4me1	H4K91ac	0.71	-0.04	6.4e-07	1.0e-06	
H3K4ac	H3K4me1	0.72	0.04	6.6e-07	1.0e-06	
H2AK5ac	H3K9me3	0.43	0.04	1.7e-06	2.7e-06	
H3K14ac	H4K20me1	0.59	0.04	2.2e-06	3.4e-06	
H2BK20ac	H3K27ac	0.38	0.04	2.9e-06	4.3e-06	
H2BK120ac	H3K79me1	0.65	-0.04	4.4e-06	6.5e-06	
H3K27me3	H3K4ac	0.37	-0.04	9.0e-06	1.3e-05	
H3K4ac	H3K9ac	0.69	0.04	1.4e-05	2.1e-05	
H3K36me3	H4K91ac	0.5	-0.04	1.9e-05	2.7e-05	
H2BK12ac	H3K27ac	0.57	-0.04	3.7e-05	5.3e-05	
mRNA	H3K27me3	-0.01	-0.04	4.6e-05	6.5e-05	
DNaseIHS	H2AK5ac	0.42	-0.04	5.4e-05	7.4e-05	
H3K14ac	H3K9me3	0.41	0.03	8.9e-05	1.2e-04	
H3K23ac	H3K27me3	0.5	0.03	1.5e-04	2.0e-04	
mRNA	H3K14ac	0.43	-0.03	2.7e-04	3.7e-04	
H2BK12ac	H3K9me3	0.45	0.03	6.0e-04	8.0e-04	
H3K23ac	H3K36me3	0.55	0.03	9.0e-04	0.001	
H2BK20ac	H3K14ac	0.74	0.03	0.002	0.002	
H2AK5ac	H3K36me3	0.56	0.03	0.002	0.003	
H3K9ac	H4K5ac	0.89	-0.03	0.004	0.005	
H2BK120ac	H4K20me1	0.49	-0.02	0.008	0.011	
H3K4me3	H3K9me3	0.12	0.02	0.013	0.016	
H3K18ac	H3K23ac	0.78	-0.02	0.02	0.024	
H2BK20ac	H3K4me2	0.47	-0.02	0.023	0.029	
H2BK120ac	H3K14ac	0.84	0.02	0.04	0.049	
H3K18ac	H4K20me1	0.5	-0.02	0.059	0.071	
H3K27ac	H3K9me3	0.04	0.02	0.067	0.08	
H2BK20ac	H3K9me3	0.46	0.02	0.077	0.091	
mRNA	H3K18ac	0.49	0.01	0.108	0.126	
H2BK120ac	H3K27me3	0.46	-0.01	0.11	0.127	
H3K9ac	H4K91ac	0.63	0.01	0.122	0.14	
H2AK5ac	H3K14ac	0.81	0.01	0.14	0.16	
H3K23ac	H3K27ac	0.58	-0.01	0.186	0.21	
H3K18ac	H3K9me3	0.34	-0.01	0.205	0.229	
H3K23ac	H3K4me1	0.73	0.01	0.252	0.279	
mRNA	H3K4me2	0.49	-0.01	0.266	0.293	
H3K4ac	H4K5ac	0.72	0.01	0.283	0.309	
H3K27ac	H3K4me3	0.85	-0.01	0.32	0.346	
H2BK12ac	H3K4ac	0.85	-0.01	0.416	0.446	
H3K27me3	H3K36me3	0.42	0.01	0.488	0.519	
H2BK12ac	H3K23ac	0.8	0.01	0.502	0.531	
H2BK120ac	H3K9me3	0.4	-0.01	0.554	0.579	
H3K79me2	H4K5ac	0.65	0	0.612	0.634	
mRNA	H3K9ac	0.68	0	0.685	0.702	
H3K23ac	H3K79me2	0.67	0	0.738	0.753	
H2AK5ac	H4K5ac	0.56	0	0.867	0.877	
H3K14ac	H3K4me3	0.62	0	0.962	0.962	

Table S10: **Edges for the network in H1 cells.** The interactions are ordered by increasing q-value, where the q-value is the p-value of the partial correlation coefficient corrected for multiple testing according to Benjamini-Hochberg. The first two colums give the variables that are connected, the Cor column gives the correlation coefficient, the PCor column gives the partial correlation coefficient (the strength of the edge), and the p-value (respectively q-value) column gives the p-value (respectively q-value) of this coefficient. The last column indicates, as a result of the cross-validation, whether the edge should be drawn in the network.

8 Effect matrices

Effect matrices here are presented as a list of pairs of variables with their properties.

8.1 Effect matrix for CD4+ cells

variable 1	variable 2	Cor	PCor	PCor- Cor	MIV
H2BK20ac	H3K9ac	0.884	-6.54e-02	-9.50e-01	H2BK120ac
H2BK20ac	H3K27ac	0.907	-1.44e-03	-9.08e-01	H2BK120ac
H3K4me3	H4K5ac	0.795	-9.25e-02	-8.87e-01	H3K27ac
H2BK12ac	H3K4me3	0.794	-7.49e-02	-8.69e-01	H2BK120ac
H2BK12ac	H3K18ac	0.927	0.065	-8.62e-01	H2BK120ac
H2BK12ac	H3K9ac	0.885	0.029	-8.56e-01	H2BK120ac
DNaseIHS	H3K18ac	0.863	0.008	-8.55e-01	H2BK120ac
H2BK20ac	H4K5ac	0.914	0.06	-8.54e-01	H2BK120ac
H3K4me2	H3K9ac	0.817	-3.53e-02	-8.52e-01	H3K4me3
H3K4ac	H3K4me3	0.808	-3.71e-02	-8.45e-01	H3K18ac
H3K27ac	H3K4ac	0.918	0.076	-8.42e-01	H2BK120ac
H4K5ac	H4K91ac	0.901	0.062	-8.39e-01	H2BK120ac
H2BK120ac	H3K27ac	0.931	0.099	-8.32e-01	H3K18ac
H2BK120ac	H4K5ac	0.922	0.094	-8.28e-01	H3K18ac
H2BK120ac	H3K4me2	0.824	7.61e-04	-8.24e-01	H3K18ac
H2BK120ac	H3K9ac	0.914	0.092	-8.23e-01	H3K18ac
H3K4me1	H3K4me3	0.676	-1.40e-01	-8.15e-01	H3K4me2
H2BK20ac	H3K4me3	0.809	-3.60e-03	-8.13e-01	H3K18ac
H3K4ac	H3K9ac	0.901	0.094	-8.06e-01	H2BK120ac
H3K18ac	H4K5ac	0.923	0.121	-8.01e-01	H2BK120ac
H3K18ac	H3K79me1	0.731	-6.86e-02	-7.99e-01	H4K91ac
H2BK120ac	H3K18ac	0.959	0.166	-7.93e-01	H2BK20ac
DNaseIHS	H4K5ac	0.806	0.02	-7.86e-01	H2BK120ac
H4K20me1	H4K5ac	0.499	-2.84e-01	-7.83e-01	H4K91ac
DNaseIHS	H2BK20ac	0.855	0.073	-7.82e-01	H2BK120ac
H2BK20ac	H3K4me2	0.805	0.029	-7.76e-01	H3K18ac
H3K4me1	H3K79me2	0.616	-1.59e-01	-7.75e-01	H3K79me1
H3K4me1	H3K9ac	0.664	-1.05e-01	-7.69e-01	H3K18ac
H3K18ac	H3K79me2	0.718	-4.99e-02	-7.68e-01	H3K4ac
H3K27ac	H4K5ac	0.913	0.149	-7.64e-01	H2BK120ac
H2BK20ac	H3K18ac	0.954	0.194	-7.60e-01	H2BK120ac
H3K79me2	H4K91ac	0.722	-2.94e-02	-7.51e-01	H3K4ac
H2BK120ac	H2BK20ac	0.956	0.209	-7.47e-01	H3K18ac
H3K4me1	H4K91ac	0.744	0.005	-7.39e-01	H3K18ac
H2BK20ac	H3K79me1	0.731	-5.38e-03	-7.37e-01	H4K91ac
H2AK5ac	H2BK120ac	0.713	-1.78e-02	-7.31e-01	H2BK20ac
H3K4ac	H3K79me1	0.749	0.021	-7.28e-01	H4K91ac
H3K4ac	H3K4me1	0.728	0.003	-7.25e-01	H4K91ac
H2BK12ac	H3K79me1	0.7	-2.38e-02	-7.24e-01	H3K4ac
H2BK20ac	H3K4me1	0.734	0.015	-7.19e-01	H4K91ac
H3K18ac	H3K4me3	0.85	0.133	-7.17e-01	H2BK120ac
H2AK5ac	H3K18ac	0.716	0.002	-7.14e-01	H2BK20ac
H3K4me2	H3K79me2	0.708	5.32e-04	-7.08e-01	H3K27ac
H2AK5ac	H3K9ac	0.627	-7.23e-02	-7.00e-01	H3K4ac
H3K27ac	H3K79me2	0.754	0.057	-6.97e-01	H3K9ac
H3K27ac	H3K4me1	0.691	-4.79e-03	-6.96e-01	H3K18ac
H3K4me3	H3K79me1	0.693	0.003	-6.90e-01	H3K4me2
H2BK20ac	H3K27me3	-6.02e-01	0.087	0.689	H4K91ac
H2AK5ac	H4K91ac	0.743	0.058	-6.85e-01	H2BK20ac
H3K79me2	H3K9ac	0.751	0.067	-6.83e-01	H3K27ac
mRNA	H3K18ac	0.614	-6.38e-02	-6.78e-01	H3K27ac
H3K27ac	H3K4me3	0.863	0.191	-6.73e-01	H3K9ac
H3K4ac	H3K79me2	0.74	0.069	-6.71e-01	H3K27ac
H3K4me3	H3K79me2	0.712	0.047	-6.65e-01	H3K9ac
H3K27ac	H4K20me1	0.568	-9.65e-02	-6.65e-01	H4K91ac
H2BK20ac	H3K23ac	0.644	-1.38e-02	-6.58e-01	H4K5ac
H2AK5ac	H3K4ac	0.748	0.095	-6.53e-01	H2BK20ac
H3K4me2	H4K20me1	0.664	0.017	-6.47e-01	H3K4me1
H3K23ac	H3K27ac	0.689	0.049	-6.41e-01	H4K5ac
H3K18ac	H3K23ac	0.673	0.035	-6.39e-01	H4K5ac
H2AK5ac	H2BK12ac	0.73	0.096	-6.34e-01	H2BK20ac
mRNA	DNaseIHS	0.633	4.64e-04	-6.32e-01	H3K27ac
H3K23ac	H3K4me3	0.678	0.051	-6.26e-01	H3K9ac
H3K23ac	H3K9ac	0.697	0.073	-6.25e-01	H4K5ac
H3K4me3	H3K9ac	0.876	0.255	-6.21e-01	H3K27ac
H3K4me1	H4K5ac	0.678	0.059	-6.19e-01	H3K18ac

variable 1	variable 2	Cor	PCor	PCor- Cor	MIV
H2BK20ac	H3K9ac	0.884	-6.54e-02	-9.50e-01	H2BK120ac
H2BK20ac	H3K27ac	0.907	-1.44e-03	-9.08e-01	H2BK120ac
H3K4me3	H4K5ac	0.795	-9.25e-02	-8.87e-01	H3K27ac
H2BK12ac	H3K4me3	0.794	-7.49e-02	-8.69e-01	H2BK120ac
H2BK12ac	H3K18ac	0.927	0.065	-8.62e-01	H2BK120ac
H2BK12ac	H3K9ac	0.885	0.029	-8.56e-01	H2BK120ac
DNaseIHS	H3K18ac	0.863	0.008	-8.55e-01	H2BK120ac
H2BK20ac	H4K5ac	0.914	0.06	-8.54e-01	H2BK120ac
H3K4me2	H3K9ac	0.817	-3.53e-02	-8.52e-01	H3K4me3
H3K4ac	H3K4me3	0.808	-3.71e-02	-8.45e-01	H3K18ac
H3K27ac	H3K4ac	0.918	0.076	-8.42e-01	H2BK120ac
H4K5ac	H4K91ac	0.901	0.062	-8.39e-01	H2BK120ac
H2BK120ac	H3K27ac	0.931	0.099	-8.32e-01	H3K18ac
H2BK120ac	H4K5ac	0.922	0.094	-8.28e-01	H3K18ac
H2BK120ac	H3K4me2	0.824	7.61e-04	-8.24e-01	H3K18ac
H2BK120ac	H3K9ac	0.914	0.092	-8.23e-01	H3K18ac
H3K4me1	H3K4me3	0.676	-1.40e-01	-8.15e-01	H3K4me2
H2BK20ac	H3K4me3	0.809	-3.60e-03	-8.13e-01	H3K18ac
H3K4ac	H3K9ac	0.901	0.094	-8.06e-01	H2BK120ac
H3K18ac	H4K5ac	0.923	0.121	-8.01e-01	H2BK120ac
H3K18ac	H3K79me1	0.731	-6.86e-02	-7.99e-01	H4K91ac
H2BK120ac	H3K18ac	0.959	0.166	-7.93e-01	H2BK20ac
DNaseIHS	H4K5ac	0.806	0.02	-7.86e-01	H2BK120ac
H4K20me1	H4K5ac	0.499	-2.84e-01	-7.83e-01	H4K91ac
DNaseIHS	H2BK20ac	0.855	0.073	-7.82e-01	H2BK120ac
H2BK20ac	H3K4me2	0.805	0.029	-7.76e-01	H3K18ac
H3K4me1	H3K79me2	0.616	-1.59e-01	-7.75e-01	H3K79me1
H3K4me1	H3K9ac	0.664	-1.05e-01	-7.69e-01	H3K18ac
H3K18ac	H3K79me2	0.718	-4.99e-02	-7.68e-01	H3K4ac
H3K27ac	H4K5ac	0.913	0.149	-7.64e-01	H2BK120ac
H2BK20ac	H3K18ac	0.954	0.194	-7.60e-01	H2BK120ac
H3K79me2	H4K91ac	0.722	-2.94e-02	-7.51e-01	H3K4ac
H2BK120ac	H2BK20ac	0.956	0.209	-7.47e-01	H3K18ac
H3K4me1	H4K91ac	0.744	0.005	-7.39e-01	H3K18ac
H2BK20ac	H3K79me1	0.731	-5.38e-03	-7.37e-01	H4K91ac
H2AK5ac	H2BK120ac	0.713	-1.78e-02	-7.31e-01	H2BK20ac
H3K4ac	H3K79me1	0.749	0.021	-7.28e-01	H4K91ac
H3K4ac	H3K4me1	0.728	0.003	-7.25e-01	H4K91ac
H2BK12ac	H3K79me1	0.7	-2.38e-02	-7.24e-01	H3K4ac
H2BK20ac	H3K4me1	0.734	0.015	-7.19e-01	H4K91ac
H3K18ac	H3K4me3	0.85	0.133	-7.17e-01	H2BK120ac
H2AK5ac	H3K18ac	0.716	0.002	-7.14e-01	H2BK20ac
H3K4me2	H3K79me2	0.708	5.32e-04	-7.08e-01	H3K27ac
H2AK5ac	H3K9ac	0.627	-7.23e-02	-7.00e-01	H3K4ac
H3K27ac	H3K79me2	0.754	0.057	-6.97e-01	H3K9ac
H3K27ac	H3K4me1	0.691	-4.79e-03	-6.96e-01	H3K18ac
H3K4me3	H3K79me1	0.693	0.003	-6.90e-01	H3K4me2
H2BK20ac	H3K27me3	-6.02e-01	0.087	0.689	H4K91ac
H2AK5ac	H4K91ac	0.743	0.058	-6.85e-01	H2BK20ac
H3K79me2	H3K9ac	0.751	0.067	-6.83e-01	H3K27ac
mRNA	H3K18ac	0.614	-6.38e-02	-6.78e-01	H3K27ac
H3K27ac	H3K4me3	0.863	0.191	-6.73e-01	H3K9ac
H3K4ac	H3K79me2	0.74	0.069	-6.71e-01	H3K27ac
H3K4me3	H3K79me2	0.712	0.047	-6.65e-01	H3K9ac
H3K27ac	H4K20me1	0.568	-9.65e-02	-6.65e-01	H4K91ac
H2BK20ac	H3K23ac	0.644	-1.38e-02	-6.58e-01	H4K5ac
H2AK5ac	H3K4ac	0.748	0.095	-6.53e-01	H2BK20ac
H3K4me2	H4K20me1	0.664	0.017	-6.47e-01	H3K4me1
H3K23ac	H3K27ac	0.689	0.049	-6.41e-01	H4K5ac
H3K18ac	H3K23ac	0.673	0.035	-6.39e-01	H4K5ac
H2AK5ac	H2BK12ac	0.73	0.096	-6.34e-01	H2BK20ac
mRNA	DNaseIHS	0.633	4.64e-04	-6.32e-01	H3K27ac
H3K23ac	H3K4me3	0.678	0.051	-6.26e-01	H3K9ac
H3K23ac	H3K9ac	0.697	0.073	-6.25e-01	H4K5ac
H3K4me3	H3K9ac	0.876	0.255	-6.21e-01	H3K27ac
H3K4me1	H4K5ac	0.678	0.059	-6.19e-01	H3K18ac

mRNA	H4K5ac	0.59	-2.72e-02	-6.17e-01	H3K27ac	mRNA	H4K5ac	0.59	-2.72e-02	-6.17e-01	H3K27ac
mRNA	H2BK12ac	0.605	-3.58e-03	-6.08e-01	H3K27ac	mRNA	H2BK12ac	0.605	-3.58e-03	-6.08e-01	H3K27ac
H2BK120ac	H4K20me1	0.615	0.013	-6.02e-01	H4K91ac	H2BK120ac	H4K20me1	0.615	0.013	-6.02e-01	H4K91ac
mRNA	H3K9ac	0.63	0.029	-6.00e-01	H3K27ac	mRNA	H3K9ac	0.63	0.029	-6.00e-01	H3K27ac
H3K27me3	H3K9ac	-6.03e-01	-1.03e-02	0.592	H3K27ac	H3K27me3	H3K9ac	-6.03e-01	-1.03e-02	0.592	H3K27ac
mRNA	H3K4me2	0.603	0.012	-5.91e-01	H3K27ac	mRNA	H3K4me2	0.603	0.012	-5.91e-01	H3K27ac
H3K27me3	H3K4ac	-6.38e-01	-4.98e-02	0.588	H3K27ac	H3K27me3	H3K4ac	-6.38e-01	-4.98e-02	0.588	H3K27ac
H3K27me3	H4K91ac	-6.43e-01	-5.88e-02	0.584	H3K27ac	H3K27me3	H4K91ac	-6.43e-01	-5.88e-02	0.584	H3K27ac
H3K4ac	H4K20me1	0.623	0.041	-5.81e-01	H4K91ac	H3K4ac	H4K20me1	0.623	0.041	-5.81e-01	H4K91ac
mRNA	H3K27ac	0.66	0.088	-5.71e-01	H2BK120ac	mRNA	H3K27ac	0.66	0.088	-5.71e-01	H2BK120ac
H3K23ac	H3K79me1	0.549	-1.54e-02	-5.64e-01	H3K4me2	H3K23ac	H3K79me1	0.549	-1.54e-02	-5.64e-01	H3K4me2
H2AK5ac	H3K79me1	0.61	0.051	-5.59e-01	H3K4ac	H2AK5ac	H3K79me1	0.61	0.051	-5.59e-01	H3K4ac
H2AK5ac	H3K4me1	0.596	0.054	-5.42e-01	H2BK20ac	H2AK5ac	H3K4me1	0.596	0.054	-5.42e-01	H2BK20ac
DNaseIHS	H3K27me3	-6.00e-01	-6.28e-02	0.537	H3K27ac	DNaseIHS	H3K27me3	-6.00e-01	-6.28e-02	0.537	H3K27ac
H3K79me1	H4K20me1	0.732	0.199	-5.33e-01	H3K4me1	H3K79me1	H4K20me1	0.732	0.199	-5.33e-01	H3K4me1
H3K4me1	H3K79me1	0.78	0.254	-5.26e-01	H3K4me2	H3K4me1	H3K79me1	0.78	0.254	-5.26e-01	H3K4me2
H3K23ac	H3K4me1	0.494	-2.63e-02	-5.20e-01	H3K4me2	H3K23ac	H3K4me1	0.494	-2.63e-02	-5.20e-01	H3K4me2
mRNA	H3K79me2	0.624	0.12	-5.04e-01	H3K27ac	mRNA	H3K79me2	0.624	0.12	-5.04e-01	H3K27ac
H2AK5ac	H3K27me3	-4.48e-01	0.046	0.494	H4K91ac	H2AK5ac	H3K27me3	-4.48e-01	0.046	0.494	H4K91ac
H3K23ac	H3K4me2	0.696	0.212	-4.84e-01	H3K4me3	H3K23ac	H3K4me2	0.696	0.212	-4.84e-01	H3K4me3
H3K27me3	H3K79me1	-5.17e-01	-3.77e-02	0.479	H3K27ac	H3K27me3	H3K79me1	-5.17e-01	-3.77e-02	0.479	H3K27ac
mRNA	H4K20me1	0.566	0.108	-4.58e-01	H3K4me1	mRNA	H4K20me1	0.566	0.108	-4.58e-01	H3K4me1
DNaseIHS	H4K20me1	0.686	0.232	-4.54e-01	H3K4me1	DNaseIHS	H4K20me1	0.686	0.232	-4.54e-01	H3K4me1
H3K79me2	H4K20me1	0.616	0.168	-4.48e-01	H3K79me1	H3K79me2	H4K20me1	0.616	0.168	-4.48e-01	H3K79me1
H3K4me2	H3K4me3	0.878	0.435	-4.43e-01	H3K27ac	H3K4me2	H3K4me3	0.878	0.435	-4.43e-01	H3K27ac
H3K23ac	H4K20me1	0.325	-1.10e-01	-4.35e-01	H3K4me2	H3K23ac	H4K20me1	0.325	-1.10e-01	-4.35e-01	H3K4me2
H2AK5ac	H3K23ac	0.51	0.077	-4.33e-01	H4K5ac	H2AK5ac	H3K23ac	0.51	0.077	-4.33e-01	H4K5ac
mRNA	H2AK5ac	0.464	0.038	-4.26e-01	H3K4ac	mRNA	H2AK5ac	0.464	0.038	-4.26e-01	H3K4ac
mRNA	H3K23ac	0.422	0.004	-4.18e-01	H3K27ac	mRNA	H3K23ac	0.422	0.004	-4.18e-01	H3K27ac
H2BK12ac	H3K9me3	-3.20e-01	0.092	0.412	H4K91ac	H2BK12ac	H3K9me3	-3.20e-01	0.092	0.412	H4K91ac
H3K27ac	H3K27me3	-6.68e-01	-2.60e-01	0.409	H4K91ac	H3K27ac	H3K27me3	-6.68e-01	-2.60e-01	0.409	H4K91ac
H2BK12ac	H3K14ac	0.432	0.026	-4.06e-01	H4K5ac	H2BK12ac	H3K14ac	0.432	0.026	-4.06e-01	H4K5ac
H3K79me1	H3K79me2	0.781	0.383	-3.98e-01	H3K4ac	H3K79me1	H3K79me2	0.781	0.383	-3.98e-01	H3K4ac
H3K4me1	H3K4me2	0.809	0.418	-3.91e-01	H3K18ac	H3K4me1	H3K4me2	0.809	0.418	-3.91e-01	H3K18ac
H3K14ac	H3K9ac	0.445	0.069	-3.75e-01	H4K5ac	H3K14ac	H3K9ac	0.445	0.069	-3.75e-01	H4K5ac
mRNA	H3K27me3	-6.13e-01	-2.53e-01	0.36	H3K27ac	mRNA	H3K27me3	-6.13e-01	-2.53e-01	0.36	H3K27ac
H3K4ac	H3K9me3	-3.77e-01	-2.56e-02	0.351	H4K91ac	H3K4ac	H3K9me3	-3.77e-01	-2.56e-02	0.351	H4K91ac
H3K27ac	H3K9me3	-3.34e-01	0.014	0.349	H4K91ac	H3K27ac	H3K9me3	-3.34e-01	0.014	0.349	H4K91ac
H3K9me3	H4K91ac	-3.98e-01	-6.18e-02	0.336	H3K27me3	H3K9me3	H4K91ac	-3.98e-01	-6.18e-02	0.336	H3K27me3
DNaseIHS	H3K9me3	-3.27e-01	0.002	0.329	H4K91ac	DNaseIHS	H3K9me3	-3.27e-01	0.002	0.329	H4K91ac
H3K14ac	H3K79me1	0.368	0.043	-3.25e-01	H4K5ac	H3K14ac	H3K79me1	0.368	0.043	-3.25e-01	H4K5ac
H2AK5ac	H3K14ac	0.409	0.085	-3.23e-01	H4K5ac	H2AK5ac	H3K14ac	0.409	0.085	-3.23e-01	H4K5ac
H3K79me1	H3K9me3	-3.28e-01	-1.29e-02	0.315	H4K91ac	H3K79me1	H3K9me3	-3.28e-01	-1.29e-02	0.315	H4K91ac
H3K14ac	H3K4me1	0.296	-7.83e-03	-3.03e-01	H3K4me2	H3K14ac	H3K4me1	0.296	-7.83e-03	-3.03e-01	H3K4me2
H3K9ac	H3K9me3	-2.99e-01	-7.70e-03	0.291	H4K91ac	H3K9ac	H3K9me3	-2.99e-01	-7.70e-03	0.291	H4K91ac
H3K9me3	H4K20me1	-3.54e-01	-7.87e-02	0.275	H3K4me1	H3K9me3	H4K20me1	-3.54e-01	-7.87e-02	0.275	H3K4me1
H3K14ac	H3K27me3	-9.19e-02	0.159	0.251	H4K5ac	H3K14ac	H3K27me3	-9.19e-02	0.159	0.251	H4K5ac
mRNA	H3K14ac	0.191	-4.41e-02	-2.35e-01	H4K5ac	mRNA	H3K14ac	0.191	-4.41e-02	-2.35e-01	H4K5ac
H3K14ac	H4K20me1	0.138	-5.01e-02	-1.88e-01	H3K79me1	H3K14ac	H4K20me1	0.138	-5.01e-02	-1.88e-01	H3K79me1
H3K14ac	H3K36me3	0.2	0.082	-1.18e-01	H2AK5ac	H3K14ac	H3K36me3	0.2	0.082	-1.18e-01	H2AK5ac
H3K36me3	H3K79me2	0.059	-2.98e-02	-8.91e-02	H3K79me1	H3K36me3	H3K79me2	0.059	-2.98e-02	-8.91e-02	H3K79me1
H3K36me3	H4K5ac	0.035	0.106	0.071	H3K27me3	H3K36me3	H4K5ac	0.035	0.106	0.071	H3K27me3
mRNA	H3K36me3	-1.19e-02	0.043	0.055	H3K27me3	mRNA	H3K36me3	-1.19e-02	0.043	0.055	H3K27me3
H2BK120ac	H3K36me3	-2.38e-02	-7.36e-02	-4.98e-02	H3K79me1	H2BK120ac	H3K36me3	-2.38e-02	-7.36e-02	-4.98e-02	H3K79me1
H3K27ac	H3K36me3	-2.16e-02	0.016	0.038	H3K27me3	H3K27ac	H3K36me3	-2.16e-02	0.016	0.038	H3K27me3
H3K36me3	H4K91ac	0.003	0.026	0.023	H3K27me3	H3K36me3	H4K91ac	0.003	0.026	0.023	H3K27me3
H3K36me3	H3K4ac	0.01	0.021	0.011	H3K27me3	H3K36me3	H3K4ac	0.01	0.021	0.011	H3K27me3
H3K36me3	H3K9ac	-4.31e-03	0.004	0.009	H3K79me1	H3K36me3	H3K9ac	-4.31e-03	0.004	0.009	H3K79me1
H2AK5ac	H3K36me3	0.159	0.167	0.008	H3K27me3	H2AK5ac	H3K36me3	0.159	0.167	0.008	H3K27me3
H3K27me3	H3K36me3	0.216	0.22	0.004	H3K79me1	H3K27me3	H3K36me3	0.216	0.22	0.004	H3K79me1
H3K36me3	H3K79me1	0.16	0.162	0.002	H3K27me3						

Table S11: **Effect of partial correlations in CD4+ cells.** The pairs of variables are ordered by decreasing difference between correlation and partial correlation. The first two colums give the variables, the Cor column gives the correlation coefficient, the PCor column gives the partial correlation coefficient, and PCor − Cor column gives the magnitude of the effect of the control set on the correlation coefficient. The last column indicates the variable Z that has the largest effect $\mathrm{Cor}(X, Y|Z) - \mathrm{Cor}(X, Y)$.

8.2 Effect matrix for IMR90 cells

variable 1	variable 2	Cor	PCor	PCor- Cor	MIV
H2AK5ac	H2BK120ac	0.919	-2.12e-01	-1.13e+00	H3K4ac
H3K23ac	H4K5ac	0.927	-1.65e-01	-1.09e+00	H3K14ac
H2AK5ac	H3K18ac	0.913	-1.68e-01	-1.08e+00	H3K4ac
H3K14ac	H3K4me3	0.87	-1.74e-01	-1.04e+00	H3K9ac
H2BK120ac	H4K5ac	0.919	-1.16e-01	-1.03e+00	H4K91ac
H2BK12ac	H3K4ac	0.945	-7.50e-02	-1.02e+00	H2BK120ac
H3K18ac	H3K79me1	0.847	-1.62e-01	-1.01e+00	H3K14ac
H3K27ac	H3K4me2	0.913	-9.16e-02	-1.00e+00	H3K9ac
H2BK20ac	H3K4ac	0.921	-7.28e-02	-9.93e-01	H2BK120ac
H3K14ac	H4K91ac	0.973	-1.40e-02	-9.87e-01	H3K4ac
H3K4me2	H3K79me2	0.861	-1.14e-01	-9.75e-01	H3K9ac
H3K27ac	H3K4me3	0.914	-5.47e-02	-9.69e-01	H3K9ac
H3K18ac	H3K79me2	0.85	-1.10e-01	-9.60e-01	H3K14ac
H3K4ac	H3K79me2	0.898	-5.66e-02	-9.55e-01	H3K14ac
H3K4ac	H3K9ac	0.942	-9.03e-03	-9.51e-01	H3K27ac
H3K23ac	H3K79me2	0.897	-5.06e-02	-9.48e-01	H3K14ac
H3K4ac	H3K79me1	0.894	-4.96e-02	-9.44e-01	H3K14ac
H3K4me3	H4K91ac	0.838	-1.04e-01	-9.42e-01	H3K9ac
DNaseIHS	H3K9ac	0.869	-7.20e-02	-9.41e-01	H3K27ac
H3K27ac	H3K79me2	0.9	-3.72e-02	-9.37e-01	H3K9ac
H3K27ac	H4K5ac	0.953	0.023	-9.30e-01	H3K4ac
H3K18ac	H3K9ac	0.929	0.002	-9.27e-01	H3K27ac
DNaseIHS	H3K23ac	0.84	-7.99e-02	-9.20e-01	H3K9ac
H2BK12ac	H3K9ac	0.854	-6.51e-02	-9.19e-01	H3K4ac
H4K20me1	H4K5ac	0.659	-2.54e-01	-9.13e-01	H3K9ac
H3K27ac	H3K79me1	0.874	-3.63e-02	-9.10e-01	H3K14ac
H2BK12ac	H4K91ac	0.951	0.042	-9.09e-01	H2BK120ac
H3K27ac	H4K91ac	0.953	0.048	-9.05e-01	H3K4ac
H2BK12ac	H3K27ac	0.893	-6.82e-03	-9.00e-01	H3K18ac
H3K9ac	H4K5ac	0.938	0.044	-8.94e-01	H3K27ac
H2BK20ac	H3K9ac	0.818	-5.66e-02	-8.75e-01	H3K4ac
H3K4ac	H3K4me2	0.894	0.029	-8.65e-01	H3K23ac
DNaseIHS	H2BK120ac	0.793	-7.14e-02	-8.64e-01	H3K4ac
DNaseIHS	H3K4ac	0.836	-2.24e-02	-8.59e-01	H3K27ac
H3K14ac	H4K5ac	0.958	0.099	-8.58e-01	H3K4ac
H3K14ac	H3K9ac	0.945	0.092	-8.52e-01	H3K27ac
H2AK5ac	H3K4me3	0.783	-5.93e-02	-8.42e-01	H3K9ac
H2BK20ac	H3K23ac	0.898	0.058	-8.40e-01	H2BK120ac
H3K23ac	H3K9ac	0.948	0.118	-8.30e-01	H3K14ac
H2BK12ac	H3K79me2	0.815	-1.21e-02	-8.28e-01	H3K4ac
H2AK5ac	H4K91ac	0.952	0.126	-8.26e-01	H3K4ac
H2BK120ac	H3K23ac	0.931	0.107	-8.24e-01	H3K14ac
H2BK20ac	H3K4me2	0.807	-1.55e-02	-8.23e-01	H3K23ac
H3K9ac	H4K20me1	0.756	-6.12e-02	-8.18e-01	H3K79me2
DNaseIHS	H4K91ac	0.822	0.011	-8.11e-01	H3K27ac
H3K4me1	H3K4me3	0.531	-2.76e-01	-8.07e-01	H3K4me2
H3K4me1	H4K91ac	0.702	-9.31e-02	-7.96e-01	H2AK5ac
H2BK120ac	H4K91ac	0.964	0.175	-7.89e-01	H3K4ac
H2BK12ac	H3K79me1	0.83	0.046	-7.84e-01	H3K4ac
H3K4ac	H4K20me1	0.712	-6.91e-02	-7.81e-01	H3K23ac
H3K23ac	H3K79me1	0.899	0.121	-7.78e-01	H3K14ac
H3K4me2	H3K9ac	0.941	0.166	-7.75e-01	H3K4me3
H3K14ac	H3K79me1	0.902	0.131	-7.71e-01	H3K23ac
DNaseIHS	H3K4me2	0.854	0.085	-7.68e-01	H3K9ac
H3K4me3	H3K79me2	0.87	0.11	-7.60e-01	H3K9ac
mRNA	H3K9ac	0.722	-3.64e-02	-7.59e-01	H3K79me2
H3K27ac	H3K4ac	0.963	0.205	-7.58e-01	H4K91ac
DNaseIHS	H4K5ac	0.832	0.075	-7.57e-01	H3K27ac
H2AK5ac	H3K79me2	0.889	0.14	-7.48e-01	H3K4ac
H3K14ac	H4K20me1	0.722	-1.82e-02	-7.40e-01	H3K23ac
H3K4ac	H3K4me1	0.712	-2.31e-02	-7.35e-01	H2AK5ac
H2BK120ac	H4K20me1	0.654	-7.73e-02	-7.31e-01	H3K23ac
mRNA	H4K5ac	0.683	-4.74e-02	-7.30e-01	H3K27ac
H2BK12ac	H3K4me1	0.741	0.02	-7.21e-01	H2AK5ac
H3K36me3	H3K4me2	0.56	-1.54e-01	-7.14e-01	H2AK5ac
mRNA	H3K14ac	0.695	-1.79e-02	-7.13e-01	H3K9ac
mRNA	H2AK5ac	0.654	-4.78e-02	-7.02e-01	H3K4ac
mRNA	H3K23ac	0.689	-9.19e-03	-6.99e-01	H3K9ac
mRNA	H3K27ac	0.716	0.021	-6.96e-01	H3K9ac
H3K4me1	H3K79me2	0.646	-4.53e-02	-6.91e-01	H2AK5ac
H3K4me3	H4K20me1	0.727	0.043	-6.85e-01	H3K9ac
H3K14ac	H3K36me3	0.596	-8.53e-02	-6.81e-01	H2AK5ac

variable 1	variable 2	Cor	PCor	PCor- Cor	MIV
H2AK5ac	H2BK120ac	0.919	-2.12e-01	-1.13e+00	H3K4ac
H3K23ac	H4K5ac	0.927	-1.65e-01	-1.09e+00	H3K14ac
H2AK5ac	H3K18ac	0.913	-1.68e-01	-1.08e+00	H3K4ac
H3K14ac	H3K4me3	0.87	-1.74e-01	-1.04e+00	H3K9ac
H2BK120ac	H4K5ac	0.919	-1.16e-01	-1.03e+00	H4K91ac
H2BK12ac	H3K4ac	0.945	-7.50e-02	-1.02e+00	H2BK120ac
H3K18ac	H3K79me1	0.847	-1.62e-01	-1.01e+00	H3K14ac
H3K27ac	H3K4me2	0.913	-9.16e-02	-1.00e+00	H3K9ac
H2BK20ac	H3K4ac	0.921	-7.28e-02	-9.93e-01	H2BK120ac
H3K14ac	H4K91ac	0.973	-1.40e-02	-9.87e-01	H3K4ac
H3K4me2	H3K79me2	0.861	-1.14e-01	-9.75e-01	H3K9ac
H3K27ac	H3K4me3	0.914	-5.47e-02	-9.69e-01	H3K9ac
H3K18ac	H3K79me2	0.85	-1.10e-01	-9.60e-01	H3K14ac
H3K4ac	H3K79me2	0.898	-5.66e-02	-9.55e-01	H3K14ac
H3K4ac	H3K9ac	0.942	-9.03e-03	-9.51e-01	H3K27ac
H3K23ac	H3K79me2	0.897	-5.06e-02	-9.48e-01	H3K14ac
H3K4ac	H3K79me1	0.894	-4.96e-02	-9.44e-01	H3K14ac
H3K4me3	H4K91ac	0.838	-1.04e-01	-9.42e-01	H3K9ac
DNaseIHS	H3K9ac	0.869	-7.20e-02	-9.41e-01	H3K27ac
H3K27ac	H3K79me2	0.9	-3.72e-02	-9.37e-01	H3K9ac
H3K27ac	H4K5ac	0.953	0.023	-9.30e-01	H3K4ac
H3K18ac	H3K9ac	0.929	0.002	-9.27e-01	H3K27ac
DNaseIHS	H3K23ac	0.84	-7.99e-02	-9.20e-01	H3K9ac
H2BK12ac	H3K9ac	0.854	-6.51e-02	-9.19e-01	H3K4ac
H4K20me1	H4K5ac	0.659	-2.54e-01	-9.13e-01	H3K9ac
H3K27ac	H3K79me1	0.874	-3.63e-02	-9.10e-01	H3K14ac
H2BK12ac	H4K91ac	0.951	0.042	-9.09e-01	H2BK120ac
H3K27ac	H4K91ac	0.953	0.048	-9.05e-01	H3K4ac
H2BK12ac	H3K27ac	0.893	-6.82e-03	-9.00e-01	H3K18ac
H3K9ac	H4K5ac	0.938	0.044	-8.94e-01	H3K27ac
H2BK20ac	H3K9ac	0.818	-5.66e-02	-8.75e-01	H3K4ac
H3K4ac	H3K4me2	0.894	0.029	-8.65e-01	H3K23ac
DNaseIHS	H2BK120ac	0.793	-7.14e-02	-8.64e-01	H3K4ac
DNaseIHS	H3K4ac	0.836	-2.24e-02	-8.59e-01	H3K27ac
H3K14ac	H4K5ac	0.958	0.099	-8.58e-01	H3K4ac
H3K14ac	H3K9ac	0.945	0.092	-8.52e-01	H3K27ac
H2AK5ac	H3K4me3	0.783	-5.93e-02	-8.42e-01	H3K9ac
H2BK20ac	H3K23ac	0.898	0.058	-8.40e-01	H2BK120ac
H3K23ac	H3K9ac	0.948	0.118	-8.30e-01	H3K14ac
H2BK12ac	H3K79me2	0.815	-1.21e-02	-8.28e-01	H3K4ac
H2AK5ac	H4K91ac	0.952	0.126	-8.26e-01	H3K4ac
H2BK120ac	H3K23ac	0.931	0.107	-8.24e-01	H3K14ac
H2BK20ac	H3K4me2	0.807	-1.55e-02	-8.23e-01	H3K23ac
H3K9ac	H4K20me1	0.756	-6.12e-02	-8.18e-01	H3K79me2
DNaseIHS	H4K91ac	0.822	0.011	-8.11e-01	H3K27ac
H3K4me1	H3K4me3	0.531	-2.76e-01	-8.07e-01	H3K4me2
H3K4me1	H4K91ac	0.702	-9.31e-02	-7.96e-01	H2AK5ac
H2BK120ac	H4K91ac	0.964	0.175	-7.89e-01	H3K4ac
H2BK12ac	H3K79me1	0.83	0.046	-7.84e-01	H3K4ac
H3K4ac	H4K20me1	0.712	-6.91e-02	-7.81e-01	H3K23ac
H3K23ac	H3K79me1	0.899	0.121	-7.78e-01	H3K14ac
H3K4me2	H3K9ac	0.941	0.166	-7.75e-01	H3K4me3
H3K14ac	H3K79me1	0.902	0.131	-7.71e-01	H3K23ac
DNaseIHS	H3K4me2	0.854	0.085	-7.68e-01	H3K9ac
H3K4me3	H3K79me2	0.87	0.11	-7.60e-01	H3K9ac
mRNA	H3K9ac	0.722	-3.64e-02	-7.59e-01	H3K79me2
H3K27ac	H3K4ac	0.963	0.205	-7.58e-01	H4K91ac
DNaseIHS	H4K5ac	0.832	0.075	-7.57e-01	H3K27ac
H2AK5ac	H3K79me2	0.889	0.14	-7.48e-01	H3K4ac
H3K14ac	H4K20me1	0.722	-1.82e-02	-7.40e-01	H3K23ac
H3K4ac	H3K4me1	0.712	-2.31e-02	-7.35e-01	H2AK5ac
H2BK120ac	H4K20me1	0.654	-7.73e-02	-7.31e-01	H3K23ac
mRNA	H4K5ac	0.683	-4.74e-02	-7.30e-01	H3K27ac
H2BK12ac	H3K4me1	0.741	0.02	-7.21e-01	H2AK5ac
H3K36me3	H3K4me2	0.56	-1.54e-01	-7.14e-01	H2AK5ac
mRNA	H3K14ac	0.695	-1.79e-02	-7.13e-01	H3K9ac
mRNA	H2AK5ac	0.654	-4.78e-02	-7.02e-01	H3K4ac
mRNA	H3K23ac	0.689	-9.19e-03	-6.99e-01	H3K9ac
mRNA	H3K27ac	0.716	0.021	-6.96e-01	H3K9ac
H3K4me1	H3K79me2	0.646	-4.53e-02	-6.91e-01	H2AK5ac
H3K4me3	H4K20me1	0.727	0.043	-6.85e-01	H3K9ac
H3K14ac	H3K36me3	0.596	-8.53e-02	-6.81e-01	H2AK5ac

H2BK12ac	H4K20me1	0.665	-9.13e-03	-6.74e-01	H3K23ac	H2BK12ac	H4K20me1	0.665	-9.13e-03	-6.74e-01	H3K23ac
mRNA	H3K4me2	0.679	0.01	-6.69e-01	H3K9ac	mRNA	H3K4me2	0.679	0.01	-6.69e-01	H3K9ac
H3K23ac	H3K4me1	0.687	0.018	-6.69e-01	H2AK5ac	H3K23ac	H3K4me1	0.687	0.018	-6.69e-01	H2AK5ac
H2BK12ac	H3K18ac	0.954	0.29	-6.63e-01	H2BK120ac	H2BK12ac	H3K18ac	0.954	0.29	-6.63e-01	H2BK120ac
H3K4me1	H3K9ac	0.628	-1.91e-02	-6.48e-01	H2AK5ac	H3K4me1	H3K9ac	0.628	-1.91e-02	-6.48e-01	H2AK5ac
H3K18ac	H3K4me1	0.68	0.036	-6.44e-01	H2AK5ac	H3K18ac	H3K4me1	0.68	0.036	-6.44e-01	H2AK5ac
H3K27ac	H3K4me1	0.651	0.01	-6.40e-01	H2AK5ac	H3K27ac	H3K4me1	0.651	0.01	-6.40e-01	H2AK5ac
DNaseIHS	H3K36me3	0.517	-1.22e-01	-6.39e-01	H2AK5ac	DNaseIHS	H3K36me3	0.517	-1.22e-01	-6.39e-01	H2AK5ac
mRNA	H2BK12ac	0.62	-1.20e-02	-6.32e-01	H3K4ac	mRNA	H2BK12ac	0.62	-1.20e-02	-6.32e-01	H3K4ac
mRNA	H2BK120ac	0.638	0.01	-6.28e-01	H3K4ac	mRNA	H2BK120ac	0.638	0.01	-6.28e-01	H3K4ac
H3K36me3	H3K4ac	0.62	0.002	-6.18e-01	H2AK5ac	H3K36me3	H3K4ac	0.62	0.002	-6.18e-01	H2AK5ac
H3K36me3	H3K79me2	0.672	0.064	-6.08e-01	H2AK5ac	H3K36me3	H3K79me2	0.672	0.064	-6.08e-01	H2AK5ac
H3K36me3	H3K4me3	0.525	-8.12e-02	-6.06e-01	H3K9ac	H3K36me3	H3K4me3	0.525	-8.12e-02	-6.06e-01	H3K9ac
H2BK120ac	H3K4me1	0.715	0.114	-6.01e-01	H2BK12ac	H2BK120ac	H3K4me1	0.715	0.114	-6.01e-01	H2BK12ac
H2BK12ac	H3K36me3	0.556	-4.41e-02	-6.00e-01	H2AK5ac	H2BK12ac	H3K36me3	0.556	-4.41e-02	-6.00e-01	H2AK5ac
H3K18ac	H3K27ac	0.958	0.36	-5.98e-01	H3K4ac	H3K18ac	H3K27ac	0.958	0.36	-5.98e-01	H3K4ac
H2AK5ac	H2BK12ac	0.93	0.338	-5.91e-01	H3K4ac	H2AK5ac	H2BK12ac	0.93	0.338	-5.91e-01	H3K4ac
H3K36me3	H4K91ac	0.603	0.016	-5.87e-01	H2AK5ac	H3K36me3	H4K91ac	0.603	0.016	-5.87e-01	H2AK5ac
H3K27me3	H3K9ac	-4.15e-01	0.172	0.587	H3K27ac	H3K27me3	H3K9ac	-4.15e-01	0.172	0.587	H3K27ac
H2BK120ac	H2BK20ac	0.974	0.402	-5.72e-01	H2BK12ac	H2BK120ac	H2BK20ac	0.974	0.402	-5.72e-01	H2BK12ac
H2BK120ac	H3K36me3	0.545	-2.26e-02	-5.68e-01	H2AK5ac	H2BK120ac	H3K36me3	0.545	-2.26e-02	-5.68e-01	H2AK5ac
H3K23ac	H3K36me3	0.606	0.041	-5.65e-01	H2AK5ac	H3K23ac	H3K36me3	0.606	0.041	-5.65e-01	H2AK5ac
H2AK5ac	H3K36me3	0.696	0.139	-5.57e-01	H3K79me2	H2AK5ac	H3K36me3	0.696	0.139	-5.57e-01	H3K79me2
H2BK12ac	H2BK20ac	0.979	0.43	-5.48e-01	H2BK120ac	H2BK12ac	H2BK20ac	0.979	0.43	-5.48e-01	H2BK120ac
H2BK20ac	H3K36me3	0.525	-1.74e-02	-5.42e-01	H2AK5ac	H2BK20ac	H3K36me3	0.525	-1.74e-02	-5.42e-01	H2AK5ac
H3K36me3	H3K9ac	0.604	0.08	-5.23e-01	H3K79me2	H3K36me3	H3K9ac	0.604	0.08	-5.23e-01	H3K79me2
H2AK5ac	H3K4ac	0.962	0.441	-5.21e-01	H4K91ac	H2AK5ac	H3K4ac	0.962	0.441	-5.21e-01	H4K91ac
H3K4me1	H3K79me1	0.703	0.19	-5.14e-01	H2AK5ac	H3K4me1	H3K79me1	0.703	0.19	-5.14e-01	H2AK5ac
H3K36me3	H3K79me1	0.66	0.154	-5.06e-01	H2AK5ac	H3K36me3	H3K79me1	0.66	0.154	-5.06e-01	H2AK5ac
H3K36me3	H3K4me1	0.669	0.188	-4.81e-01	H2AK5ac	H3K36me3	H3K4me1	0.669	0.188	-4.81e-01	H2AK5ac
H3K4me3	H3K9ac	0.955	0.492	-4.63e-01	H3K4me2	H3K4me3	H3K9ac	0.955	0.492	-4.63e-01	H3K4me2
H2AK5ac	H3K4me1	0.793	0.353	-4.40e-01	H2BK12ac	H2AK5ac	H3K4me1	0.793	0.353	-4.40e-01	H2BK12ac
H3K27me3	H4K5ac	-4.34e-01	-5.26e-03	0.429	H3K27ac	H3K27me3	H4K5ac	-4.34e-01	-5.26e-03	0.429	H3K27ac
H3K36me3	H4K20me1	0.709	0.283	-4.26e-01	H3K79me2	H3K36me3	H4K20me1	0.709	0.283	-4.26e-01	H3K79me2
H3K14ac	H3K23ac	0.977	0.553	-4.24e-01	H3K4ac	H3K14ac	H3K23ac	0.977	0.553	-4.24e-01	H3K4ac
H3K14ac	H3K27me3	-4.09e-01	-2.02e-02	0.389	H3K27ac	H3K14ac	H3K27me3	-4.09e-01	-2.02e-02	0.389	H3K27ac
H2BK120ac	H3K27me3	-3.41e-01	0.042	0.383	H4K91ac	H2BK120ac	H3K27me3	-3.41e-01	0.042	0.383	H4K91ac
H2AK5ac	H3K27me3	-3.52e-01	0.022	0.374	H3K4ac	H2AK5ac	H3K27me3	-3.52e-01	0.022	0.374	H3K4ac
H3K27me3	H4K20me1	-1.81e-01	0.157	0.338	H3K79me2	H3K27me3	H4K20me1	-1.81e-01	0.157	0.338	H3K79me2
DNaseIHS	H3K27me3	-3.93e-01	-7.73e-02	0.315	H3K27ac	DNaseIHS	H3K27me3	-3.93e-01	-7.73e-02	0.315	H3K27ac
H3K4me1	H3K4me2	0.678	0.376	-3.03e-01	H2AK5ac	H3K4me1	H3K4me2	0.678	0.376	-3.03e-01	H2AK5ac
H3K27me3	H3K4me2	-3.39e-01	-4.03e-02	0.299	H3K27ac	H3K27me3	H3K4me2	-3.39e-01	-4.03e-02	0.299	H3K27ac
H3K4me3	H3K9me3	-1.46e-01	0.116	0.262	H3K9ac	H3K4me3	H3K9me3	-1.46e-01	0.116	0.262	H3K9ac
H3K9me3	H4K91ac	-1.79e-01	0.066	0.245	H3K27me3	H3K9me3	H4K91ac	-1.79e-01	0.066	0.245	H3K27me3
H3K18ac	H3K9me3	-1.67e-01	0.059	0.227	H3K27ac	H3K18ac	H3K9me3	-1.67e-01	0.059	0.227	H3K27ac
H3K4me2	H3K9me3	-1.30e-01	0.074	0.204	H3K79me1	H3K4me2	H3K9me3	-1.30e-01	0.074	0.204	H3K79me1
DNaseIHS	H3K9me3	-1.55e-01	0.048	0.203	H3K27me3	DNaseIHS	H3K9me3	-1.55e-01	0.048	0.203	H3K27me3
H3K23ac	H3K9me3	-1.50e-01	0.038	0.187	H3K14ac	H3K23ac	H3K9me3	-1.50e-01	0.038	0.187	H3K14ac
H3K14ac	H3K9me3	-1.88e-01	-2.00e-02	0.168	H3K27me3	H3K14ac	H3K9me3	-1.88e-01	-2.00e-02	0.168	H3K27me3
H3K4ac	H3K9me3	-1.86e-01	-2.10e-02	0.165	H3K27me3	H3K4ac	H3K9me3	-1.86e-01	-2.10e-02	0.165	H3K27me3
H2BK120ac	H3K9me3	-1.60e-01	-1.85e-03	0.158	H3K4ac	H2BK120ac	H3K9me3	-1.60e-01	-1.85e-03	0.158	H3K4ac
H2AK5ac	H3K9me3	-1.56e-01	-1.19e-02	0.144	H3K79me1	H2AK5ac	H3K9me3	-1.56e-01	-1.19e-02	0.144	H3K79me1
H2BK12ac	H3K9me3	-1.62e-01	-6.79e-02	0.094	H3K4ac	H2BK12ac	H3K9me3	-1.62e-01	-6.79e-02	0.094	H3K4ac
H3K9ac	H3K9me3	-1.82e-01	-1.49e-01	0.034	H3K27ac	H3K9ac	H3K9me3	-1.82e-01	-1.49e-01	0.034	H3K27ac
H3K9me3	H4K20me1	-3.65e-02	-3.32e-02	0.003	H3K79me2						

Table S12: **Effect of partial correlations in IMR90 cells.** The pairs of variables are ordered by decreasing difference between correlation and partial correlation. The first two colums give the variables, the Cor column gives the correlation coefficient, the PCor column gives the partial correlation coefficient, and PCor − Cor column gives the magnitude of the effect of the control set on the correlation coefficient. The last column indicates the variable Z that has the largest effect $\mathrm{Cor}(X, Y|Z) - \mathrm{Cor}(X, Y)$.

8.3 Effect matrix for H1 cells

variable 1	variable 2	Cor	PCor	PCor- Cor	MIV
H3K9ac	H4K5ac	0.886	-2.55e-02	-9.12e-01	H3K27ac
H2BK12ac	H3K14ac	0.827	-4.36e-02	-8.70e-01	H3K4ac
H3K27ac	H3K4me3	0.852	-8.72e-03	-8.60e-01	H3K9ac
H2BK12ac	H3K4ac	0.848	-7.13e-03	-8.55e-01	H4K91ac
H2BK120ac	H3K14ac	0.841	0.018	-8.23e-01	H3K4ac
H3K18ac	H3K4ac	0.871	0.06	-8.11e-01	H4K91ac
H2AK5ac	H3K14ac	0.81	0.013	-7.97e-01	H3K4ac
H2BK120ac	H3K4ac	0.87	0.08	-7.90e-01	H4K91ac
H2BK120ac	H3K23ac	0.784	4.44e-04	-7.84e-01	H3K14ac
H3K14ac	H3K4me1	0.731	-5.04e-02	-7.81e-01	H2BK12ac
H3K79me1	H3K9ac	0.764	-5.87e-03	-7.70e-01	H3K4me3
H2BK120ac	H2BK12ac	0.882	0.125	-7.57e-01	H4K91ac
H3K4ac	H4K91ac	0.893	0.147	-7.47e-01	H2BK120ac
H3K14ac	H4K5ac	0.641	-9.28e-02	-7.34e-01	H3K18ac
H4K5ac	H4K91ac	0.682	-4.60e-02	-7.28e-01	H3K27ac
H3K27ac	H3K79me1	0.733	0.01	-7.23e-01	H3K9ac
H3K18ac	H3K27ac	0.771	0.05	-7.21e-01	H4K5ac
H2BK20ac	H4K91ac	0.757	0.04	-7.17e-01	H2BK12ac
H2BK12ac	H3K4me1	0.786	0.074	-7.13e-01	H2BK120ac
H2AK5ac	H3K23ac	0.79	0.082	-7.08e-01	H3K4ac
H3K4me1	H3K4me3	0.409	-2.97e-01	-7.06e-01	H3K4me2
H2AK5ac	H3K4me2	0.527	-1.75e-01	-7.02e-01	H3K4ac
H3K27ac	H3K4ac	0.741	0.043	-6.99e-01	H3K18ac
H3K4ac	H4K20me1	0.52	-1.70e-01	-6.90e-01	H3K23ac
H2BK120ac	H3K79me1	0.647	-4.02e-02	-6.87e-01	H3K4ac
H3K18ac	H3K79me2	0.631	-5.33e-02	-6.84e-01	H3K4ac
H3K4ac	H3K79me2	0.736	0.057	-6.80e-01	H3K79me1
H3K18ac	H3K4me1	0.752	0.073	-6.79e-01	H2BK12ac
H3K23ac	H3K79me2	0.674	-2.94e-03	-6.77e-01	H3K4ac
H2AK5ac	H4K91ac	0.859	0.183	-6.76e-01	H3K4ac
H3K23ac	H3K79me1	0.707	0.044	-6.63e-01	H3K4ac
H3K14ac	H3K4ac	0.898	0.239	-6.59e-01	H3K23ac
H3K79me2	H4K5ac	0.652	-4.45e-03	-6.57e-01	H3K27ac
H3K4ac	H3K9ac	0.693	0.038	-6.55e-01	H3K27ac
H2BK20ac	H3K4ac	0.751	0.098	-6.53e-01	H2BK12ac
H2BK120ac	H3K18ac	0.887	0.235	-6.52e-01	H4K91ac
H2BK120ac	H3K9ac	0.583	-6.64e-02	-6.49e-01	H3K18ac
DNaseIHS	H3K79me2	0.533	-1.14e-01	-6.47e-01	H3K79me1
H3K14ac	H3K18ac	0.864	0.218	-6.46e-01	H3K4ac
H3K27ac	H4K91ac	0.706	0.062	-6.44e-01	H3K18ac
mRNA	H4K5ac	0.656	0.013	-6.42e-01	H3K27ac
H2AK5ac	H3K4ac	0.87	0.23	-6.40e-01	H4K91ac
H3K79me2	H3K9ac	0.742	0.105	-6.37e-01	H3K79me1
H3K23ac	H3K4ac	0.852	0.216	-6.36e-01	H3K14ac
H2BK120ac	H2BK20ac	0.805	0.172	-6.32e-01	H2BK12ac
H3K4me3	H4K91ac	0.57	-6.20e-02	-6.32e-01	H3K9ac
H3K4ac	H3K4me3	0.632	0.004	-6.28e-01	H3K9ac
H4K20me1	H4K91ac	0.472	-1.54e-01	-6.26e-01	H3K79me1
H3K9ac	H4K91ac	0.635	0.014	-6.21e-01	H3K27ac
H3K14ac	H3K4me3	0.618	4.17e-04	-6.17e-01	H3K9ac
H2BK20ac	H3K23ac	0.73	0.114	-6.17e-01	H2BK12ac
H3K4me2	H4K5ac	0.686	0.073	-6.12e-01	H3K4me3
H2BK12ac	H3K27ac	0.572	-3.62e-02	-6.08e-01	H3K18ac
H2AK5ac	H3K4me3	0.459	-1.45e-01	-6.04e-01	H3K4ac
H2AK5ac	H3K27ac	0.598	8.22e-04	-5.97e-01	H3K4ac
DNaseIHS	H3K23ac	0.499	-9.40e-02	-5.93e-01	H3K4me2
H3K14ac	H3K27ac	0.671	0.079	-5.92e-01	H3K18ac
H3K9ac	H4K20me1	0.456	-1.34e-01	-5.90e-01	H3K79me1
DNaseIHS	H3K14ac	0.532	-5.40e-02	-5.86e-01	H3K18ac
mRNA	H3K4me3	0.661	0.082	-5.80e-01	H3K9ac
H3K36me3	H3K79me1	0.584	0.016	-5.68e-01	H3K79me2
H3K23ac	H3K4me3	0.566	0.004	-5.63e-01	H3K4me2
H3K4me1	H3K9me3	0.482	-7.61e-02	-5.58e-01	H3K27me3
H3K4me3	H3K79me2	0.71	0.159	-5.51e-01	H3K9ac
mRNA	H3K79me2	0.599	0.05	-5.49e-01	H3K79me1
H3K14ac	H4K20me1	0.586	0.041	-5.45e-01	H3K23ac
H3K36me3	H4K91ac	0.502	-3.75e-02	-5.40e-01	H2AK5ac
H2BK12ac	H3K4me2	0.647	0.113	-5.35e-01	H3K18ac
DNaseIHS	H3K18ac	0.61	0.076	-5.34e-01	H3K4me2
H3K36me3	H3K79me2	0.564	0.035	-5.29e-01	H3K79me1
H3K9me3	H4K20me1	0.498	-2.57e-02	-5.24e-01	H3K36me3
H3K18ac	H4K5ac	0.774	0.254	-5.19e-01	H3K27ac

variable 1	variable 2	Cor	PCor	PCor- Cor	MIV
H3K9ac	H4K5ac	0.886	-2.55e-02	-9.12e-01	H3K27ac
H2BK12ac	H3K14ac	0.827	-4.36e-02	-8.70e-01	H3K4ac
H3K27ac	H3K4me3	0.852	-8.72e-03	-8.60e-01	H3K9ac
H2BK12ac	H3K4ac	0.848	-7.13e-03	-8.55e-01	H4K91ac
H2BK120ac	H3K14ac	0.841	0.018	-8.23e-01	H3K4ac
H3K18ac	H3K4ac	0.871	0.06	-8.11e-01	H4K91ac
H2AK5ac	H3K14ac	0.81	0.013	-7.97e-01	H3K4ac
H2BK120ac	H3K4ac	0.87	0.08	-7.90e-01	H4K91ac
H2BK120ac	H3K23ac	0.784	4.44e-04	-7.84e-01	H3K14ac
H3K14ac	H3K4me1	0.731	-5.04e-02	-7.81e-01	H2BK12ac
H3K79me1	H3K9ac	0.764	-5.87e-03	-7.70e-01	H3K4me3
H2BK120ac	H2BK12ac	0.882	0.125	-7.57e-01	H4K91ac
H3K4ac	H4K91ac	0.893	0.147	-7.47e-01	H2BK120ac
H3K14ac	H4K5ac	0.641	-9.28e-02	-7.34e-01	H3K18ac
H4K5ac	H4K91ac	0.682	-4.60e-02	-7.28e-01	H3K27ac
H3K27ac	H3K79me1	0.733	0.01	-7.23e-01	H3K9ac
H3K18ac	H3K27ac	0.771	0.05	-7.21e-01	H4K5ac
H2BK20ac	H4K91ac	0.757	0.04	-7.17e-01	H2BK12ac
H2BK12ac	H3K4me1	0.786	0.074	-7.13e-01	H2BK120ac
H2AK5ac	H3K23ac	0.79	0.082	-7.08e-01	H3K4ac
H3K4me1	H3K4me3	0.409	-2.97e-01	-7.06e-01	H3K4me2
H2AK5ac	H3K4me2	0.527	-1.75e-01	-7.02e-01	H3K4ac
H3K27ac	H3K4ac	0.741	0.043	-6.99e-01	H3K18ac
H3K4ac	H4K20me1	0.52	-1.70e-01	-6.90e-01	H3K23ac
H2BK120ac	H3K79me1	0.647	-4.02e-02	-6.87e-01	H3K4ac
H3K18ac	H3K79me2	0.631	-5.33e-02	-6.84e-01	H3K4ac
H3K4ac	H3K79me2	0.736	0.057	-6.80e-01	H3K79me1
H3K18ac	H3K4me1	0.752	0.073	-6.79e-01	H2BK12ac
H3K23ac	H3K79me2	0.674	-2.94e-03	-6.77e-01	H3K4ac
H2AK5ac	H4K91ac	0.859	0.183	-6.76e-01	H3K4ac
H3K23ac	H3K79me1	0.707	0.044	-6.63e-01	H3K4ac
H3K14ac	H3K4ac	0.898	0.239	-6.59e-01	H3K23ac
H3K79me2	H4K5ac	0.652	-4.45e-03	-6.57e-01	H3K27ac
H3K4ac	H3K9ac	0.693	0.038	-6.55e-01	H3K27ac
H2BK20ac	H3K4ac	0.751	0.098	-6.53e-01	H2BK12ac
H2BK120ac	H3K18ac	0.887	0.235	-6.52e-01	H4K91ac
H2BK120ac	H3K9ac	0.583	-6.64e-02	-6.49e-01	H3K18ac
DNaseIHS	H3K79me2	0.533	-1.14e-01	-6.47e-01	H3K79me1
H3K14ac	H3K18ac	0.864	0.218	-6.46e-01	H3K4ac
H3K27ac	H4K91ac	0.706	0.062	-6.44e-01	H3K18ac
mRNA	H4K5ac	0.656	0.013	-6.42e-01	H3K27ac
H2AK5ac	H3K4ac	0.87	0.23	-6.40e-01	H4K91ac
H3K79me2	H3K9ac	0.742	0.105	-6.37e-01	H3K79me1
H3K23ac	H3K4ac	0.852	0.216	-6.36e-01	H3K14ac
H2BK120ac	H2BK20ac	0.805	0.172	-6.32e-01	H2BK12ac
H3K4me3	H4K91ac	0.57	-6.20e-02	-6.32e-01	H3K9ac
H3K4ac	H3K4me3	0.632	0.004	-6.28e-01	H3K9ac
H4K20me1	H4K91ac	0.472	-1.54e-01	-6.26e-01	H3K79me1
H3K9ac	H4K91ac	0.635	0.014	-6.21e-01	H3K27ac
H3K14ac	H3K4me3	0.618	4.17e-04	-6.17e-01	H3K9ac
H2BK20ac	H3K23ac	0.73	0.114	-6.17e-01	H2BK12ac
H3K4me2	H4K5ac	0.686	0.073	-6.12e-01	H3K4me3
H2BK12ac	H3K27ac	0.572	-3.62e-02	-6.08e-01	H3K18ac
H2AK5ac	H3K4me3	0.459	-1.45e-01	-6.04e-01	H3K4ac
H2AK5ac	H3K27ac	0.598	8.22e-04	-5.97e-01	H3K4ac
DNaseIHS	H3K23ac	0.499	-9.40e-02	-5.93e-01	H3K4me2
H3K14ac	H3K27ac	0.671	0.079	-5.92e-01	H3K18ac
H3K9ac	H4K20me1	0.456	-1.34e-01	-5.90e-01	H3K79me1
DNaseIHS	H3K14ac	0.532	-5.40e-02	-5.86e-01	H3K18ac
mRNA	H3K4me3	0.661	0.082	-5.80e-01	H3K9ac
H3K36me3	H3K79me1	0.584	0.016	-5.68e-01	H3K79me2
H3K23ac	H3K4me3	0.566	0.004	-5.63e-01	H3K4me2
H3K4me1	H3K9me3	0.482	-7.61e-02	-5.58e-01	H3K27me3
H3K4me3	H3K79me2	0.71	0.159	-5.51e-01	H3K9ac
mRNA	H3K79me2	0.599	0.05	-5.49e-01	H3K79me1
H3K14ac	H4K20me1	0.586	0.041	-5.45e-01	H3K23ac
H3K36me3	H4K91ac	0.502	-3.75e-02	-5.40e-01	H2AK5ac
H2BK12ac	H3K4me2	0.647	0.113	-5.35e-01	H3K18ac
DNaseIHS	H3K18ac	0.61	0.076	-5.34e-01	H3K4me2
H3K36me3	H3K79me2	0.564	0.035	-5.29e-01	H3K79me1
H3K9me3	H4K20me1	0.498	-2.57e-02	-5.24e-01	H3K36me3
H3K18ac	H4K5ac	0.774	0.254	-5.19e-01	H3K27ac

H3K18ac	H4K20me1	0.498	-1.66e-02	-5.14e-01	H3K14ac	H3K18ac	H4K20me1	0.498	-1.66e-02	-5.14e-01	H3K14ac
H2BK12ac	H2BK20ac	0.863	0.353	-5.10e-01	H2BK120ac	H2BK12ac	H2BK20ac	0.863	0.353	-5.10e-01	H2BK120ac
DNaseIHS	H3K4me3	0.705	0.201	-5.04e-01	H3K4me2	DNaseIHS	H3K4me3	0.705	0.201	-5.04e-01	H3K4me2
H3K23ac	H4K20me1	0.638	0.14	-4.98e-01	H3K79me1	H3K23ac	H4K20me1	0.638	0.14	-4.98e-01	H3K79me1
H3K14ac	H3K23ac	0.889	0.398	-4.91e-01	H3K4ac	H3K14ac	H3K23ac	0.889	0.398	-4.91e-01	H3K4ac
H2BK120ac	H3K4me3	0.542	0.052	-4.90e-01	H3K18ac	H2BK120ac	H3K4me3	0.542	0.052	-4.90e-01	H3K18ac
H3K4me1	H3K9ac	0.424	-6.46e-02	-4.88e-01	H3K18ac	H3K4me1	H3K9ac	0.424	-6.46e-02	-4.88e-01	H3K18ac
H3K79me1	H3K79me2	0.877	0.391	-4.87e-01	H3K9ac	H3K79me1	H3K79me2	0.877	0.391	-4.87e-01	H3K9ac
DNaseIHS	H2BK120ac	0.507	0.021	-4.86e-01	H3K18ac	DNaseIHS	H2BK120ac	0.507	0.021	-4.86e-01	H3K18ac
H3K79me1	H4K20me1	0.701	0.218	-4.83e-01	H3K79me2	H3K79me1	H4K20me1	0.701	0.218	-4.83e-01	H3K79me2
H2BK12ac	H3K27me3	0.526	0.048	-4.78e-01	H3K4me1	H2BK12ac	H3K27me3	0.526	0.048	-4.78e-01	H3K4me1
H3K36me3	H3K4ac	0.537	0.062	-4.74e-01	H2AK5ac	H3K36me3	H3K4ac	0.537	0.062	-4.74e-01	H2AK5ac
H3K23ac	H3K27me3	0.503	0.033	-4.70e-01	H3K4me1	H3K23ac	H3K27me3	0.503	0.033	-4.70e-01	H3K4me1
mRNA	H3K4ac	0.502	0.036	-4.66e-01	H3K27ac	mRNA	H3K4ac	0.502	0.036	-4.66e-01	H3K27ac
H2BK20ac	H3K79me1	0.455	-6.34e-03	-4.62e-01	H3K4ac	H2BK20ac	H3K79me1	0.455	-6.34e-03	-4.62e-01	H3K4ac
H3K27ac	H3K36me3	0.372	-8.43e-02	-4.57e-01	H3K79me1	H3K27ac	H3K36me3	0.372	-8.43e-02	-4.57e-01	H3K79me1
DNaseIHS	H4K20me1	0.616	0.161	-4.55e-01	H3K79me1	DNaseIHS	H4K20me1	0.616	0.161	-4.55e-01	H3K79me1
H4K20me1	H4K5ac	0.311	-1.41e-01	-4.52e-01	H3K79me1	H4K20me1	H4K5ac	0.311	-1.41e-01	-4.52e-01	H3K79me1
H2BK20ac	H3K9ac	0.286	-1.61e-01	-4.48e-01	H3K4ac	H2BK20ac	H3K9ac	0.286	-1.61e-01	-4.48e-01	H3K4ac
H2BK20ac	H3K36me3	0.426	-2.02e-02	-4.46e-01	H2BK12ac	H2BK20ac	H3K36me3	0.426	-2.02e-02	-4.46e-01	H2BK12ac
H3K4me3	H4K20me1	0.502	0.06	-4.43e-01	H3K79me1	H3K4me3	H4K20me1	0.502	0.06	-4.43e-01	H3K79me1
H2AK5ac	H4K20me1	0.553	0.112	-4.40e-01	H3K23ac	H2AK5ac	H4K20me1	0.553	0.112	-4.40e-01	H3K23ac
H3K27me3	H3K79me1	0.249	-1.85e-01	-4.34e-01	H3K4me1	H3K27me3	H3K79me1	0.249	-1.85e-01	-4.34e-01	H3K4me1
DNaseIHS	H3K27ac	0.569	0.141	-4.28e-01	H3K4me3	DNaseIHS	H3K27ac	0.569	0.141	-4.28e-01	H3K4me3
H2BK20ac	H3K79me2	0.433	0.012	-4.21e-01	H3K4ac	H2BK20ac	H3K79me2	0.433	0.012	-4.21e-01	H3K4ac
mRNA	H2BK120ac	0.405	-1.49e-02	-4.20e-01	H3K18ac	mRNA	H2BK120ac	0.405	-1.49e-02	-4.20e-01	H3K18ac
H3K27me3	H3K4me1	0.637	0.219	-4.17e-01	H2BK20ac	H3K27me3	H3K4me1	0.637	0.219	-4.17e-01	H2BK20ac
H3K27me3	H3K36me3	0.42	0.006	-4.14e-01	H3K4me1	H3K27me3	H3K36me3	0.42	0.006	-4.14e-01	H3K4me1
H3K4me3	H3K9ac	0.942	0.534	-4.08e-01	H3K27ac	H3K4me3	H3K9ac	0.942	0.534	-4.08e-01	H3K27ac
H2BK120ac	H3K9me3	0.4	-5.19e-03	-4.05e-01	H2BK12ac	H2BK120ac	H3K9me3	0.4	-5.19e-03	-4.05e-01	H2BK12ac
mRNA	H4K20me1	0.348	-5.65e-02	-4.05e-01	H3K79me1	mRNA	H4K20me1	0.348	-5.65e-02	-4.05e-01	H3K79me1
H3K27me3	H4K20me1	0.543	0.144	-3.99e-01	H3K4me1	H3K27me3	H4K20me1	0.543	0.144	-3.99e-01	H3K4me1
H3K36me3	H4K20me1	0.644	0.251	-3.93e-01	H3K79me1	H3K36me3	H4K20me1	0.644	0.251	-3.93e-01	H3K79me1
mRNA	H2BK12ac	0.326	-6.34e-02	-3.89e-01	H3K4ac	mRNA	H2BK12ac	0.326	-6.34e-02	-3.89e-01	H3K4ac
H3K4me1	H3K79me1	0.611	0.224	-3.88e-01	H3K4ac	H3K4me1	H3K79me1	0.611	0.224	-3.88e-01	H3K4ac
H2AK5ac	H3K9me3	0.426	0.042	-3.84e-01	H2BK12ac	H2AK5ac	H3K9me3	0.426	0.042	-3.84e-01	H2BK12ac
H3K4me1	H3K4me2	0.673	0.295	-3.78e-01	H3K18ac	H3K4me1	H3K4me2	0.673	0.295	-3.78e-01	H3K18ac
H3K4ac	H3K9me3	0.362	-5.69e-03	-3.68e-01	H2BK12ac	H3K4ac	H3K9me3	0.362	-5.69e-03	-3.68e-01	H2BK12ac
mRNA	H2AK5ac	0.416	0.052	-3.65e-01	H3K79me1	mRNA	H2AK5ac	0.416	0.052	-3.65e-01	H3K79me1
H2BK20ac	H3K27me3	0.537	0.18	-3.57e-01	H3K4me1	H2BK20ac	H3K27me3	0.537	0.18	-3.57e-01	H3K4me1
H2BK20ac	H3K27ac	0.377	0.041	-3.36e-01	H3K4ac	H2BK20ac	H3K27ac	0.377	0.041	-3.36e-01	H3K4ac
H3K18ac	H3K27me3	0.447	0.126	-3.21e-01	H3K4me1	H3K18ac	H3K27me3	0.447	0.126	-3.21e-01	H3K4me1
H3K4me2	H3K9me3	0.307	0.02	-2.87e-01	H3K4me1	H3K4me2	H3K9me3	0.307	0.02	-2.87e-01	H3K4me1
H2BK20ac	H3K4me3	0.238	-3.82e-02	-2.76e-01	H3K18ac	H2BK20ac	H3K4me3	0.238	-3.82e-02	-2.76e-01	H3K18ac
H3K79me1	H3K9me3	0.319	0.05	-2.69e-01	H4K20me1	H3K79me1	H3K9me3	0.319	0.05	-2.69e-01	H4K20me1
H3K27me3	H3K79me2	0.199	-6.38e-02	-2.63e-01	H4K20me1	H3K27me3	H3K79me2	0.199	-6.38e-02	-2.63e-01	H4K20me1
H3K36me3	H3K9ac	0.425	0.175	-2.50e-01	H3K79me1	H3K36me3	H3K9ac	0.425	0.175	-2.50e-01	H3K79me1
DNaseIHS	H3K9me3	0.359	0.121	-2.38e-01	H4K20me1	DNaseIHS	H3K9me3	0.359	0.121	-2.38e-01	H4K20me1
mRNA	H3K36me3	0.356	0.128	-2.28e-01	H3K79me1	mRNA	H3K36me3	0.356	0.128	-2.28e-01	H3K79me1
H3K27ac	H3K27me3	0.018	-1.85e-01	-2.03e-01	H3K18ac	H3K27ac	H3K27me3	0.018	-1.85e-01	-2.03e-01	H3K18ac
H3K9ac	H3K9me3	0.086	-9.55e-02	-1.81e-01	H3K14ac	H3K9ac	H3K9me3	0.086	-9.55e-02	-1.81e-01	H3K14ac
H3K27me3	H4K5ac	3.52e-04	-1.46e-01	-1.46e-01	H3K18ac	H3K27me3	H4K5ac	3.52e-04	-1.46e-01	-1.46e-01	H3K18ac
H3K9me3	H4K5ac	-9.57e-04	-8.25e-02	-8.15e-02	H3K18ac	H3K9me3	H4K5ac	-9.57e-04	-8.25e-02	-8.15e-02	H3K18ac
H3K27me3	H3K4me3	0.193	0.149	-4.44e-02	H3K4me2	H3K27me3	H3K4me3	0.193	0.149	-4.44e-02	H3K4me2
H3K27ac	H3K9me3	0.036	0.016	-1.95e-02	H3K18ac						

Table S13: **Effect of partial correlations in H1 cells.** The pairs of variables are ordered by decreasing difference between correlation and partial correlation. The first two colums give the variables, the Cor column gives the correlation coefficient, the PCor column gives the partial correlation coefficient, and PCor − Cor column gives the magnitude of the effect of the control set on the correlation coefficient. The last column indicates the variable Z that has the largest effect $\mathrm{Cor}(X, Y|Z) - \mathrm{Cor}(X, Y)$.

8.4 Consenus effect matrix

Figure S28 shows the consensus effect matrix. The color code is the following: blue means a decrease in all cell types, light blue a decrease in two cell types, red an increase in all cell types, pink an increase in two cell types. The histone modification responsible for the most part of the effect is written in black when it is shared by all cell types, and in grey when it is shared by two cell types (provided these two cell types also share the sign of the effect).

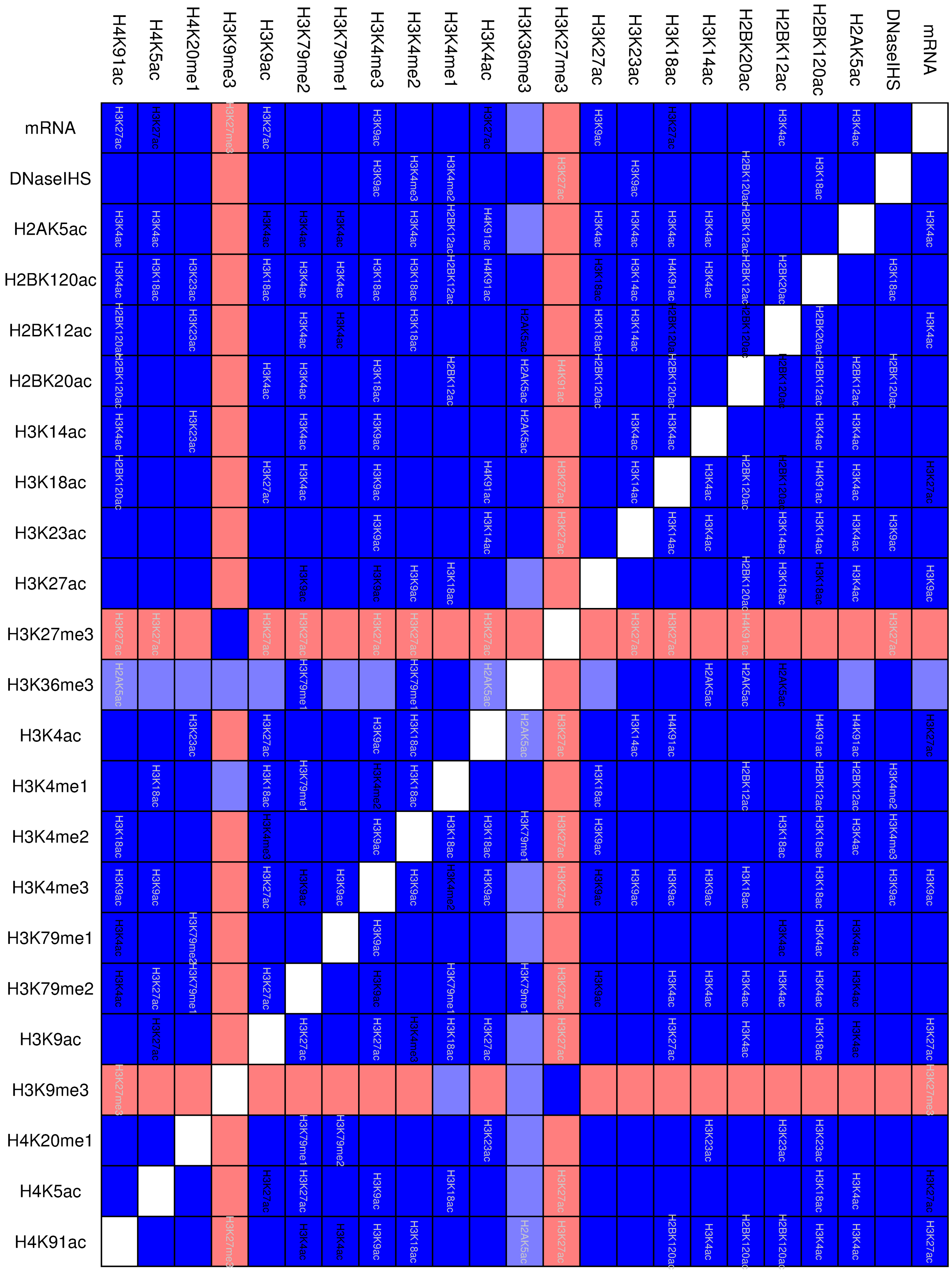

Figure S28: **Consensus effect matrix.** Blue means a decrease in all cell types, light blue a decrease in two cell types, red an increase in all cell types, pink an increase in two cell types. The histone modification responsible for the most part of the effect is written in black when it is shared by all cell types, and in grey when it is shared by two cell types (provided these two cell types also share the sign of the effect.

9 Scatter plots of the residuals

The partial correlation between X and Y with respect to a control group Z is the same as the correlation between X_Z and Y_Z, where X_Z is the residual of X after it is regressed against Z, and Y_Z is the residual of Y after it is regressed against Z. Below we show scatter plots of Y_Z against X_Z for all genes and for some gene groups in CD4+ data.

The gene groups are not present in Main Document. Two of them are based on the sequence and are cell-type-independent: the promoters of genes are known to have a bimodal distribution for their CpG content. We separated the genes according to the class of their promoter: LCP genes for low-CpG-promoter genes and HCP genes for high-CpG-promoter genes. Figure S29 shows the CpG content distribution of the promoters computed on the region [-1000,+500] around the TSS using the formula in [14].

The three other groups are based on a rough clustering of the data based on H3K27me3 and H3K4me3 only. Genes with low H3K4me3 and high H3K27me3 are repressed, genes with high H3K4me3 and low H3K27me3 are active and genes with high levels of both variables are bivalent. Figure S30 shows the groups in CD4+ cells.

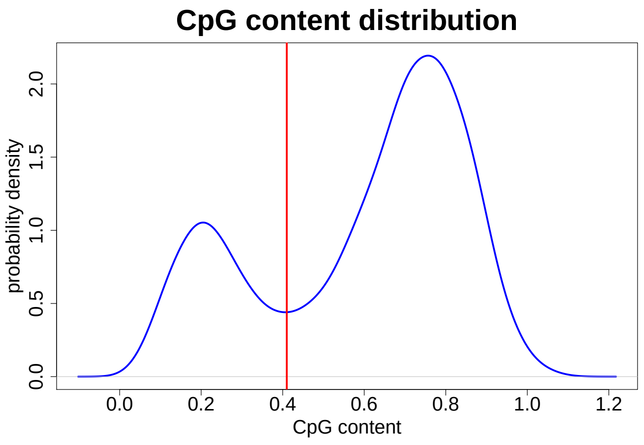

Figure S29: **Distribution of the CpG content in promoters.** The distribution is bimodal with a threshold of 0.41 (red vertical line).

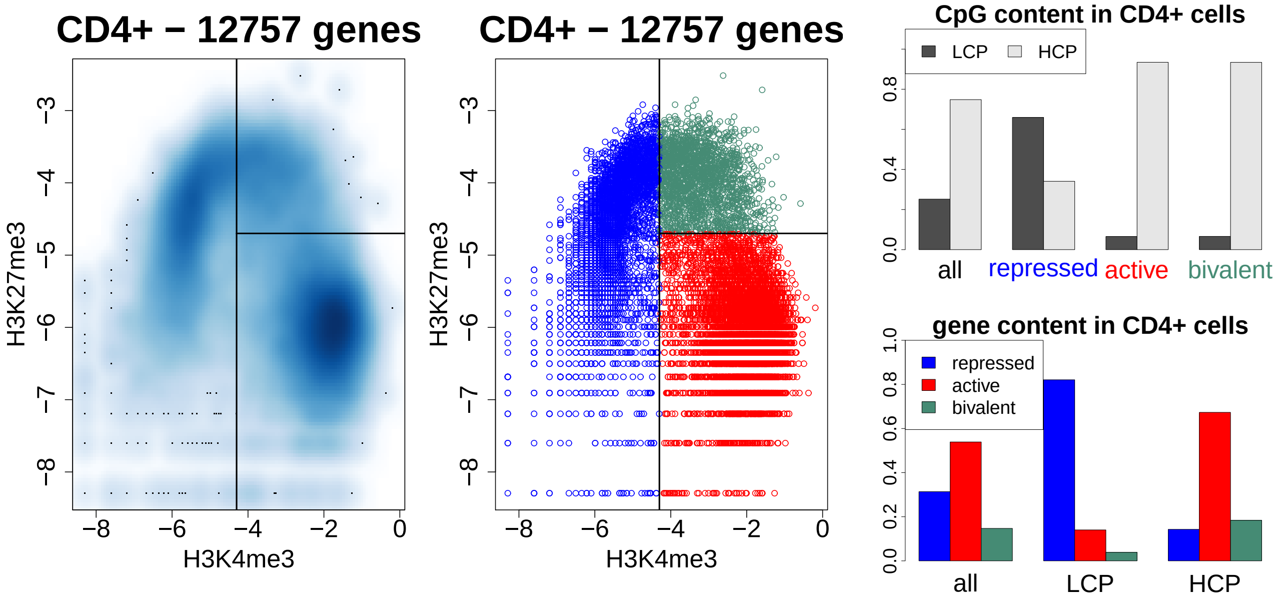

Figure S30: **The gene clusters and their CpG distributions in CD4+ cells.** LCP stands for low-CpG-promoter, HCP for high-CpG-promoter. **First column:** smooth scatter plot of the data and cluster boundaries when they are linear. **Second column:** clustered data. **Third column, top:** distribution of the CpG content in the various clusters. **Third column, bottom:** distribution of the gene activity in the various CpG types.

9.1 H3K36me3 against mRNA

We first plot in Fig. S31 the scatter plot of mRNA and H3K36me3 without any regression, simply to see the standard correlation, both using numerical data and rank-transformed data. As we can see, ranking the data has not destroyed the structure of the scatter plot.

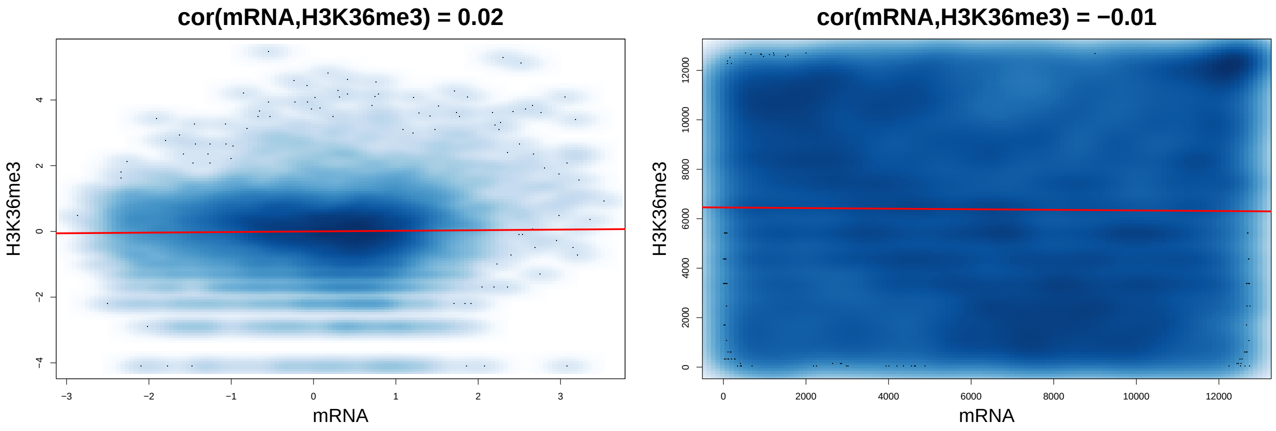

Figure S31: **Scatter plots of H3K36me3 against mRNA. Left:** numerical data. **Right:** rank data.

The data was rank-transformed. Then mRNA was regressed against all variables except itself and H3K36me3. Similarly, H3K36me3 was regressed against all variables except itself and mRNA. The scatter plot of the residuals of H3K36me3 against the residuals of mRNA is shown at the bottom of Fig. S32.

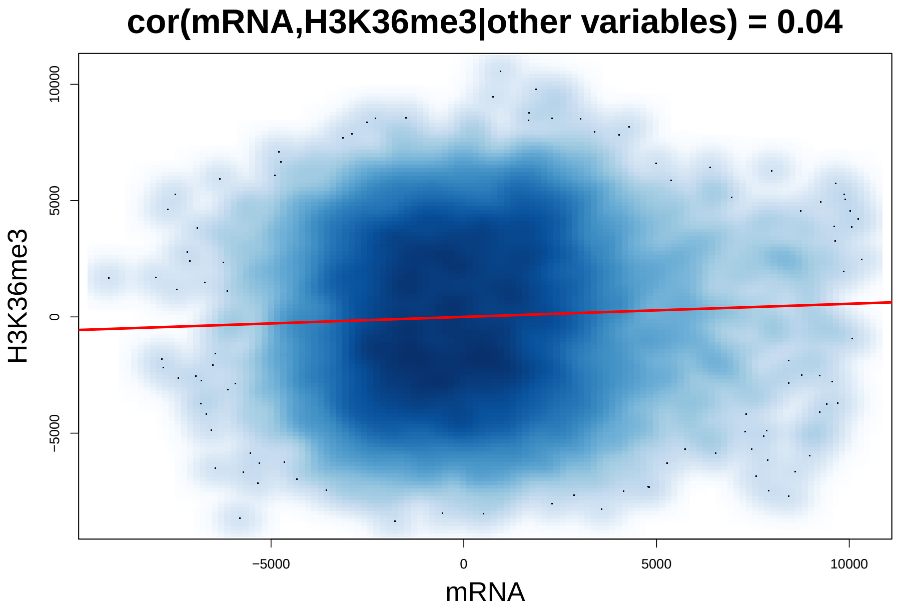

Figure S32: **Scatter plots of the residuals of H3K36me3 against the residuals of mRNA after regression against all other variables.**

Before rank-transforming the data, the genes were split into various groups: LCP and HCP, or repressed active and bivalent. The same procedure as above was then aplpied to each group. The resulting scatter plots are shown in Fig. S33.

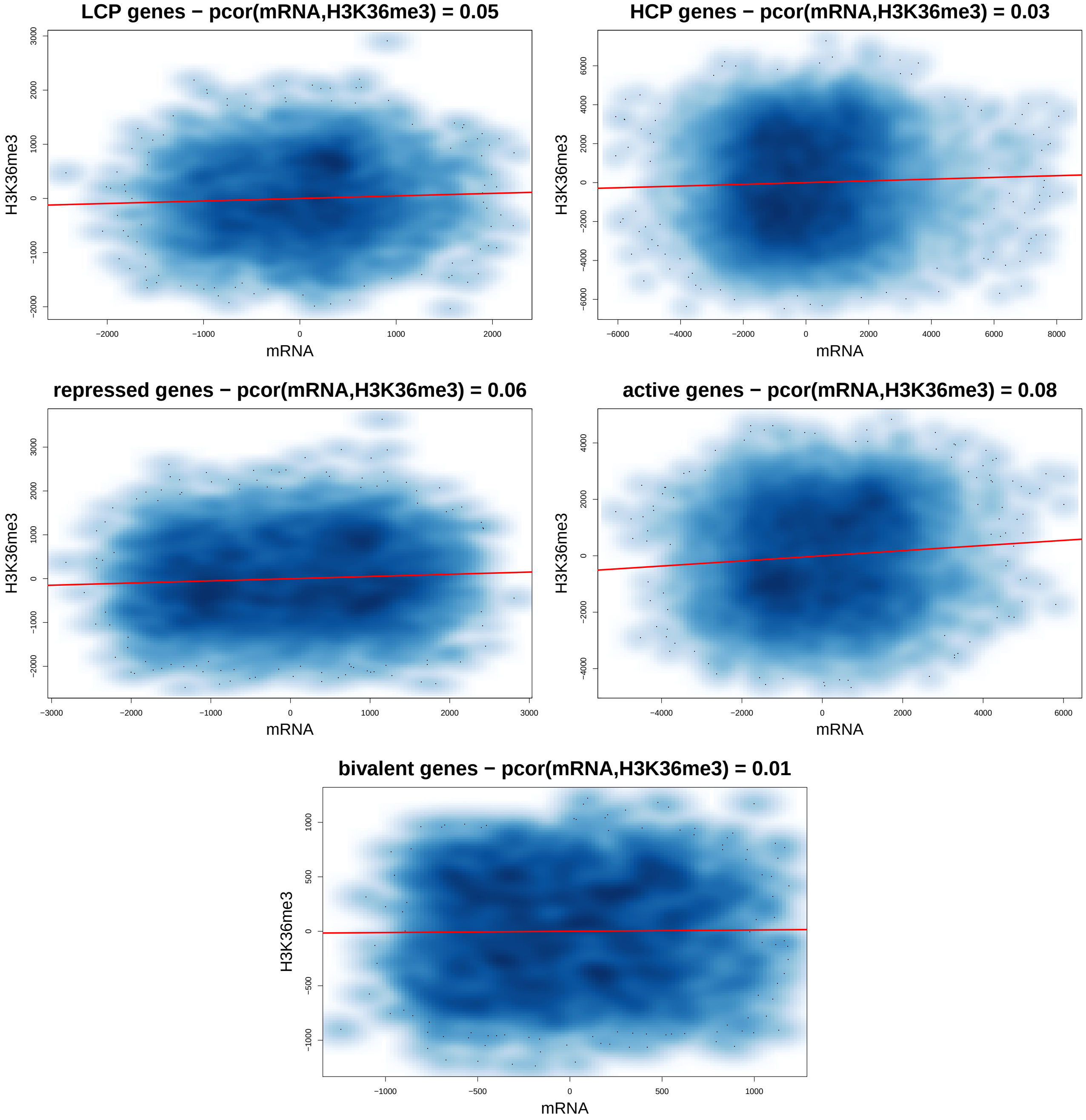

Figure S33: **Residuals of mRNA against residuals of H3K36me3 for various gene groups. 1st row left:** LCP genes. **1st row right:** HCP genes. **2nd row left:** repressed genes. **2nd row right:** active genes. **3rd row:** bivalent genes.

9.2 H3K9me3 against H3K27me3

We first plot in Fig. S34 the scatter plot of H3K27me3 and H3K9me3 without any regression, simply to see the standard correlation, both using numerical data and rank-transformed data. As we can see, ranking the data has not destroyed the structure of the scatter plot.

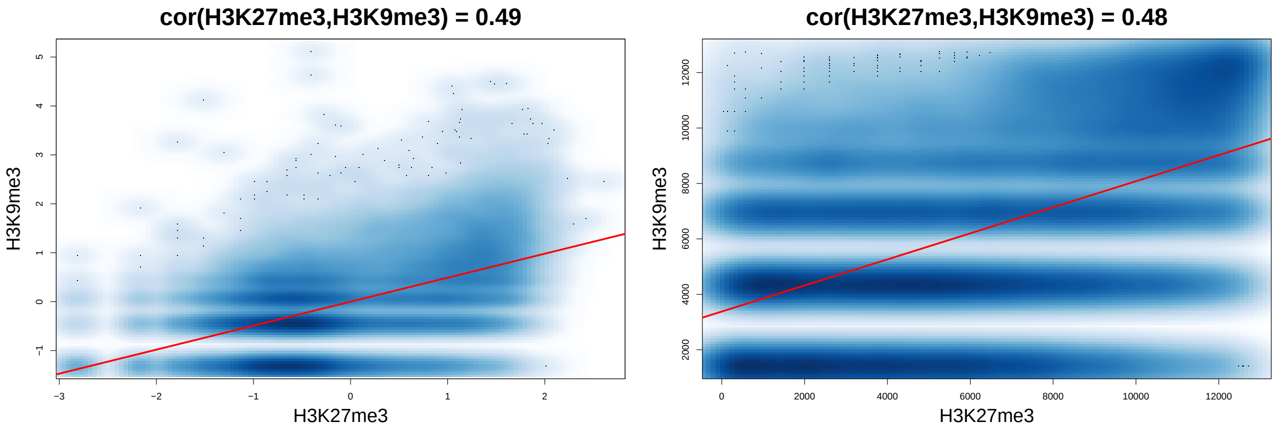

Figure S34: **Scatter plots of H3K9me3 against H3K27me3. Left:** numerical data. **Right:** rank data.

The data was rank-transformed. Then H3K27me3 was regressed against all variables except itself and H3K9me3. Similarly, H3K9me3 was regressed against all variables except itself and H3K27me3. The scatter plot of the residuals of H3K9me3 against the residuals of H3K27me3 is shown in Fig. S35.

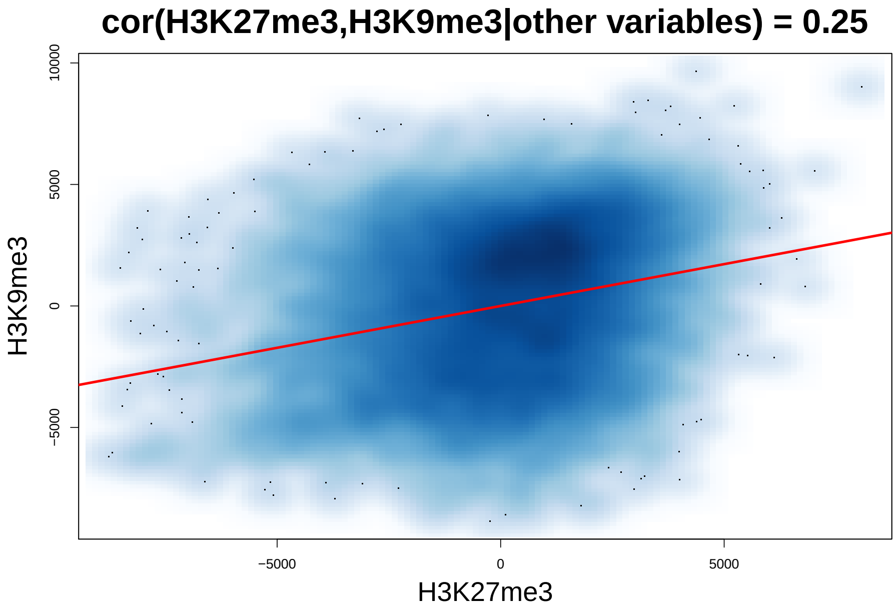

Figure S35: **Scatter plots of the residuals of H3K9me3 against the residuals of H3K27me3 after regression against all other variables.**

Before rank-transforming the data, the genes were split into various groups: LCP and HCP, or repressed active and bivalent. The same procedure as above was then aplpied to each group. The resulting scatter plots are shown in Fig. S36.

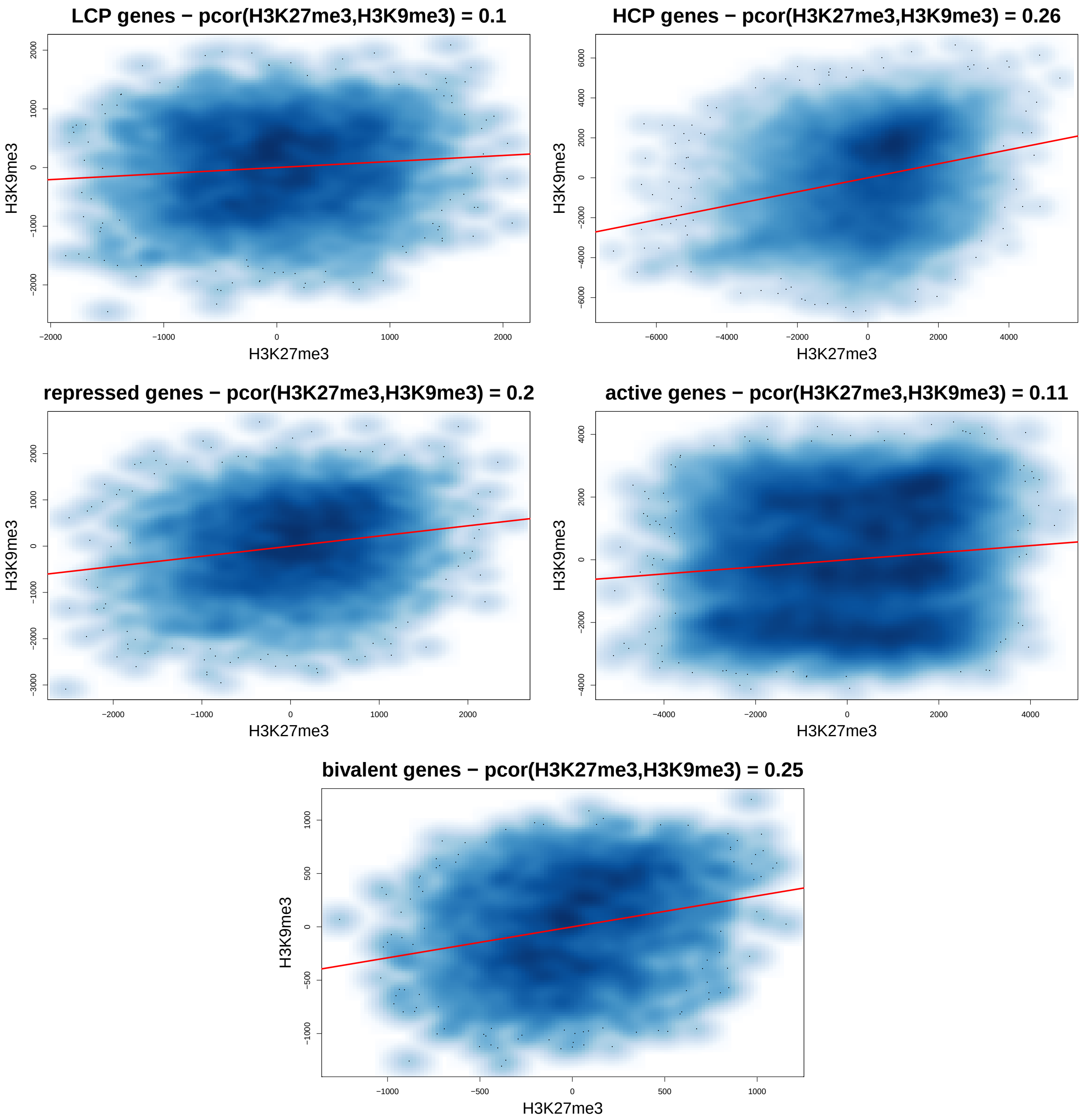

Figure S36: **Residuals of H3K9me3 against residuals of H3K27me3 for various gene groups. 1st row left:** LCP genes. **1st row right:** HCP genes. **2nd row left:** repressed genes. **2nd row right:** active genes. **3rd row:** bivalent genes.

9.3 H3K79me2 against H3K4me1

We first plot in Fig. S37 the scatter plot of H3K4me1 and H3K79me2 without any regression, simply to see the standard correlation, both using numerical data and rank-transformed data. As we can see, ranking the data has not destroyed the structure of the scatter plot.

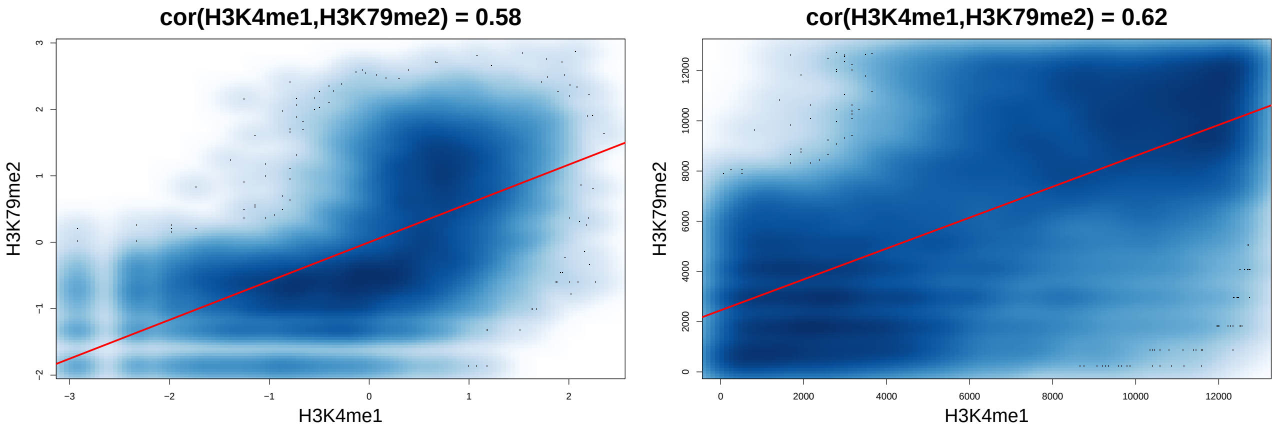

Figure S37: **Scatter plots of H3K79me2 against H3K4me1. Left:** numerical data. **Right:** rank data.

The data was rank-transformed. Then H3K4me1 was regressed against all variables except itself and H3K79me2. Similarly, H3K79me2 was regressed against all variables except itself and H3K4me1. The scatter plot of the residuals of H3K79me2 against the residuals of H3K4me1 is shown in Fig. S38.

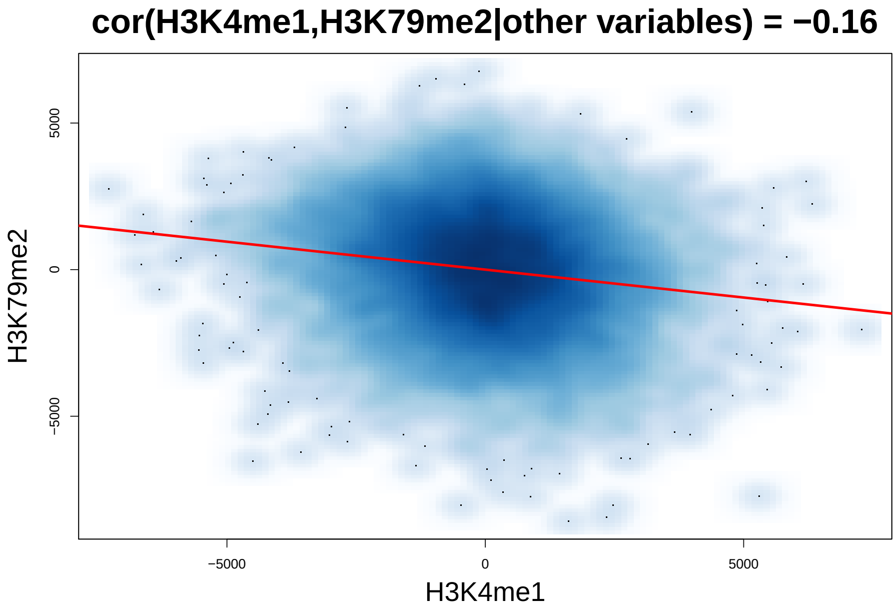

Figure S38: **Scatter plots of the residuals of H3K79me2 against the residuals of H3K4me1 after regression against all other variables.**

Before rank-transforming the data, the genes were split into various groups: LCP and HCP, or repressed active and bivalent. The same procedure as above was then aplpied to each group. The resulting scatter plots are shown in Fig. S39.

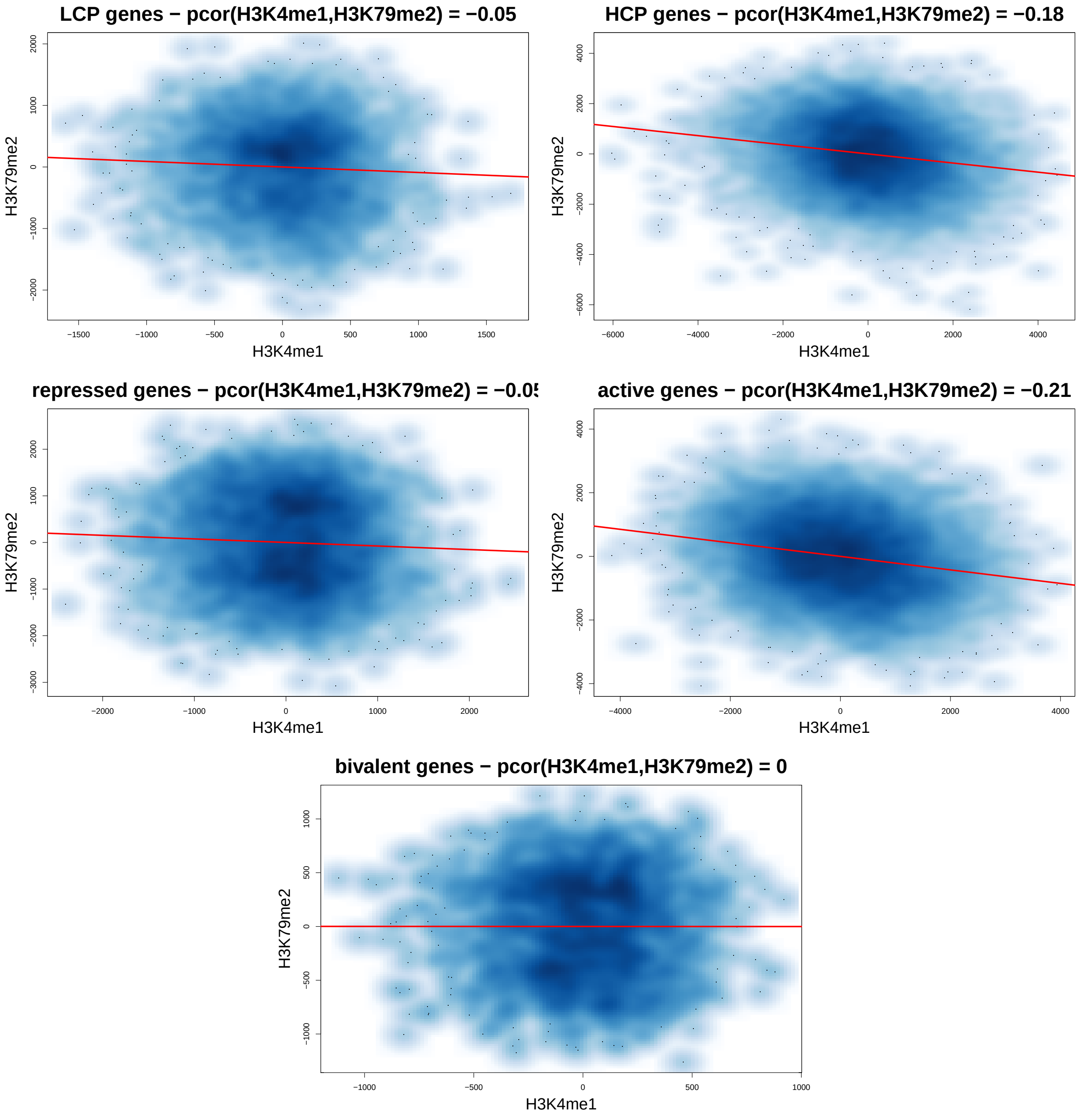

Figure S39: **Residuals of H3K79me2 against residuals of H3K4me1 for various gene groups. 1st row left:** LCP genes. **1st row right:** HCP genes. **2nd row left:** repressed genes. **2nd row right:** active genes. **3rd row:** bivalent genes.

9.4 H3K4me3 against H3K27me3

We first plot in Fig. S40 the scatter plot of H3K27me3 and H3K4me3 without any regression, simply to see the standard correlation, both using numerical data and rank-transformed data. As we can see, ranking the data has not destroyed the structure of the scatter plot.

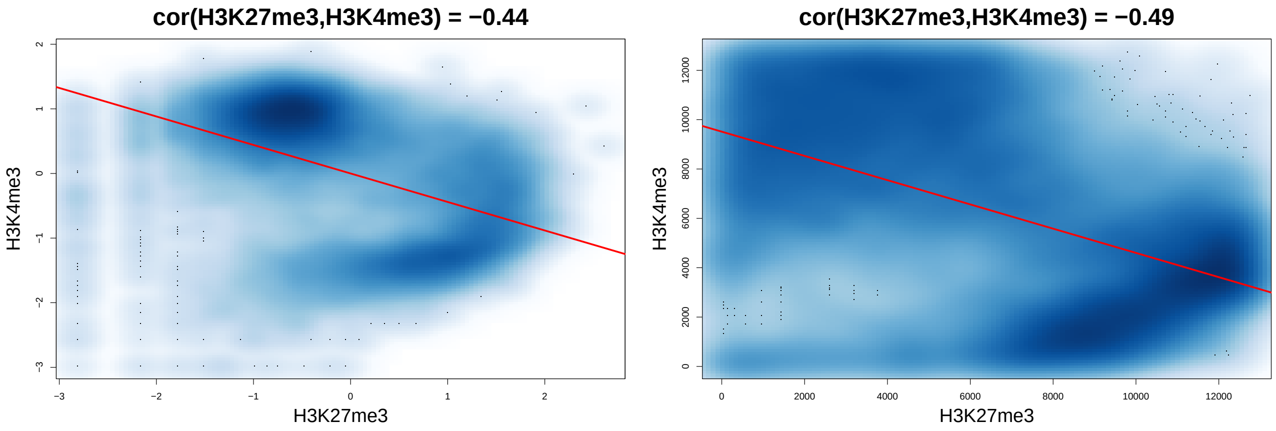

Figure S40: **Scatter plots of H3K4me3 against H3K27me3. Left:** numerical data. **Right:** rank data.

The data was rank-transformed. Then H3K27me3 was regressed against all variables except itself and H3K4me3. Similarly, H3K4me3 was regressed against all variables except itself and H3K27me3. The scatter plot of the residuals of H3K4me3 against the residuals of H3K27me3 is shown in Fig. S41.

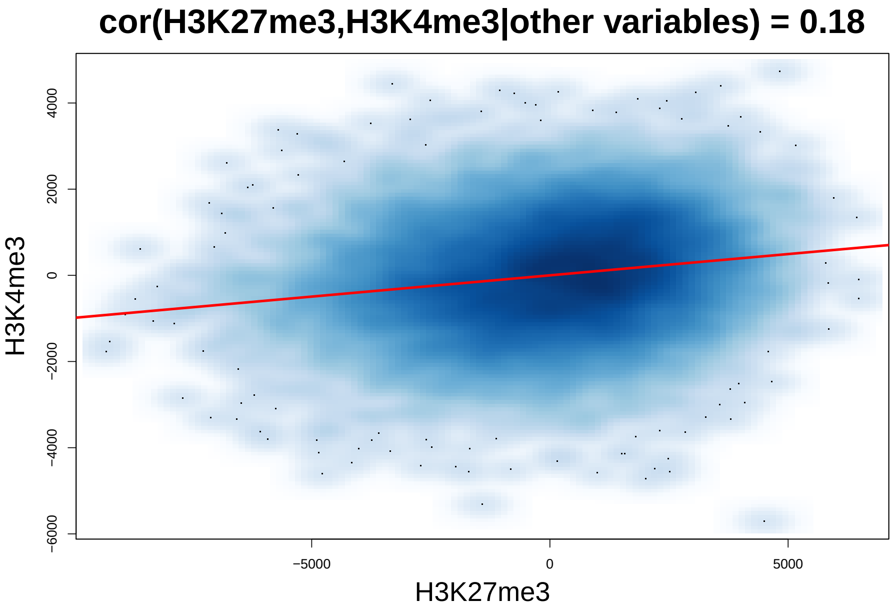

Figure S41: **Scatter plots of the residuals of H3K4me3 against the residuals of H3K27me3 after regression against all other variables.**

Before rank-transforming the data, the genes were split into various groups: LCP and HCP, or repressed active and bivalent. The same procedure as above was then aplpied to each group. The resulting scatter plots are shown in Fig. S42.

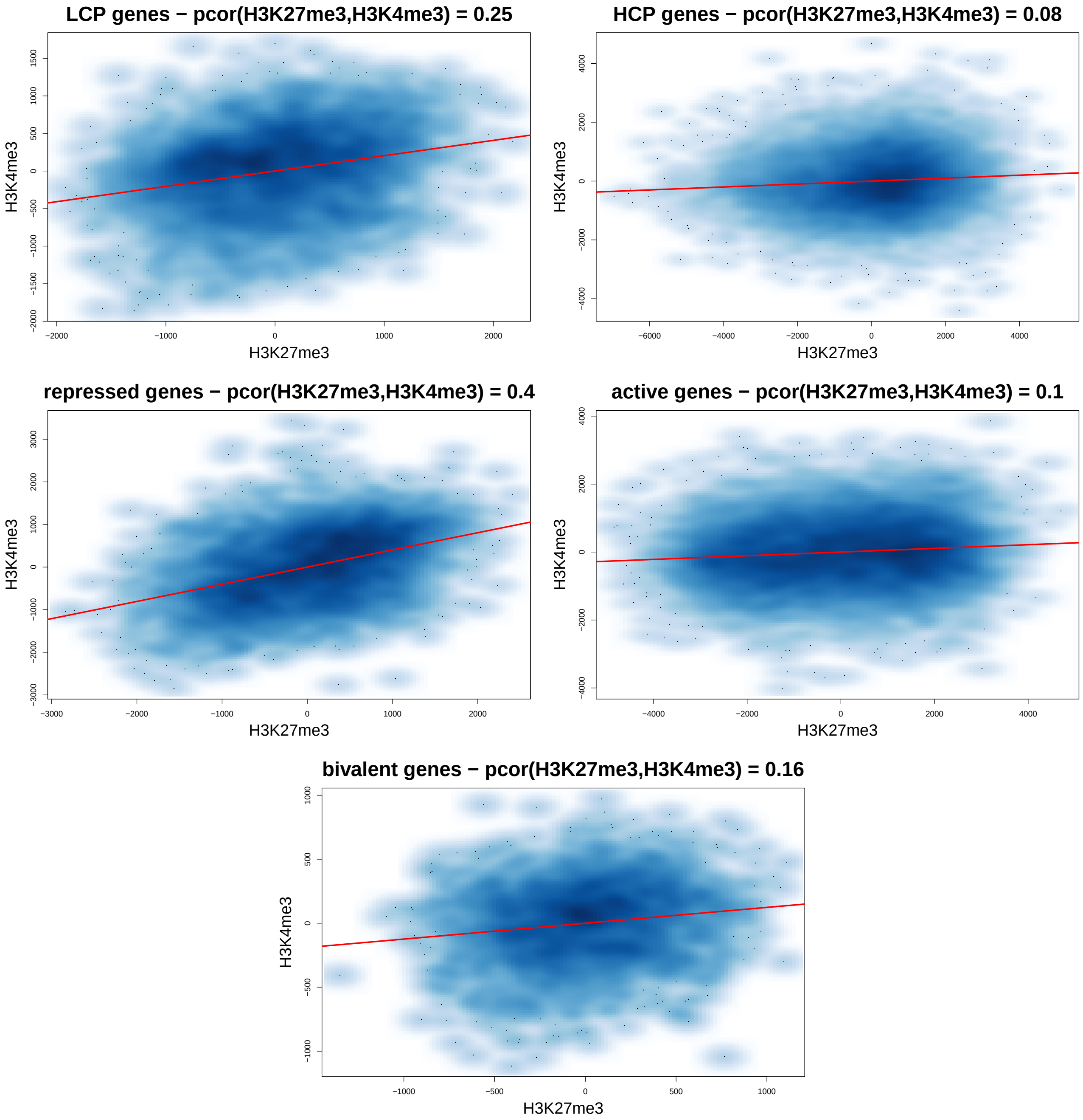

Figure S42: **Residuals of H3K4me3 against residuals of H3K27me3 for various gene groups. 1st row left:** LCP genes. **1st row right:** HCP genes. **2nd row left:** repressed genes. **2nd row right:** active genes. **3rd row:** bivalent genes.

9.5 H4K5ac against H4K20me1

We first plot in Fig. S43 the scatter plot of H4K20me1 and H4K5ac without any regression, simply to see the standard correlation, both using numerical data and rank-transformed data. As we can see, ranking the data has not destroyed the structure of the scatter plot.

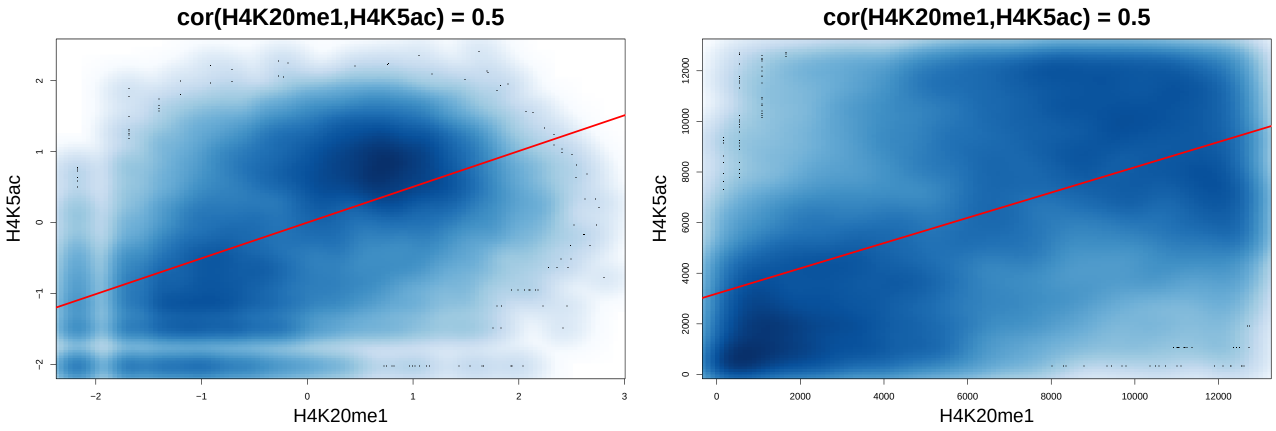

Figure S43: **Scatter plots of H4K5ac against H4K20me1. Left:** numerical data. **Right:** rank data.

The data was rank-transformed. Then H4K20me1 was regressed against all variables except itself and H4K5ac. Similarly, H4K5ac was regressed against all variables except itself and H4K20me1. The scatter plot of the residuals of H4K5ac against the residuals of H4K20me1 is shown in Fig. S44.

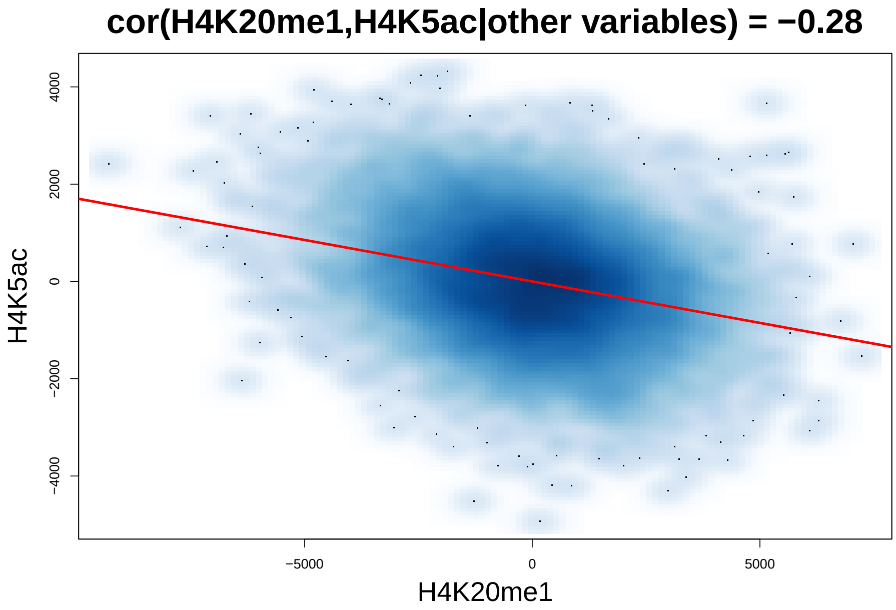

Figure S44: **Scatter plots of the residuals of H4K5ac against the residuals of H4K20me1 after regression against all other variables.**

Before rank-transforming the data, the genes were split into various groups: LCP and HCP, or repressed active and bivalent. The same procedure as above was then aplpied to each group. The resulting scatter plots are shown in Fig. S45.

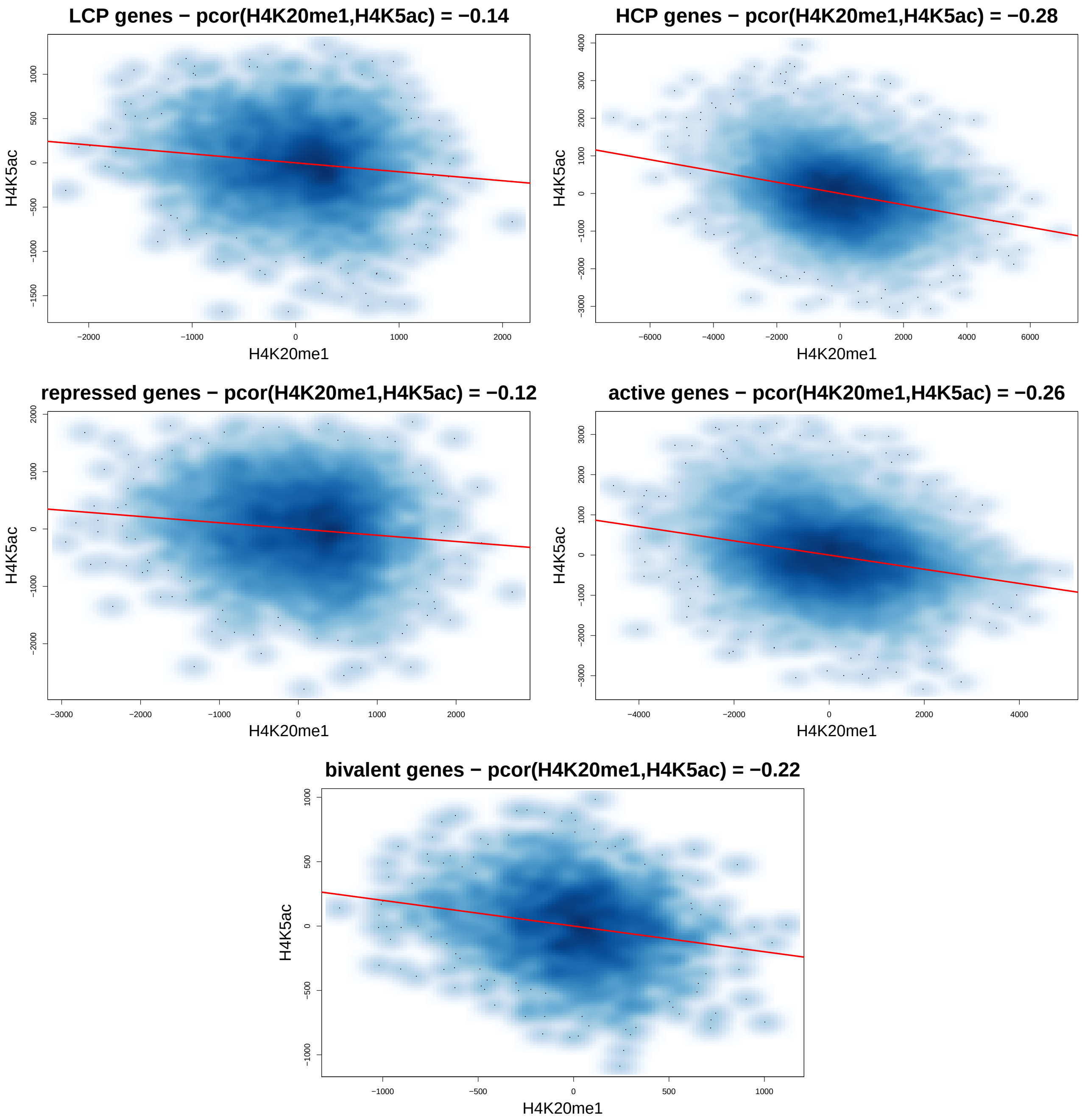

Figure S45: **Residuals of H4K5ac against residuals of H4K20me1 for various gene groups. 1st row left:** LCP genes. **1st row right:** HCP genes. **2nd row left:** repressed genes. **2nd row right:** active genes. **3rd row:** bivalent genes.

10 Explaining away pairs of histone modifications

We first condition $\mathrm{Cor}(X, Y)$ on a single variable Z. We repeat the operation for every possible Z in the dataset and identify the Z_1 that leads to the biggest discrepancy between $\mathrm{Cor}(X, Y)$ and $\mathrm{Cor}(X, Y|Z_1)$, i.e. the control variable that has the highest impact on the correlation. This left subplots show on the x-axis the variables ordered by impact, and on the y-axis $\mathrm{Cor}(X, Y|Z)$. The base line is given by $\mathrm{Cor}(X, Y)$, so that the length of bars shows the magnitude of the impact.

Once Z_1 is identified, we continue with a greedy procedure. All variables (X, Y and other control variables W) are regressed against Z_1 and their residuals (X_{Z_1}, Y_{Z_1} and W_{Z_1}) are kept for further analysis. We then look for the Z_2 that leads to the biggest discrepancy between $\mathrm{Cor}(X, Y|Z_1) = \mathrm{Cor}(X_{Z_1}, Y_{Z-1})$ and $\mathrm{Cor}(X_{Z_1}, Y_{Z_1}|Z_{2Z_1})$. And so on, until all possible control variables have been exhausted. This procedure gives the right subplots where $\mathrm{Cor}(X, Y|Z)$ is monitored as Z increases in size, and allows to check which variables have the most effect.

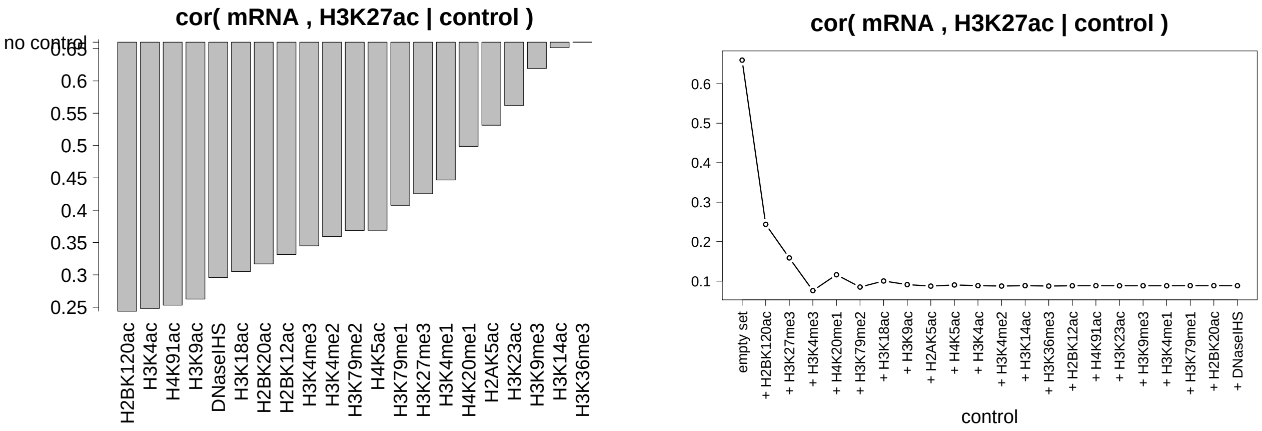

Figure S46: **Explaining away the pair mRNA-H3K27ac. Left:** $\mathrm{Cor}(\mathrm{mRNA}, \mathrm{H3K27ac}|Z)$, when Z is a single variable. **Right:** $\mathrm{Cor}(\mathrm{mRNA}, \mathrm{H3K27ac}|Z)$, when Z grows, as explained in Main Document "Section From correlations to partial correlations: explaining away".

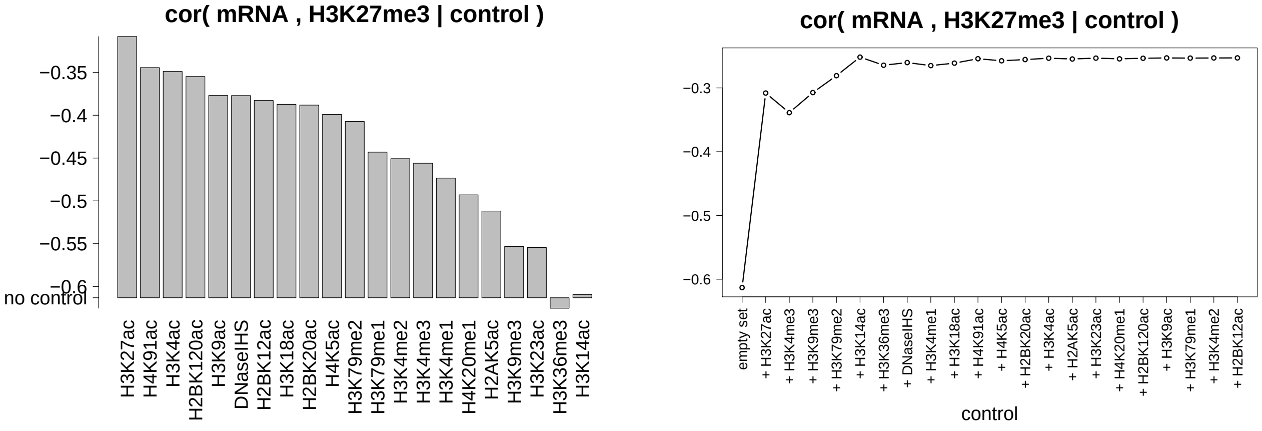

Figure S47: **Explaining away the pair mRNA-H3K27me3. Left:** $\mathrm{Cor}(\mathrm{mRNA}, \mathrm{H3K27me3}|Z)$, when Z is a single variable. **Right:** $\mathrm{Cor}(\mathrm{mRNA}, \mathrm{H3K27me3}|Z)$, when Z grows, as explained in Main Document "Section From correlations to partial correlations: explaining away".

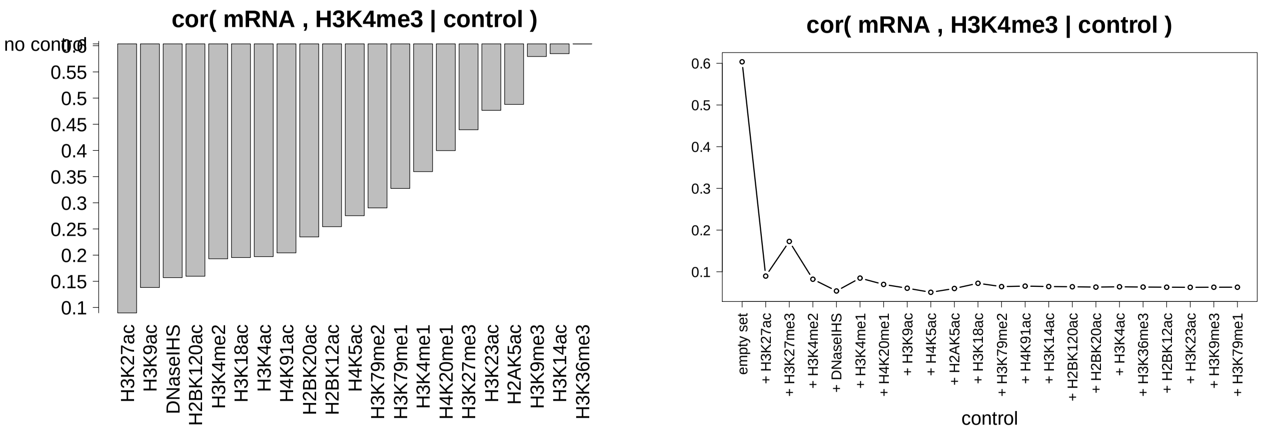

Figure S48: **Explaining away the pair mRNA-H3K4me3. Left:** $\mathrm{Cor}(\mathrm{mRNA}, \mathrm{H3K4me3}|Z)$, when Z is a single variable. **Right:** $\mathrm{Cor}(\mathrm{mRNA}, \mathrm{H3K4me3}|Z)$, when Z grows, as explained in Main Document "Section From correlations to partial correlations: explaining away".

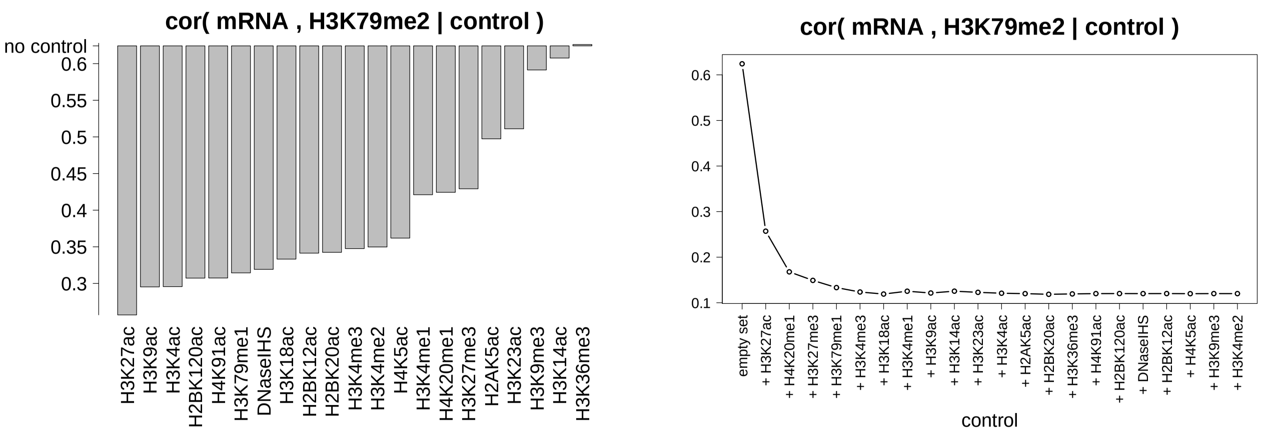

Figure S49: **Explaining away the pair mRNA-H3K79me2. Left:** $\mathrm{Cor}(\mathrm{mRNA}, \mathrm{H3K79me2}|Z)$, when Z is a single variable. **Right:** $\mathrm{Cor}(\mathrm{mRNA}, \mathrm{H3K79me2}|Z)$, when Z grows, as explained in Main Document "Section From correlations to partial correlations: explaining away".

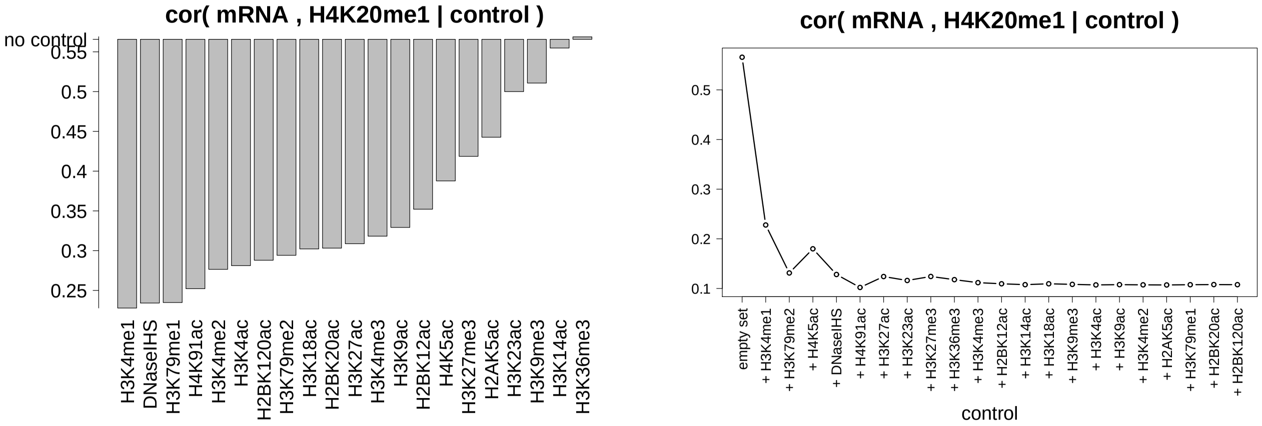

Figure S50: **Explaining away the pair mRNA-H4K20me1. Left:** $\mathrm{Cor}(\mathrm{mRNA}, \mathrm{H4K20me1}|Z)$, when Z is a single variable. **Right:** $\mathrm{Cor}(\mathrm{mRNA}, \mathrm{H4K20me1}|Z)$, when Z grows, as explained in Main Document "Section From correlations to partial correlations: explaining away".

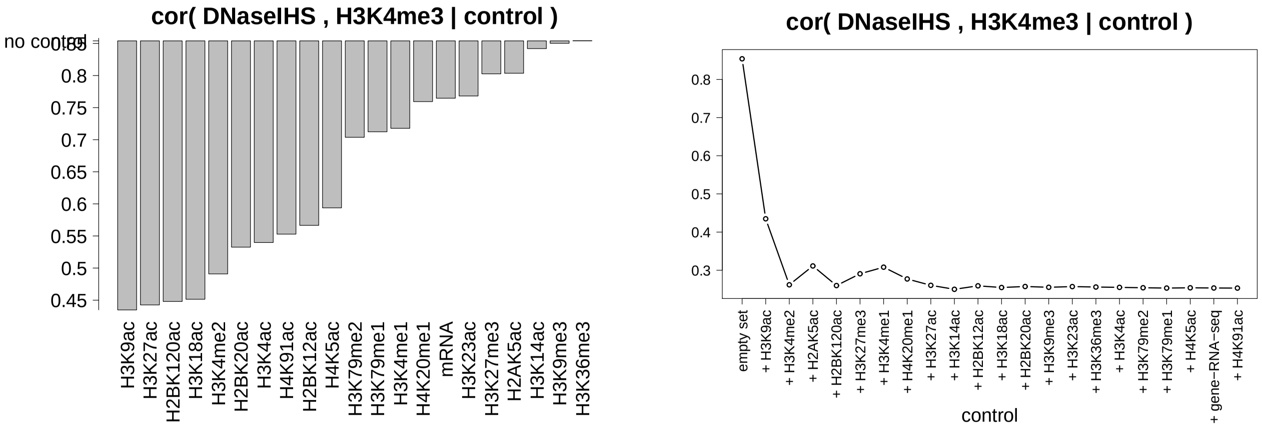

Figure S51: **Explaining away the pair DNaseIHS-H3K4me3. Left:** $\mathrm{Cor}(\mathrm{DNaseIHS}, \mathrm{H3K4me3}|Z)$, when Z is a single variable. **Right:** $\mathrm{Cor}(\mathrm{DNaseIHS}, \mathrm{H3K4me3}|Z)$, when Z grows, as explained in Main Document "Section From correlations to partial correlations: explaining away".

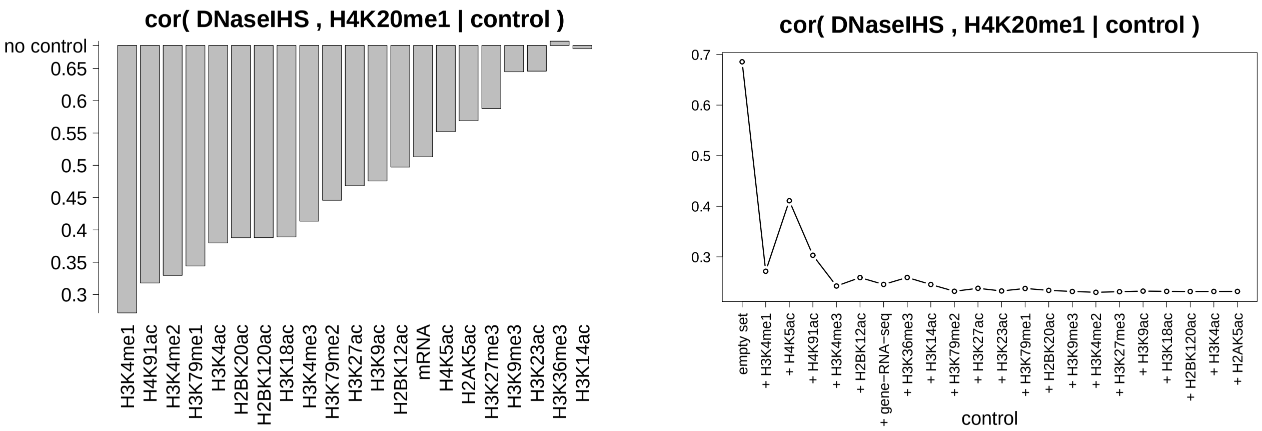

Figure S52: **Explaining away the pair DNaseIHS-H4K20me1. Left:** Cor(DNaseIHS, H4K20me1$|Z$), when Z is a single variable. **Right:** Cor(DNaseIHS, H4K20me1$|Z$), when Z grows, as explained in Main Document "Section From correlations to partial correlations: explaining away".

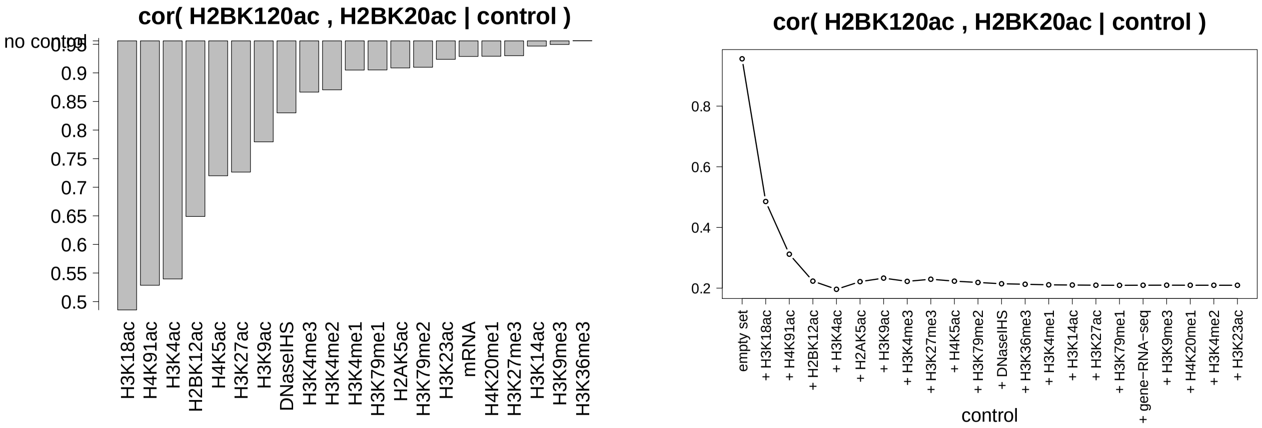

Figure S53: **Explaining away the pair H2BK120ac-H2BK20ac. Left:** Cor(H2BK120ac, H2BK20ac$|Z$), when Z is a single variable. **Right:** Cor(H2BK120ac, H2BK20ac$|Z$), when Z grows, as explained in Main Document "Section From correlations to partial correlations: explaining away".

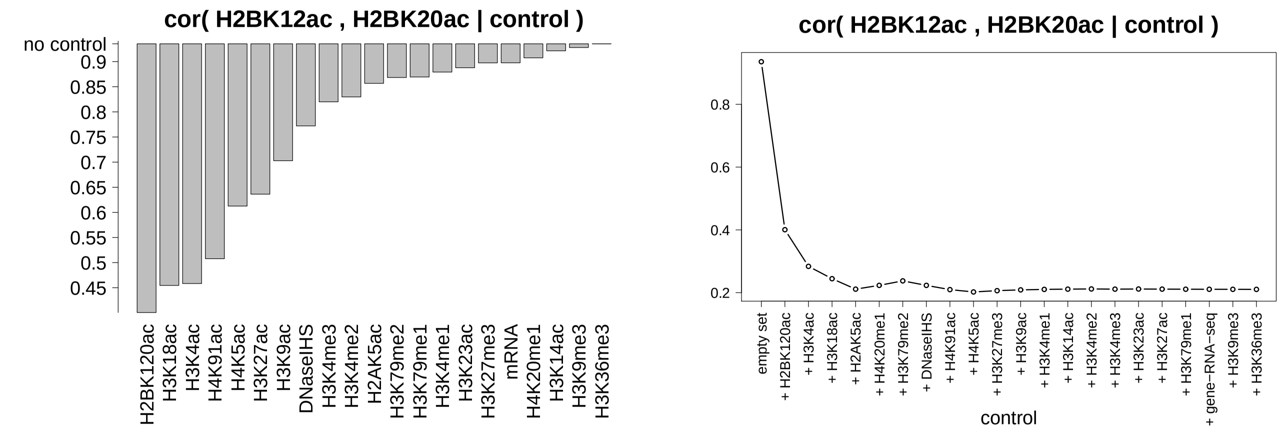

Figure S54: **Explaining away the pair H2BK12ac-H2BK20ac. Left:** Cor(H2BK12ac, H2BK20ac|Z), when Z is a single variable. **Right:** Cor(H2BK12ac, H2BK20ac|Z), when Z grows, as explained in Main Document "Section From correlations to partial correlations: explaining away".

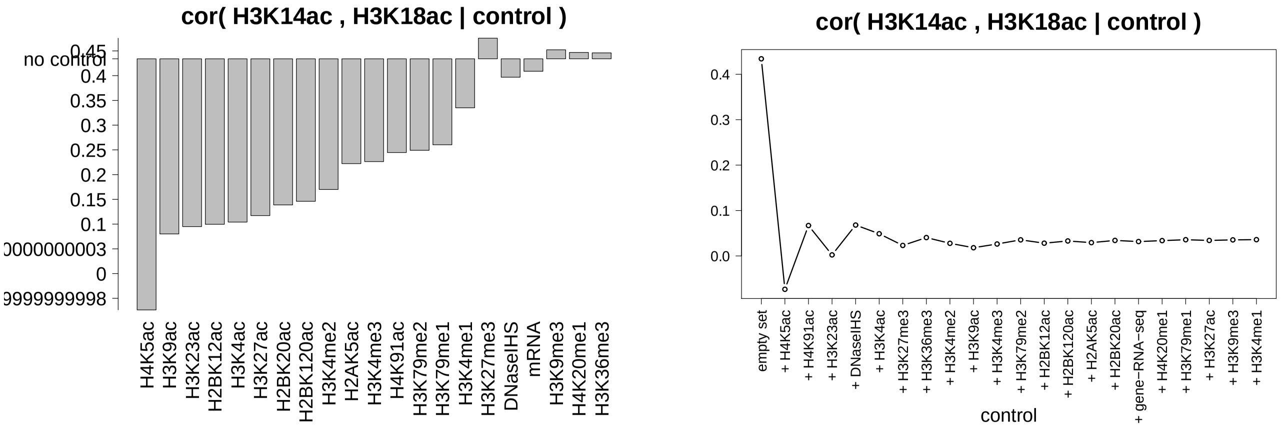

Figure S55: **Explaining away the pair H3K14ac-H3K18ac. Left:** Cor(H3K14ac, H3K18ac|Z), when Z is a single variable. **Right:** Cor(H3K14ac, H3K18ac|Z), when Z grows, as explained in Main Document "Section From correlations to partial correlations: explaining away".

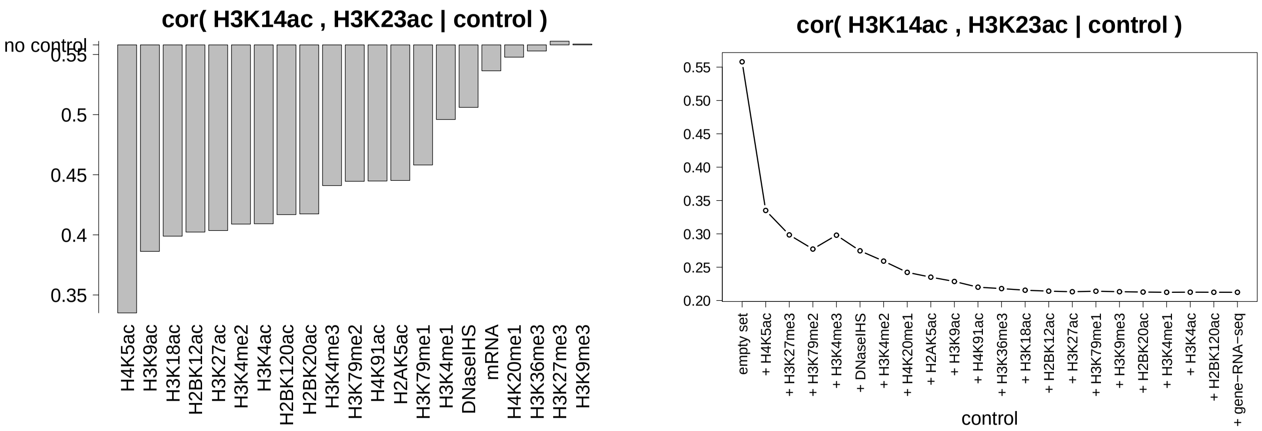

Figure S56: **Explaining away the pair H3K14ac-H3K23ac. Left:** Cor(H3K14ac, H3K23ac$|Z$), when Z is a single variable. **Right:** Cor(H3K14ac, H3K23ac$|Z$), when Z grows, as explained in Main Document "Section From correlations to partial correlations: explaining away".

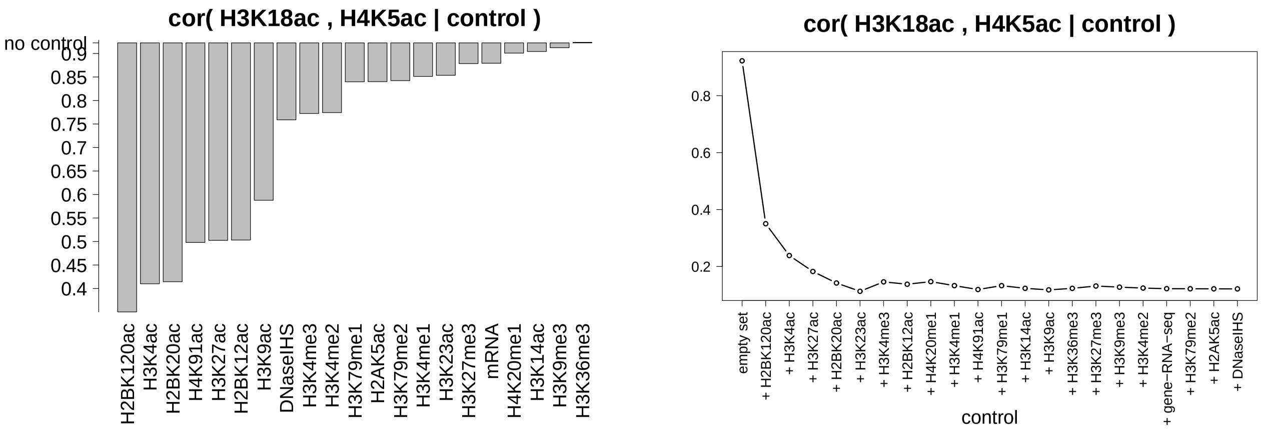

Figure S57: **Explaining away the pair H3K18ac-H4K5ac. Left:** Cor(H3K18ac, H4K5ac$|Z$), when Z is a single variable. **Right:** Cor(H3K18ac, H4K5ac$|Z$), when Z grows, as explained in Main Document "Section From correlations to partial correlations: explaining away".

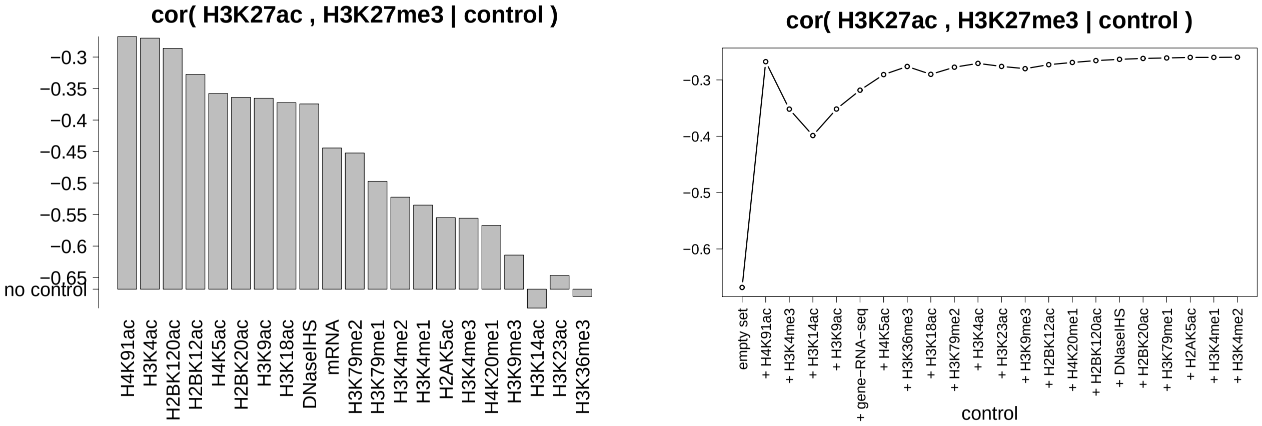

Figure S58: **Explaining away the pair H3K27ac-H3K27me3. Left:** Cor(H3K27ac, H3K27me3|Z), when Z is a single variable. **Right:** Cor(H3K27ac, H3K27me3|Z), when Z grows, as explained in Main Document "Section From correlations to partial correlations: explaining away".

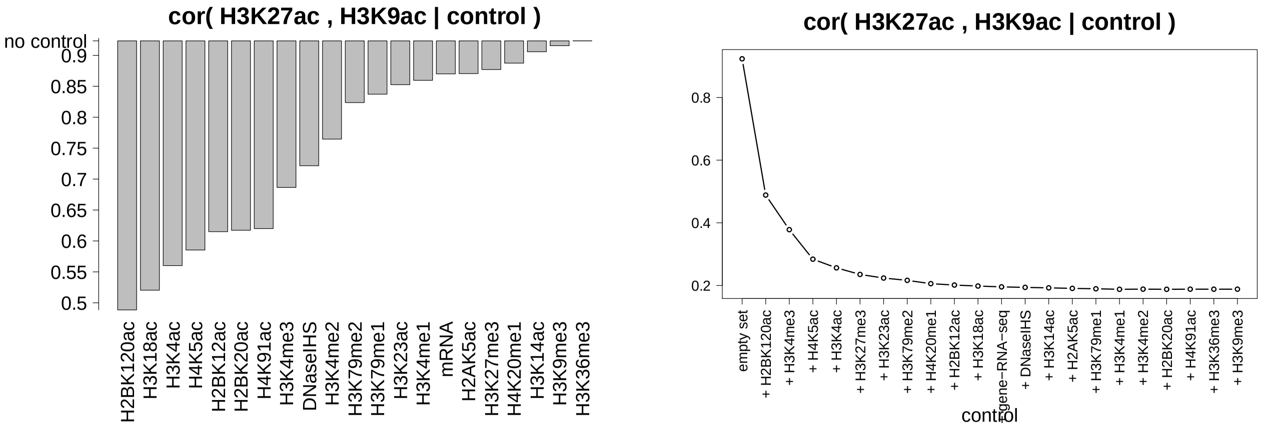

Figure S59: **Explaining away the pair H3K27ac-H3K9ac. Left:** Cor(H3K27ac, H3K9ac|Z), when Z is a single variable. **Right:** Cor(H3K27ac, H3K9ac|Z), when Z grows, as explained in Main Document "Section From correlations to partial correlations: explaining away".

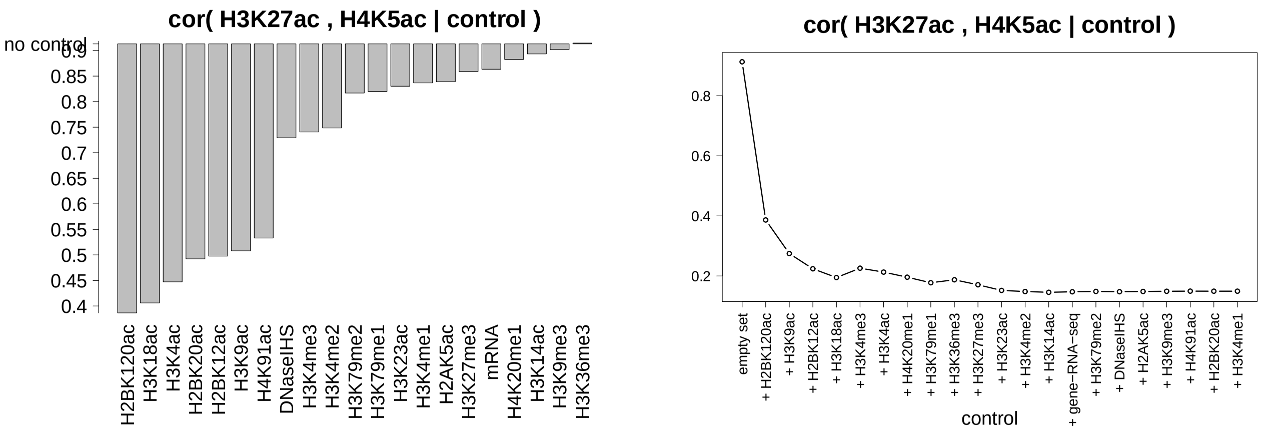

Figure S60: **Explaining away the pair H3K27ac-H4K5ac. Left:** $\mathrm{Cor}(\mathrm{H3K27ac}, \mathrm{H4K5ac}|Z)$, when Z is a single variable. **Right:** $\mathrm{Cor}(\mathrm{H3K27ac}, \mathrm{H4K5ac}|Z)$, when Z grows, as explained in Main Document "Section From correlations to partial correlations: explaining away".

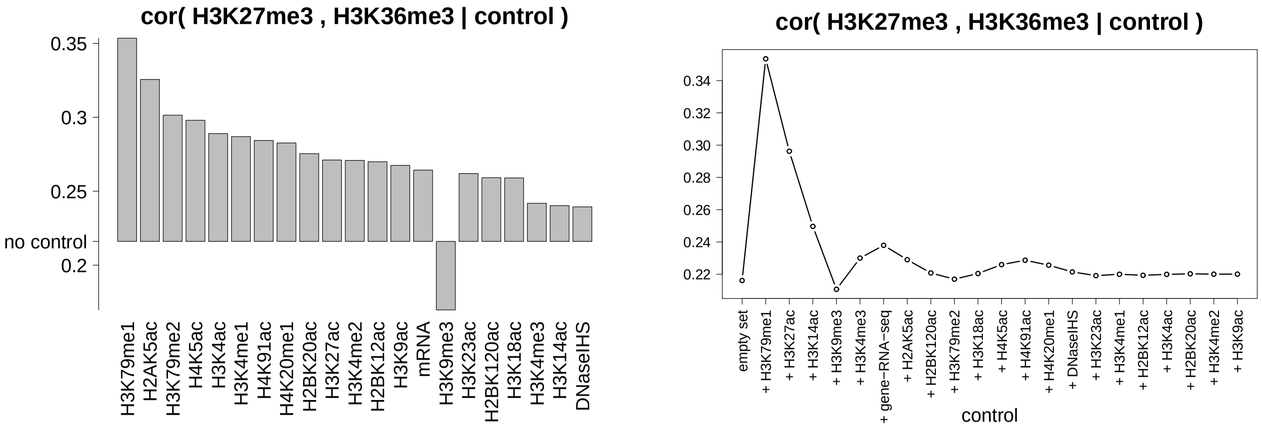

Figure S61: **Explaining away the pair H3K27me3-H3K36me3. Left:** $\mathrm{Cor}(\mathrm{H3K27me3}, \mathrm{H3K36me3}|Z)$, when Z is a single variable. **Right:** $\mathrm{Cor}(\mathrm{H3K27me3}, \mathrm{H3K36me3}|Z)$, when Z grows, as explained in Main Document "Section From correlations to partial correlations: explaining away".

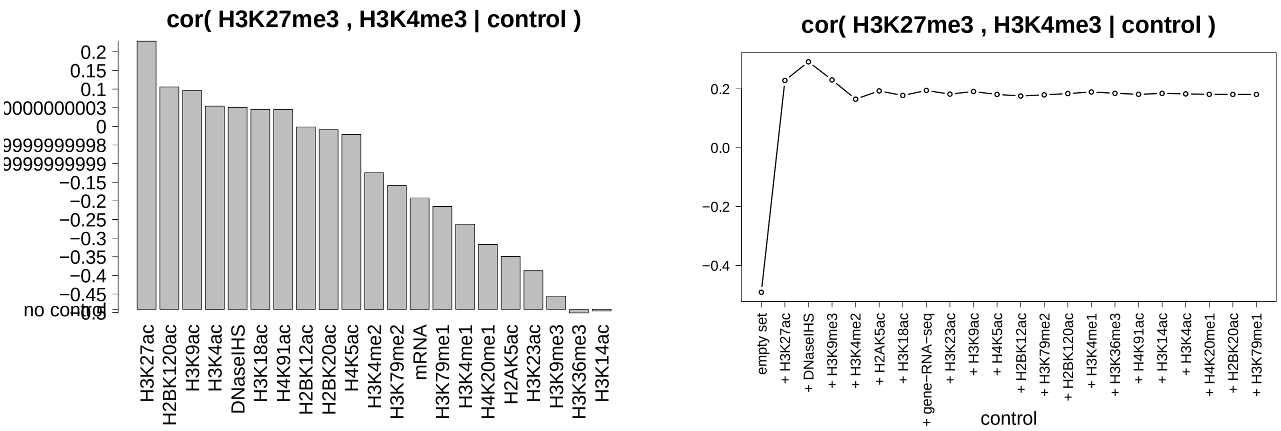

Figure S62: **Explaining away the pair H3K27me3-H3K4me3. Left:** Cor(H3K27me3, H3K4me3$|Z$), when Z is a single variable. **Right:** Cor(H3K27me3, H3K4me3$|Z$), when Z grows, as explained in Main Document "Section From correlations to partial correlations: explaining away".

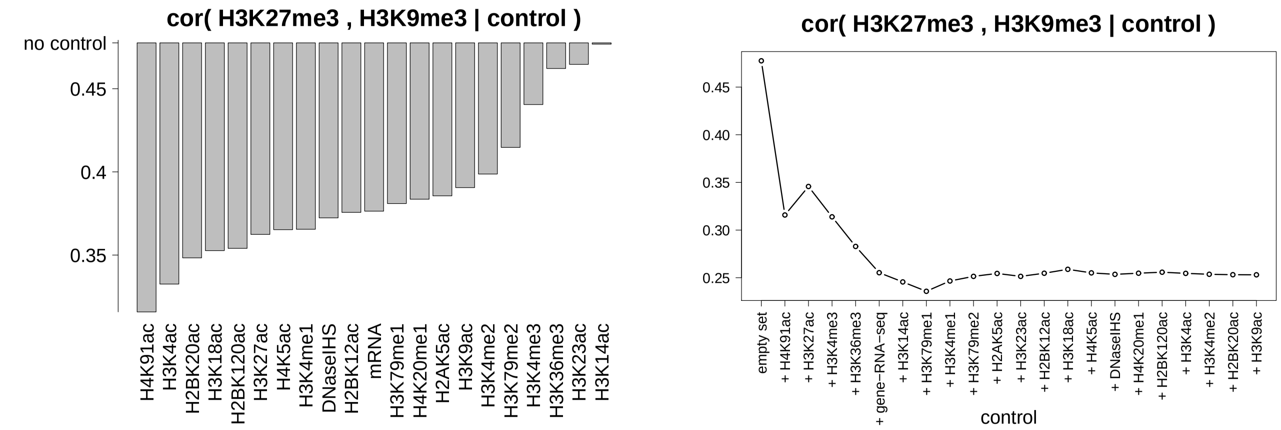

Figure S63: **Explaining away the pair H3K27me3-H3K9me3. Left:** Cor(H3K27me3, H3K9me3$|Z$), when Z is a single variable. **Right:** Cor(H3K27me3, H3K9me3$|Z$), when Z grows, as explained in Main Document "Section From correlations to partial correlations: explaining away".

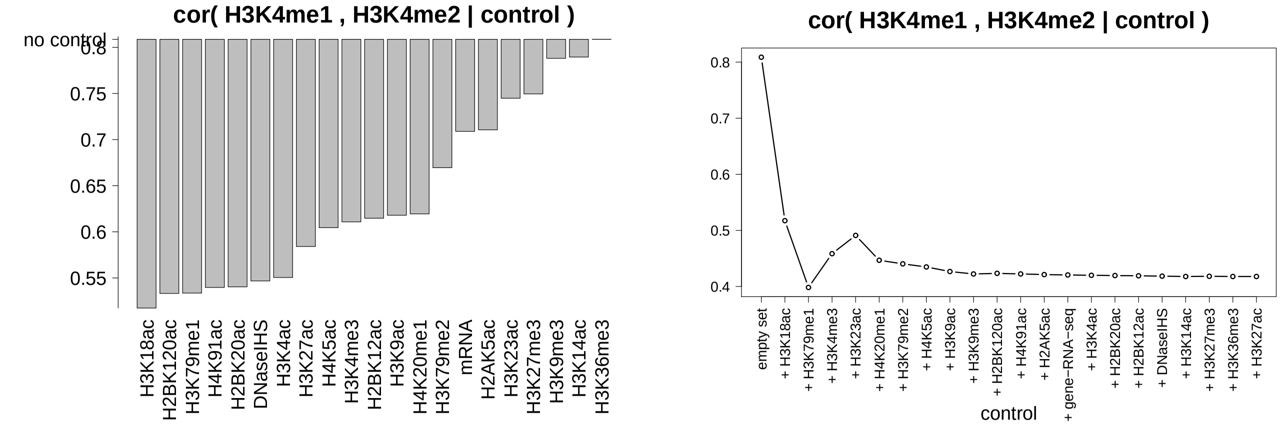

Figure S64: **Explaining away the pair H3K4me1-H3K4me2. Left:** Cor(H3K4me1, H3K4me2|Z), when Z is a single variable. **Right:** Cor(H3K4me1, H3K4me2|Z), when Z grows, as explained in Main Document "Section From correlations to partial correlations: explaining away".

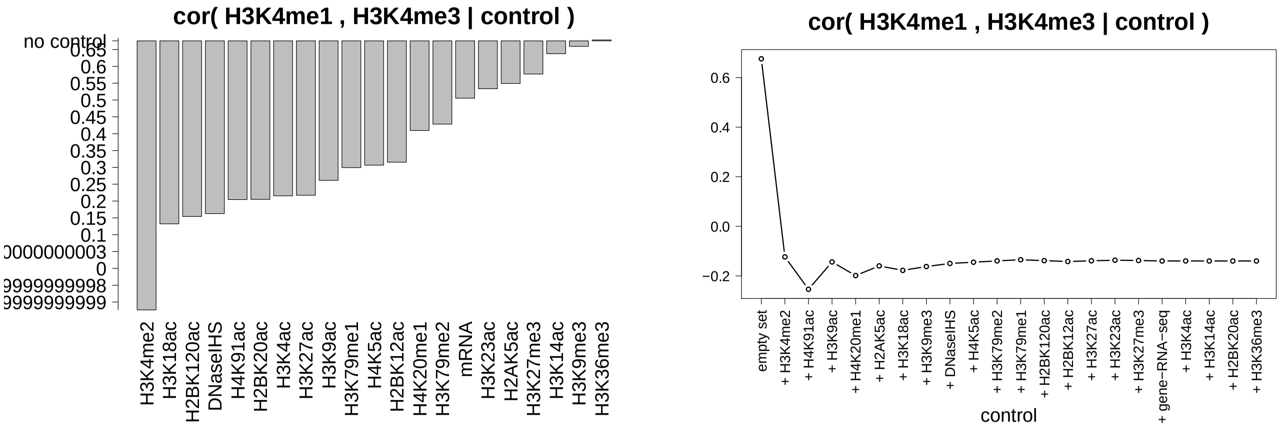

Figure S65: **Explaining away the pair H3K4me1-H3K4me3. Left:** Cor(H3K4me1, H3K4me3|Z), when Z is a single variable. **Right:** Cor(H3K4me1, H3K4me3|Z), when Z grows, as explained in Main Document "Section From correlations to partial correlations: explaining away".

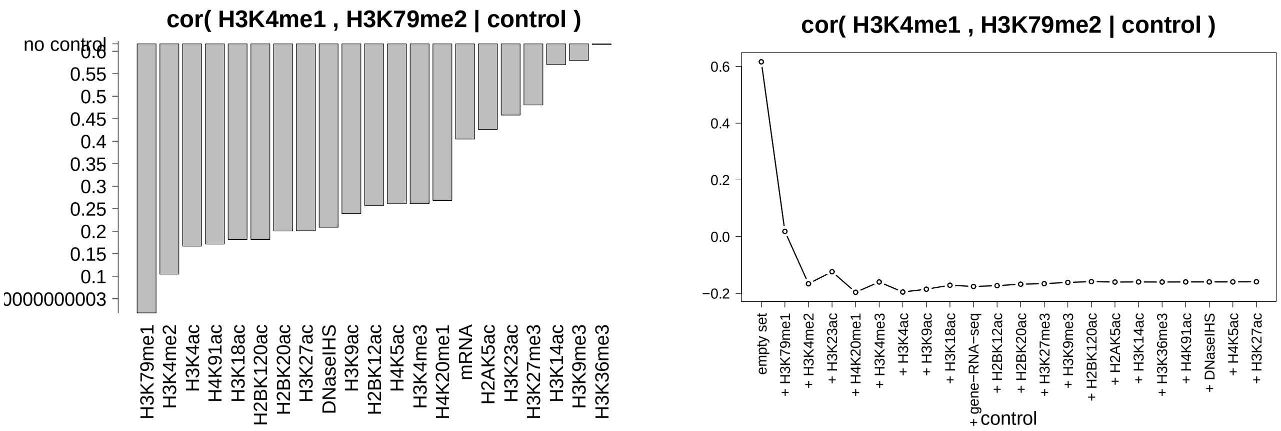

Figure S66: **Explaining away the pair H3K4me1-H3K79me2. Left:** Cor(H3K4me1, H3K79me2|Z), when Z is a single variable. **Right:** Cor(H3K4me1, H3K79me2|Z), when Z grows, as explained in Main Document "Section From correlations to partial correlations: explaining away".

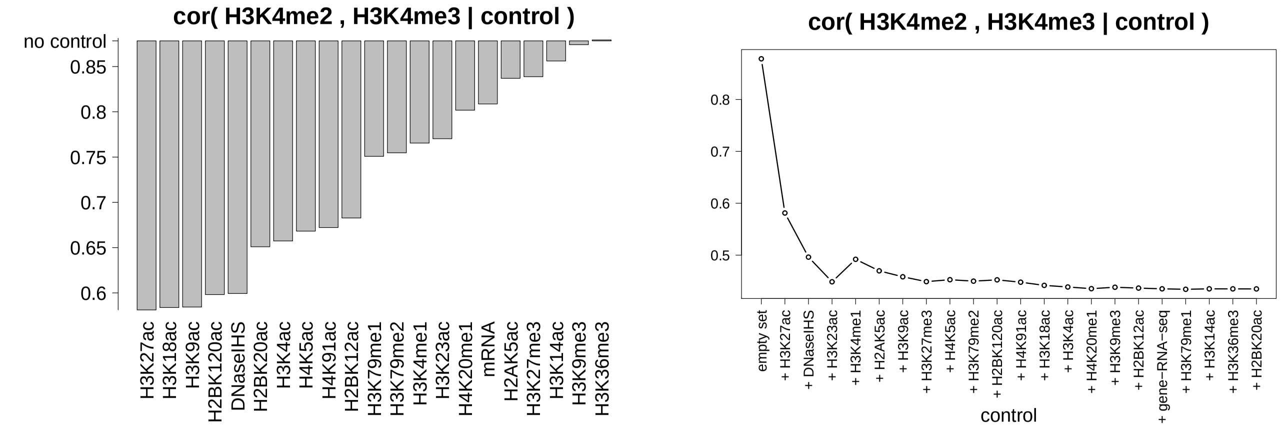

Figure S67: **Explaining away the pair H3K4me2-H3K4me3. Left:** Cor(H3K4me2, H3K4me3|Z), when Z is a single variable. **Right:** Cor(H3K4me2, H3K4me3|Z), when Z grows, as explained in Main Document "Section From correlations to partial correlations: explaining away".

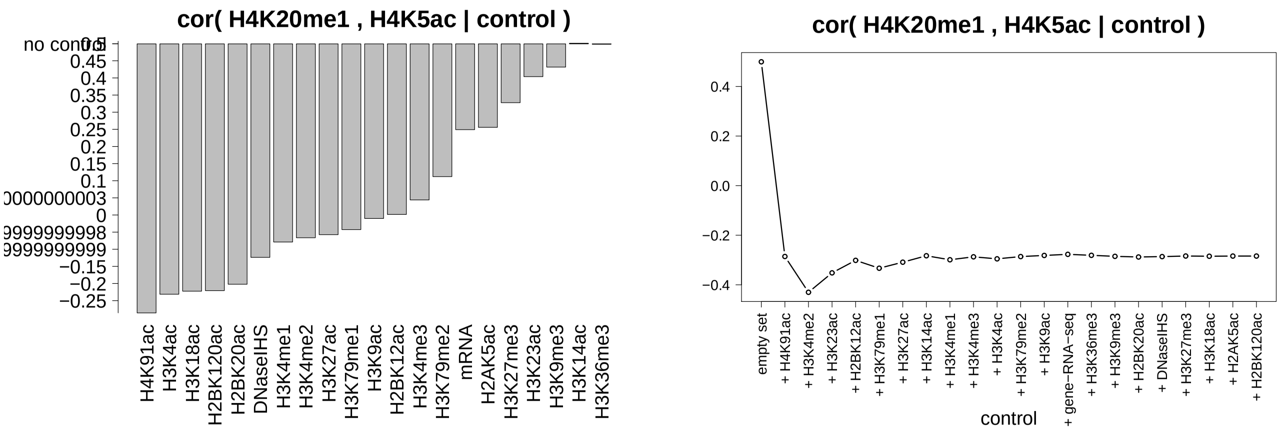

Figure S68: **Explaining away the pair H4K20me1-H4K5ac. Left:** $\mathrm{Cor}(\mathrm{H4K20me1}, \mathrm{H4K5ac}|Z)$, when Z is a single variable. **Right:** $\mathrm{Cor}(\mathrm{H4K20me1}, \mathrm{H4K5ac}|Z)$, when Z grows, as explained in Main Document "Section From correlations to partial correlations: explaining away".

References

[1] Chepelev I, Wei G, Tang Q, Zhao K (2009) Detection of single nucleotide variations in expressed exons of the human genome using rna-seq. Nucleic Acids Research 37: e106.

[2] Bernstein B, Stamatoyannopoulos J, Costello J, Ren B, Milosavljevic A, et al. (2010) The NIH Roadmap Epigenomics mapping consortium. Nature Biotechnology 28: 1045-1048.

[3] Schones DE, Cui K, Cuddapah S, Roh TY, Barski A, et al. .

[4] Wang Z, Zang C, Rosenfeld JA, Schones DE, Barski A, et al. (2008) Combinatorial patterns of histone acetylations and methylations in the human genome. Nature Genetics 40: 897-903.

[5] Barski A, Cuddapah S, Cui K, Roh TY, Schones DE, et al. (2007) High-Resolution Profiling of Histone Methylations in the Human Genome. Cell 129: 823-837.

[6] Boyle A, Song L, Lee B, London D, Keefe D, et al. (2011) High-resolution genome-wide in vivo footprinting of diverse transcription factors in human cells. Genome Research 21: 456-464.

[7] The ENCODE Project Consortium (2004) The ENCODE (ENCylopedia Of DNA Elements) Project. Science 306: 636-640.

[8] DNA Nexus (2012). Sequence Read Archive. URL `http://sra.dnanexus.com/experiments/`.

[9] San Diego Epigenome Center (2012). Antibody Validation. URL `http://epigenome.ucsd.edu/antibodies.html`.

[10] Harvard Medical School (2012). Antibody Validation Database. URL `http://compbio.med.harvard.edu/antibodies/sources/`.

[11] Fisher RA (1915) Frequency distribution of the values of the correlation coefficient in samples of an indefinitely large population. Biometrika 10: 507-521.

[12] Karlic R, Chung HR, Lasserre J, Vlahovicek K, Vingron M (2010) Histone modification levels are predictive for gene expression. Proceedings of the National Academy of Sciences of the United States of America 107: 2926-2931.

[13] Margolin AA, Nemenman I, Basso K, Wiggins C, Stolovitzky G, et al. (2006) ARACNE: an algorithm for the reconstruction of gene regulatory networks in a mammalian cellular context. BMC Bioinformatics 7: S7.

[14] Saxonov S, Berg P, Brutlag DL (2006) A genome-wide analysis of CpG dinucleotides in the human genome distinguishes two distinct classes of promoters. Proceedings of the National Academy of Sciences of the United States of America 103: 1412-1417.
